# Supplementary material for: Protocol for a definitive randomised controlled trial and economic evaluation of a community-based rehabilitation programme following hip fracture: fracture in the elderly multidisciplinary rehabilitation—phase III (FEMuR III)
Source: BMJ Open. 2020 Oct 16;10(10):e039791. doi: 10.1136/bmjopen-2020-039791 (PMC7569930; doi:10.1136/bmjopen-2020-039791)
Supplement: Supplementary data [file bmjopen-2020-039791supp001.pdf]

Appendix 1 FEMuR III Trial Registration Data

Data category	Information
Registry and trial identification no.	ISRCTN28376407
Date of registration	23/11/2018
Funder	NIHR Evaluation, Trials and Studies Co-ordinating Centre (NETSCC); Grant code 16/167/09
Sponsor	University of Liverpool
Contact for public enquiries	LH email: femur3@liverpool.ac.uk
Scientific title	Fracture in the Elderly Multidisciplinary Rehabilitation - Phase III (FEMuR III): a definitive randomised controlled trial and economic evaluation of a community-based rehabilitation package following hip fracture
Acronym	FEMuR III
Countries of recruitment	United Kingdom
Health condition	Hip fracture
Intervention	Intervention comparator: Enhanced rehabilitation
	Control comparator: Usual care
Inclusion criteria	Aged 60 years or older
	Recent proximal hip fracture
	Surgical repair by replacement arthroplasty or internal fixation
	Living in own home prior to hip fracture
	Living and receiving rehabilitation from the NHS in the area covered by the trial centres
Exclusion criteria	Living in residential or nursing home prior to hip fracture
	Unable to understand English or Welsh
	Lacking mental capacity to give informed consent
Study design	Interventional
	Randomised controlled trial
	Treatment, education or self-management, psychological and behavioural, complex intervention, physical, rehabilitation
Recruitment start date	01/04/2019
Target sample size	446
Primary outcome	Nottingham Extended Activities of Daily Living scale
Secondary outcomes	EuroQol EQ-5D, Hospital Anxiety and Depression Scale, Abbreviated Mental Test Score, Falls Efficacy Scale – International, hip pain intensity, fear of falling, grip strength, short physical performance battery

Appendix 2 FEMuR III Patient Participant Information Sheet and Informed Consent Forms

Fracture in the Elderly Multidisciplinary Rehabilitation III

<<Local NHS Logo to go here>>

<Trust/Site address 1>
<Trust/Site address 2>
<Trust/Site address 3>
<postcode>
Tel: <telephone number>

Patient Participant Information Sheet

Contents

Contents	Page
You are invited to take part in FEMuR III	1
Why are we doing the FEMuR III study?	2
What is the Enhanced Rehabilitation Package?	2
Why have I been asked to take part?	2
What will I have to do if I take part?	2
Timeline of visits	3
How will I know which treatment I'm going to have?	3
What are the benefits and risks of taking part?	3
What are the alternatives for treatment?	3
Do I have to take part?	4
What happens if I change my mind?	4
Will my participation be kept confidential?	4
What will happen to the results of the study?	5
What if there is a problem?	5
Additional information	5
Additional information about future research	5
Contacts for further information	6
Consent Form	7

Important Contact Information

Thank you for taking the time to read this information sheet. We hope you will find this information helpful.

If you would like a large print version of this information sheet please ask your research team.

If you have any questions about this study please talk to your research team:

<Add contact details for PI/RN i.e., name and telephone number>

Website: Femur3study.co.uk

You are invited to take part in FEMuR III

Important things to know about FEMUR III:

- FEMuR III aims to compare a new enhanced rehabilitation package with standard NHS care for patients who have had surgery to repair a hip fracture.
- We are interested in the recovery of patients aged over 60 years old who lived independently before they suffered a hip fracture even if they were in receipt of personal care at home.
- Being part of the study means you will receive either standard care or standard care **plus** enhanced rehabilitation when you leave hospital.
- Standard care can vary but usually involves community-based physiotherapy. The enhanced rehabilitation package will provide additional physiotherapy, occupational therapy and some 'self-help' tools to aid recovery.
- You have been given this information sheet as you might be eligible to take part in this study.
- Before you decide whether or not to take part it is important for you to understand why the research is being done and what it will involve for you. Please take time to read the following information carefully and discuss it with friends or relatives if you wish.
- Taking part is voluntary. If you don't want to take part then you don't need to give a reason.
- If you have someone who provides you with help for most days of the week with activities of daily living or physical care, we would also like them to be involved in the study.
- Please ask a member of your clinical team if there is anything that is not clear, or if you would like more information.

FEMuR III Patient PISC V4.0 19/09/2019
IRAS Project ID: 246828
Page 1 of 8

Why are we doing the FEMuR III study?

Hip fracture is a common, major health problem in old age, especially for people with other health problems or who are frail. Some patients who suffer this type of fracture need surgery to repair it. They take a long time to recover, and others may not recover fully.

Once patients are discharged, the routine care they receive can vary depending on local NHS policy. Some may not find it as easy to live independently afterwards.

We have designed an enhanced rehabilitation package for patients who are recovering from this surgery, which is delivered in addition to standard NHS care. FEMuR III will compare the enhanced package with standard NHS care to see if it can improve recovery for patients.

What is the Enhanced Rehabilitation Package?

The enhanced rehabilitation package is made specifically for each patient and we think this should improve recovery. We think this package should work better if it includes physiotherapy (to help patients recover movement), occupational therapy (to help patients with activities associated with daily living) and also provides tools to help build confidence and mood.

The enhanced rehabilitation package we have designed involves additional rehabilitation at follow up visits. You will be given a workbook and a goal-setting diary to complete during the first few months of recovery.

In order to compare the enhanced rehabilitation package to standard NHS care we are asking 446 people to take part in our study. We will follow your progress in the 12 months after surgery and collect information from you during that time so we can see how you are. The information we collect will help us to see whether there is a difference between those people who have standard NHS treatment and those who receive standard NHS treatment **and** the enhanced rehabilitation package.

Why have I been asked to take part?

We are inviting you to take part in this study because you are a patient at one of the hospitals taking part and have recently had surgery to repair a hip fracture.

What will I have to do if I take part?

A member of the clinical team can talk to you in more detail about this study and you will be able to ask any questions that you have. If you have had all of your questions answered and are happy to take part then you will be asked to sign a consent form to confirm you want to take part. You will be given a copy of your consent form and this information sheet to keep.

This study is comparing standard NHS care with the enhanced rehabilitation package. Both of these will be tailored to individual patients so it is difficult for us to describe exactly what your rehabilitation will look like. However, the main differences between the two are that if you are receiving the enhanced rehabilitation package you will also:

- Be given a goal-setting diary to complete which we would like you to use to set yourself targets and track your progress through your rehabilitation.
- Be given a workbook containing information about hip fractures: what to expect during recovery, tips to aid recovery, and useful contacts if you would like more information.
- Receive up to six community/home-based therapy sessions **in addition** to any provided by the NHS.

If you do decide to take part and have given your consent, we will ask you to complete some questionnaires and do a grip-strength test so we can get some information about how you are feeling both mentally and physically. This will allow us to see how you are recovering over time. The grip – strength test uses a special piece of equipment to measure this and will be carried out at home with a researcher.

We will arrange an appointment with you around 4 months after you have started the study to see how you are getting on. This visit may take place at your home or in a community hospital depending on what is best with you. At the visit, we will ask you to complete the same questionnaires and do some basic physical tests so we can see how you are recovering. At around 12 months after you started the study we will arrange one more visit to complete the questionnaires and physical tests again so we can track your progress. The physical tests are things you do every day. For example, the researcher will ask you to sit and stand up to five times (if you are able) and will record your progress.

We may also telephone you soon after the 4-month visit to ask some questions about how you are finding being involved in the study. This will give you a chance to give some feedback on your experiences and your views on the care you are receiving. Not all patients will receive a phone call, we aim to call 60 patients who have agreed to take part. We will offer you a £30 shopping voucher for your time and inconvenience in taking part in the telephone interview.

Timeline of visits

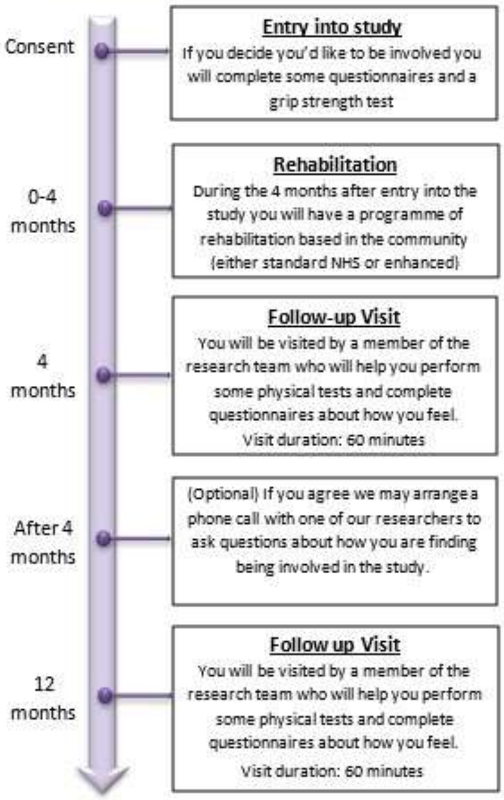

How will I know which treatment I'm going to have?

In the FEMuR III study patients will be split into two groups at random:

- One group will receive standard NHS care after discharge
- The other group will receive the enhanced rehabilitation package alongside standard NHS care after discharge

We use a computer programme that puts patients 'at random' into one of the groups – you might hear this described as 'randomisation' or 'random allocation', but they all mean the same thing. Neither you nor your doctor chooses which group you are in.

In this study you are equally likely to be in the group receiving standard NHS care as you are in the group receiving the enhanced package. Your healthcare team will let you know which group you are in as soon as possible.

What are the benefits and risks of taking part?

We are not sure whether standard NHS care or the enhanced rehabilitation package is best, but we anticipate that both will aid your recovery following surgery.

We do not foresee any significant risks involved in taking part in FEMuR III. All of the physical exercises suggested are used in normal rehabilitation after hip fracture and will be supervised by trained healthcare professionals to minimise any risk. The enhanced rehabilitation package will take up more of your time due to additional therapy sessions and having to complete the diary.

We hope that the results from the study will help doctors, therapists, patients and their carers in the future when making decisions about treatment.

What are the alternatives for treatment?

Patients recovering from hip fracture repair will get standard NHS treatment, though this may vary in different areas. In this study every patient will get standard NHS treatment even if they are in the enhanced rehabilitation group. Currently, there are no other alternative treatment programmes available.

Do I have to take part?

No, taking part is voluntary. It is up to you to decide whether or not you want to take part. If you choose to take part you can also choose to stop at any time without giving a reason. The standard of care you receive now or in the future will be the same whether you take part or not.

What happens if I change my mind?

If at any point you decide to stop taking part in the study you will receive the treatment and follow up usually offered in your area. The standard of care you receive will remain the same if you decide to stop taking part. If you do decide to stop taking part we will ask you if you would like to either:

- continue to complete follow up visits for the study
- stop taking part with no more study visits.

We will use any study information collected up until the time you stop taking part.

Will my participation be kept confidential?

Yes. All information collected about you during the course of the study will be handled according to relevant ethical and legal requirements. Your personal information will be kept strictly confidential and will only be accessed by people working on the study, or working to ensure the study is being run correctly.

You will be given a study number, which will be used along with your initials to identify you on each paper form. Your full name and date of birth, postcode, contact details and NHS number will be included on your consent form and a copy of this will be sent to the study team at the coordinating centre for the study, the Liverpool Clinical Research Centre (LCTC.) There may be instances (depending on your local NHS,) when a copy of your contact details will need to be sent to other locations or Universities within your local area to arrange your follow up visits. Only members of the FEMUR III team will be given access to your contact details, they will be held securely and destroyed after your final follow-up. Every effort will be made to ensure that any further information about you that leaves the hospital will have your name removed so that you cannot be recognised from it. This information will usually be removed by a member of the study team at your hospital/community care, but may also be removed by study team members at the LCTC upon receipt. We will also ask for your telephone number so that we can contact you for the telephone interviews during the study. Your telephone number will not be used for anything else without your consent.

With your consent, we will send a letter to your GP to let them know you are taking part and we will use your NHS number and postcode to access data about your use of health services (for example your hospital admissions).

The University of Liverpool is the sponsor for this study based in the United Kingdom. The University of Liverpool along with Bangor University will be using information from you and your medical records in order to undertake this study and will act as joint data controllers for this study. This means that both joint data controllers are responsible for looking after your information and using it properly. The University of Liverpool will keep identifiable information about you for a maximum of 25 years after the study has finished. Arrangements for confidential destruction will then be made.

Details about the use of health services (health economics) will be collected in this study. Information will be obtained from your hospital finance department and NHS Digital. Health economics researchers at the Centre for Health Economics and Medicines Evaluation (part of Bangor University), who are part of the study team, will use these data to calculate the overall costs of care. Data will be provided to the study team by NHS Digital and in order to obtain this, your NHS number will be securely transferred to NHS Digital by the CTRC using an encrypted electronic transfer system.

We would also like to collect information regarding your therapy sessions from electronic data from Therapy Management systems. This data will include date of sessions, location of sessions and activities completed during the session. This data will help us to see how many therapy sessions you have completed during the study. This data will be extracted from Therapy Management systems by NHS IT personnel and transferred using an encrypted electronic transfer system to a researcher at the University of Liverpool.

Your rights to access, change or move your information are limited, as we need to manage your information in specific ways in order for the research to be reliable and accurate. If you withdraw from the study, we will keep the information about you that we have already obtained. To safeguard your rights, we will use the minimum personally-identifiable information possible.

You can find out more about how we use your information in the "**How we use your information**" section on the study website *Femur3study.co.uk*

Your NHS hospital and/or community health team will collect information from you and your medical records for this research study in accordance with our instructions.

Your NHS hospital and/or community health team will use your name, NHS number and contact details to contact you about the research study, and make sure that relevant information about the study is recorded for your care, and to oversee the quality of the study. Individuals from the team and regulatory organisations may look at your medical and research records to check the accuracy of the research study. Your NHS hospital and/or community health team will pass these details to the University of Liverpool along with the information collected from you and your medical records. The only people in the University of Liverpool who will have access to information that identifies you will be people who need to confirm your participation in the study, to contact you after 4 months to ask you questions about taking part in this study or audit the data collection process. The people who analyse the information will not attempt to identify you or find out your name, NHS number or contact details.

Your NHS hospital and/or community health team will keep identifiable information about you from this study for up to a maximum of 25 years after the study has finished.

Additional information about future research:

When you agree to take part in a research study, the information about your health and care may be provided to researchers running other research studies in this organisation and in other organisations. These organisations may be universities, NHS organisations or companies involved in health and care research in this country or abroad. Your information will only be used by organisations and researchers to conduct research in accordance with the UK Policy Framework for Health and Social Care Research.

This information will not identify you and will not be combined with other information in a way that could identify you. The information will only be used for the purpose of health and care research, and cannot be used to contact you or to affect your care. It will not be used to make decisions about future services available to you, such as insurance.

What will happen to the results of the study?

We want the results of the study to be presented at conferences and published in medical journals so that we can explain to the medical, nursing and therapies' community what our research results have shown. You will not be identified in any publication or presentations.

What if there is a problem?

If you have a concern about any aspect of this study, you should ask to speak with one of your research team who will do their best to answer your questions.

If at any time during the study you feel distressed or anxious please speak to your research team, a therapist or contact your GP.

If you wish to make a formal complaint, you can do this through the NHS Complaints Procedure. Details can be obtained from your hospital.

Every care will be taken in the course of this clinical study. However, in the unlikely event that you are harmed by taking part in this research project, compensation may be available and you may have to pay your related legal costs. The University of Liverpool holds insurance against claims from participants for harm caused by their participation in this clinical trial. Participants may be able to claim compensation if they can prove that the University of Liverpool has been negligent. However, the NHS organisation that has provided your treatment has a duty of care to you, whether or not you agree to participate in the study, and the study sponsor accepts no liability for negligence on the part of your NHS organisation's employees. However, if you are harmed, and this is due to someone's negligence in the NHS, then you may have grounds for a legal action for compensation against the NHS organisation providing your treatment, but you may have to pay for your legal costs. The normal NHS complaints procedures should be available to you.

Additional information

The University of Liverpool is responsible for managing this study; they have asked that the day to day running of the study is carried out by the Liverpool Clinical Research Centre (LCTC,) part of the University of Liverpool. Additional support is provided by health economics researchers from the Centre for Health

Economics and Medicines Evaluation, part of Bangor University, and other researchers from participating universities (the study team).

This study is funded by the National Institute for Health Research's Health Technology Assessment Programme (ref: 16/167/09).

The study has been reviewed by the National Institute for Health Research (NIHR), Health Research Authority and the National Research Ethics Service Committee. Tyne and Wear South Ethics Committee has reviewed the study and given approval for it to take place.

The views expressed are those of the author(s) and not necessarily those of the NHS, the NIHR or the Department of Health.

Thank you for reading this information sheet.

Contacts for further information

If you would like more information or have any questions about the FEMuR III study please talk to:

Principal Investigator: <PI name to go here>

Research Nurse: <RN name to go here>

Telephone: <Hospital contact number to go here>

Or visit the website: Femur3study.co.uk

If you wish to discuss the study with someone independent of the research team you can contact the local NHS Patient Advice and Liaison Service (PALS) or local equivalent on: <Local PALS or equivalent telephone number to go here>

<<Local NHS Logo to go here>>

Fracture in the
Elderly
Multidisciplinary
Rehabilitation III

<Trust/Site address 1>
<Trust/Site address 2>
<Trust/Site address 3>
<postcode>
Tel: <telephone number>

Patient Participant Consent Form

To be completed by the Researcher:			
Site Name:			
Participant Study Number			
Patient Initials:		Participant DOB:	/ /

**Once you have read and understood each statement please enter your initials in each box**	**Initial**
Example: I confirm that I have read and understand the Participant Information Sheet.	JS
1. I have read and understood the information sheet for this study. I have had the opportunity to ask questions and have had these answered satisfactorily.	
2. I understand that participation is voluntary and that I am free to withdraw from the study at any time, without giving a reason, and without my care or legal rights being affected. I understand that in some cases further information about any unwanted effects of treatment may need to be collected by the study team.	
3. I understand that my data will be retained for a maximum of 25 years at site and by the Liverpool Clinical Trials Centre (LCTC,) part of the University of Liverpool and that they will be stored in a confidential manner.	
4. I give permission for a copy of my consent form which will include my name and date of birth, postcode and NHS number to be sent to the members of the FEMUR III research team (where it will be kept in a secure location), to allow confirmation that my consent was given.	
5. I understand that relevant sections of my medical notes and any data collected during the study may be looked at by authorised individuals from the study team and those listed under "Additional information" (NHS organisation, sponsor and regulatory authorities). I give permission for these individuals to have access to my records.	
6. I agree to allow information or results arising from this study to be used in future healthcare and/or medical research in a pseudo-anonymised form.	
7. I agree to my GP being informed of my participation in the study.	
8. I agree for my data on NHS hospital admissions and treatment to be collected for the purpose of this study and understand this will include both routine paper and electronic NHS health care records covering the study period for health economic analysis.	
9. I agree for my routine electronic data from Therapy Management systems to be collected for the purpose of this study and understand that this will include both routine paper and electronic NHS health care records covering the study period for qualitative research purposes.	
10. I agree to take part in the above study.	
**The statements below are OPTIONAL *(you can still participate even if you only agree to the statements above)*:**	
11. I agree that I may be contacted in the future in relation to this or other related studies.	

FEMuR III Patient PISC V4.0 19/09/2019
IRAS Project ID: 246828

Page 7 of 8

12. I agree to being contacted by a study researcher to conduct a qualitative interview and for the interview to be recorded.<br>(if you agree to this statement provide your details below):<br>Telephone number:		☐

Your **full** name (please print):			
Your signature:		Date:	
To be completed by a witness only if participant is unable to sign the consent form:			
Witness **full** name (please print):			
Witness signature:		Date:	

To be completed by the Researcher (on the same day after participant has completed the form):			
Researcher **full** name (please print):			
Researcher signature:		Date:	
Additional details required for health economic analysis:			
Participant postcode:			
Participant NHS Number:			

Appendix 3 FEMuR III Carer Participant Information Sheet and Informed Consent Forms

Fracture in the
Elderly
Multidisciplinary
Rehabilitation III

<<Local NHS Logo to go here>>

<Trust/Site address 1>
<Trust/Site address 2>
<Trust/Site address 3>
<postcode>
Tel: <telephone number>

Carer Participant Information Sheet

Contents	Page
You are invited to take part in FEMuR III	1
Why are we doing the FEMuR III study?	2
What is the Enhanced Rehabilitation Package?	2
Why have I been asked to take part?	2
What will I have to do if I take part?	2
Timeline of visits	3
How will I know which treatment I'm going to have?	3
What are the benefits and risks of taking part?	3
What are the alternatives for treatment?	3
Do I have to take part?	4
What happens if I change my mind?	4
Will my participation be kept confidential?	4
What will happen to the results of the study?	5
What if there is a problem?	5
Additional information	5
Contacts for further information	6
Consent Form	7

Important Contact Information

Thank you for taking the time to read this information sheet. We hope you will find this information helpful.

If you have any questions about this study please talk to your research team:

If you would like a large print version of this information sheet please ask your research team.

<Add contact details for PI/RN i.e., name and telephone number>

Website: Femur3study.co.uk

You are invited to take part in FEMuR III

Important things to know about FEMUR III:

- FEMuR III aims to compare a new enhanced rehabilitation package with standard NHS care for patients who have had surgery to repair a hip fracture.
- After surgery, some patients take a long time to recover, and others may not recover fully.
- Additional care is often provided by members of the family or close friends, and usually involves helping hip fracture patients with activities associated with daily living or physical care.
- You have been given this information sheet as you are a carer of someone who is potentially eligible to take part.
- Before you decide whether or not to take part it is important for you to understand why the research is being done and what it will involve for you. Please take time to read the following information carefully and discuss it with friends or relatives if you wish.
- Taking part is voluntary. If you don't want to take part then you don't need to give a reason.
- Please ask a member of the clinical team if there is anything that is not clear, or if you would like more information.

Why are we doing the FEMuR III study?

The FEMuR III study hopes to collect information to help provide evidence about the best way to treat patients who are recovering from hip repair surgery after a fracture. Hip fractures are common and often need to be surgically repaired. Once patients are discharged, the routine care they receive can vary depending on local

NHS policy and they often require additional help at home from family members or friends.

FEMuR III will compare an enhanced rehabilitation programme we have designed against the normal care given by the NHS. We want to see if one offers any additional benefit over the other. To do this, we are asking 446 patients and their carers to take part. We will follow progress in the 12 months after surgery and collect information from you during that time so we can see how you both are.

What is the Enhanced Rehabilitation Package?

The enhanced rehabilitation package is made specifically for each patient and we think this should improve recovery. The enhanced rehabilitation package mixes extra therapy with self-help tools which aim to help patients improve aspects of their own recovery e.g., build confidence in trying exercises by themselves. We also hope that this will help the people caring for hip fracture patients by reducing the level of care they need to provide as patients may be able to recover independence quicker.

The enhanced rehabilitation package we have designed involves additional rehabilitation sessions and patients will be given a workbook and a goal-setting diary to complete during the first few months of recovery. Carers will be given questionnaires to complete so we can look at how much help you are providing and how you are managing.

In order to compare the enhanced rehabilitation package with standard NHS care we are asking 446 people aged 60 and over, who have had hip repair surgery, to take part in our study. We will follow their progress in the 12 months after surgery and collect information from them and from you (as their carer) during that time so we can see how you both are. The information we collect will help us to see whether there is a difference between those people who have standard NHS treatment and those who receive standard NHS treatment and the enhanced rehabilitation package.

Why have I been asked to take part?

We are inviting you to take part in this study because you are a carer of a patient who has recently had surgery to repair a hip fracture at one of the hospitals taking part

What will I have to do if I take part?

A member of the clinical team will talk to you first in more detail and you will be able to ask any questions that you have. If you have had all of your questions answered and are happy to take part then you will be asked to sign a consent form to confirm you want to participate. You will be given a copy of the consent form and the information sheet to keep.

If you do decide to take part and have given your consent, we will ask you to complete some questionnaires so we can see how you are feeling. These may take around 15 minutes to complete.

We will arrange an appointment with your friend/relative around 4 months after surgery to see how they are getting on. This will involve a member of the research team coming out to visit your friend/relative and we would like to ask that you be present too. At the visit, we will ask you to complete the same questionnaires that you completed at the start, so that we can see if there is any difference in how you are feeling. At around 12 months after the surgery we will arrange one more visit to complete the questionnaires again so that we can see if anything has changed.

We may also telephone you after the 4-month visit to ask some questions about how you are finding being involved in the study. This will give you a chance to give some feedback on your experiences as well as your views on the care your friend/relative is receiving. Not all carers will receive a phone call. We aim to call 30 carers who have agreed to take part. We will offer you a £30 shopping voucher for your time and inconvenience in taking part in the telephone interview.

Timeline of visits

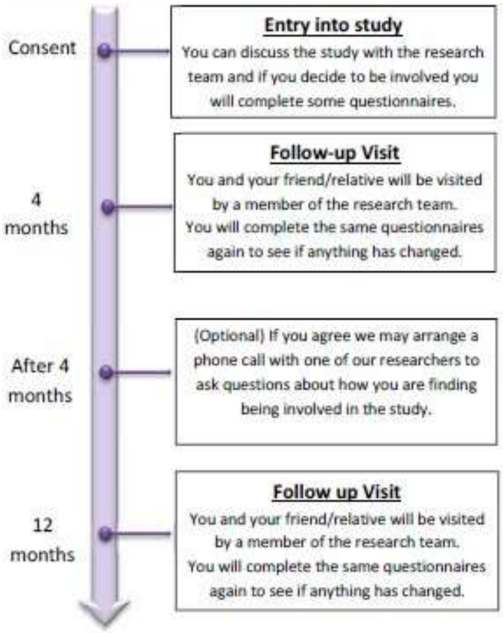

How will I know which treatment my friend/relative going to have?

In the FEMuR III study patients will be split into two groups at random:

- One group will receive normal NHS care
- The other group will receive the enhanced rehabilitation package

In the FEMuR III study participants are equally as likely to be in the group receiving normal NHS care as in the group receiving the enhanced programme. The healthcare team will let you know which group your friend/relative is in as soon as possible.

What are the benefits and risks of taking part?

We are not sure whether routine NHS care or the enhanced therapy programme is best but we anticipate that both will aid patient recovery following surgery.

We do not foresee any significant risks involved in taking part in FEMuR III, although the assessments will take up some time. It is also possible that the enhanced rehabilitation programme will take up more of your time if your friend/relative receives additional therapy sessions that you may have to support them with.

We hope that the results from the study will help doctors, therapists, patients and their carers in the future when making decisions about treatment.

What are the alternatives for treatment?

Patients recovering from hip fracture repair will get standard NHS treatment, though this may vary in different areas. In this study every patient will get standard NHS treatment even if they are in the enhanced rehabilitation group. Currently, there are no other alternative treatment programmes available.

Do I have to take part?

No, taking part is voluntary. It is up to you to decide whether or not you want to take part. If you choose to take part you can also choose to stop at any time without giving a reason. This will not affect the care received by your friend/relative or how you are treated.

What happens if I change my mind?

If at any point you decide to stop taking part in the study we will not collect any further information from you. We will use any study information collected up until the time you stop taking part.

Will my participation be kept confidential?

Yes. All information collected about you during the course of the study will be handled according to relevant ethical and legal requirements. Your personal information will be kept strictly confidential and will only be accessed by people working on the study, or working to ensure the study is being run correctly.

You will be given a study number, which will be used along with your initials to identify you on each paper form. Your full name and telephone number will be included on your consent form and a copy of this will be sent to the study team at the coordinating centre for the study, the Liverpool Clinical Trials Centre (LCTC.) There may be instances (depending on your local NHS,) when a copy of your contact details will need to be sent to other

locations or Universities within your local area to arrange your follow up visits. Only members of the FEMUR III team will be given access to your contact details, they will be held securely and destroyed after your final follow-up.We will ask for your telephone number so that we can contact you for the telephone interviews during the study. Your telephone number will not be used for anything else without your consent.

The University of Liverpool is the sponsor for this study based in the United Kingdom. The University of Liverpool along with Bangor University will be using information from you in order to undertake this study and act as the joint data controllers for this study. This means that both joint data controllers are responsible for looking after your information and using it properly. The University of Liverpool will keep identifiable information about you for a maximum of 25 years after the study has finished. Arrangements for confidential destruction will then be made.

Your rights to access, change or move your information are limited, as we need to manage your information in specific ways in order for the research to be reliable and accurate. If you withdraw from the study, we will keep the information about you that we have already obtained. To safeguard your rights, we will use the minimum personally-identifiable information possible.

You can find out more about how we use your information in the "How we use your information" section on the trial website Femur3study.co.uk

Your friend/relative's NHS hospital and/or community health team will collect information from you for this research study in accordance with our instructions.

The only people working on the studywho will have access to information that identifies you will be people who need to confirm your participation in the study, contact you after 4 months to ask you questions about taking part in this study or audit the data collection process. The people who analyse the information will not attempt to identify you or find out your name or contact details.

Your friend/relative's NHS hospital and/or community health team will keep identifiable information about you from this study for up to a maximum of 25 years after the study has finished.

Additional information about future research:

When you agree to take part in a research study, the information you give may be provided to researchers running other research studies in this organisation and in other organisations. These organisations may be universities, NHS organisations or companies involved in health and care research in this country or abroad. Your information will only be used by organisations and researchers to conduct research in accordance with the UK Policy Framework for Health and Social Care Research.

This information will not identify you and will not be combined with other information in a way that could identify you. The information will only be used for the purpose of health and care research, and cannot be used to contact you or to affect your care. It will not be used to make decisions about future services available to you, such as insurance.

What will happen to the results of the study?

It is intended that the results of the study will be presented at conferences and published in medical journals so that we can explain to the medical, nursing and therapies' community what our research results have shown. Confidentiality will be ensured at all times and you will not be identified in any publication.

What if there is a problem?

If you have a concern about any aspect of this study, you should ask to speak with one of the research team who will do their best to answer your questions.

If at any time during the study you feel distressed or anxious please speak to your research team, a therapist or contact your GP.

If you wish to make a formal complaint, you can do this through the NHS Complaints Procedure. Details can be obtained from the hospital.

Every care will be taken in the course of this clinical study. However, in the unlikely event that you are harmed by taking part in this research project, compensation may be available and you may have to pay your related legal costs. The University of Liverpool holds insurance against claims from participants for

harm caused by their participation in this clinical trial. Participants may be able to claim compensation if they can prove that the University of Liverpool has been negligent. However, if you are harmed and this is due to someone's negligence in the NHS, then you may have grounds for a legal action for compensation against the NHS, but you may have to pay for your legal costs. The normal NHS complaints procedures should be available to you. The study sponsor accepts no liability for negligence on part of your NHS organisation's employees.

Additional information

The University of Liverpool is responsible for managing this study; they have asked that the day to day running of the study is carried out by the Liverpool Clinical Trial Centre (LCTC)part of the University of Liverpool. Additional support is provided by other researchers from participating universities (the study team).

This study is funded by the National Institute for Health Research's Health Technology Assessment programme (ref: 16/167/09).

The study has been reviewed by the National Institute for Health Research (NIHR), Health Research Authority and the National Research Ethics Service Committee. Tyne and Wear South reviewed the study and given approval for it to take place.

The views expressed are those of the author(s) and not necessarily those of the NHS, the NIHR or the Department of Health.

Thank you for reading this information sheet.

Contacts for further information

If you would like more information or have any questions about the FEMuR III study please talk to:

Principal Investigator: <PI name to go here>

Research Nurse: <RN name to go here>

Telephone: <Hospital contact number to go here>

Or visit the website: Femur3study.co.uk

If you wish to discuss the study with someone independent of the research team you can contact the local NHS Patient Advice and Liaison Service (PALS) or local equivalent on: <<Local PALS or equivalent telephone number to go here>>

.

Fracture in the Elderly Multidisciplinary Rehabilitation III

<<Local NHS Logo to go here>>

<Trust/Site address 1>
<Trust/Site address 2>
<Trust/Site address 3>
<postcode>
Tel: <telephone number>

Carer Participant Consent Form

To be completed by the Researcher:			
Site Name:			
Carer Study Number		Carer Initials:	
Participant Study Number			

To be completed by the carer:

Once you have read and understood each statement please enter your initials in each box	Initial
Example: I confirm that I have read and understand the Participant Information Sheet.	JS
1. I have read and understood the information sheet for this study. I have had the opportunity to ask questions and have had these answered satisfactorily.	
2. I understand that participation is voluntary and that I am free to withdraw from the study at any time, without giving a reason, and without my care or legal rights being affected.	
3. I understand that my data will be retained for a maximum of 25 years at site and by the Liverpool Clinical Trials Centre (LCTC) part of the University of Liverpool and that they will be stored in a confidential manner.	
4. I give permission for a copy of my consent form which will include my name and telephone number to be sent to members of the FEMUR III team (where it will be kept in a secure location), to allow confirmation that my consent was given.	
5. I understand that any data collected during the study may be looked at by authorised individuals from the study team and those listed under "Additional information" (NHS organisation, sponsor and regulatory authorities). I give permission for these individuals to have access to my records.	
6. I agree to allow information or results arising from this study to be used in future healthcare and/or medical research in a pseudo-anonymised form.	
7. I agree to take part in the above study.	
**The statements below are OPTIONAL** ***(you can still participate even if you only agree to the statements above):***	
8. I agree that I may be contacted in the future in relation to this or other related studies.	
9. I agree to being contacted by a study researcher to conduct a qualitative interview and for the interview to be recorded.<br>Telephone number:<br>(if you agree to this statement provide your details below):	

Your full name (please print):			
Your signature:		Date:	

To be completed by the Researcher (after carer has completed the form):			
Researcher full name (please print):			
Researcher signature:		Date:	

FEMuR III Carer PISC V4.0 19/09/2019
IRAS Project ID: 246828

Page 6 of 6
