# Supplementary material for: Protocol for a definitive randomised controlled trial and economic evaluation of a community-based rehabilitation programme following hip fracture: fracture in the elderly multidisciplinary rehabilitation—phase III (FEMuR III)
Source: BMJ Open. 2020 Oct 16;10(10):e039791. doi: 10.1136/bmjopen-2020-039791 (PMC7569930; doi:10.1136/bmjopen-2020-039791)
Supplement: Author's manuscript [file bmjopen-2020-039791.draft_revisions.pdf]

BMJ Open

BMJ Open is committed to open peer review. As part of this commitment we make the peer review history of every article we publish publicly available.

When an article is published we post the peer reviewers' comments and the authors' responses online. We also post the versions of the paper that were used during peer review. These are the versions that the peer review comments apply to.

The versions of the paper that follow are the versions that were submitted during the peer review process. They are not the versions of record or the final published versions. They should not be cited or distributed as the published version of this manuscript.

BMJ Open is an open access journal and the full, final, typeset and author-corrected version of record of the manuscript is available on our site with no access controls, subscription charges or pay-per-view fees (http://bmjopen.bmj.com).

If you have any questions on BMJ Open's open peer review process please email info.bmjopen@bmj.com

BMJ Open

Protocol for a definitive randomised controlled trial and economic evaluation of a community-based rehabilitation programme following hip fracture: Fracture in the Elderly Multidisciplinary Rehabilitation - Phase III (FEMuR III) [ISRCTN28376407]

Journal:	*BMJ Open*
Manuscript ID	bmjopen-2020-039791
Article Type:	Protocol
Date Submitted by the Author:	25-Apr-2020

Complete List of Authors:	Williams, Nefyn; University of Liverpool Faculty of Health and Life Sciences, Primary Care and Mental Health<br>Dodd, Susanna; University of Liverpool Faculty of Health and Life Sciences, Liverpool Clinical Trials Centre<br>Hardwick, Ben; University of Liverpool Faculty of Health and Life Sciences, Liverpool Clinical Trials Centre<br>Clayton, Dannii; University of Liverpool Faculty of Health and Life Sciences, Liverpool Clinical Trials Centre<br>Edwards, Rhiannon; Bangor University College of Physical and Applied Sciences, Centre for Health Economics & Medicines Evaluation<br>Charles, Joanna; Bangor University College of Health and Behavioural Sciences, Centre for Health Economics & Medicines Evaluation<br>Logan, Phillipa; University of Nottingham Faculty of Medicine and Health Sciences, Community Health Sciences<br>Busse, Monica; Cardiff University Centre for Trials Research<br>Lewis, Ruth; Bangor University College of Health and Behavioural Sciences, North Wales Centre for Primary Care Research<br>Smith, Toby; University of East Anglia Faculty of Medicine and Health Sciences<br>Sackley, Catherine; University of Nottingham Faculty of Medicine and Health Sciences<br>Morrison, Val; Bangor University College of Health and Behavioural Sciences, School of Psychology<br>Lemmey, Andrew; Bangor University College of Health and Behavioural Sciences, School of Sports, Health and Exercise Science<br>Masterson-Algar, Patricia; Bangor University College of Health and Behavioural Sciences, School of Healthcare Sciences<br>Howard, Lola; University of Liverpool Faculty of Health and Life Sciences, Liverpool Clinical Trials Centre<br>Hennessy, Sophie; University of Liverpool Faculty of Health and Life Sciences, Liverpool Clinical Trials Centre<br>Soady, Claire; University of Liverpool Faculty of Health and Life Sciences, Liverpool Clinical Trials Centre<br>Ralph, Penelope; University of Liverpool Faculty of Health and Life Sciences, Department of Primary Care and Mental Health<br>Dobson, Susan; University of Liverpool Faculty of Health and Life Sciences, Primary Care and Mental Health

	Dorkenoo, Shanaz; Health and Care Research Wales, Involving People Network

Keywords:	REHABILITATION MEDICINE, Hip < ORTHOPAEDIC & TRAUMA SURGERY, Clinical trials < THERAPEUTICS, HEALTH ECONOMICS, QUALITATIVE RESEARCH

SCHOLARONE™
Manuscripts

I, the Submitting Author has the right to grant and does grant on behalf of all authors of the Work (as defined in the below author licence), an exclusive licence and/or a non-exclusive licence for contributions from authors who are: i) UK Crown employees; ii) where BMJ has agreed a CC-BY licence shall apply, and/or iii) in accordance with the terms applicable for US Federal Government officers or employees acting as part of their official duties; on a worldwide, perpetual, irrevocable, royalty-free basis to BMJ Publishing Group Ltd ("BMJ") its licensees and where the relevant Journal is co-owned by BMJ to the co-owners of the Journal, to publish the Work in this journal and any other BMJ products and to exploit all rights, as set out in our licence.

The Submitting Author accepts and understands that any supply made under these terms is made by BMJ to the Submitting Author unless you are acting as an employee on behalf of your employer or a postgraduate student of an affiliated institution which is paying any applicable article publishing charge ("APC") for Open Access articles. Where the Submitting Author wishes to make the Work available on an Open Access basis (and intends to pay the relevant APC), the terms of reuse of such Open Access shall be governed by a Creative Commons licence – details of these licences and which Creative Commons licence will apply to this Work are set out in our licence referred to above.

Other than as permitted in any relevant BMJ Author's Self Archiving Policies, I confirm this Work has not been accepted for publication elsewhere, is not being considered for publication elsewhere and does not duplicate material already published. I confirm all authors consent to publication of this Work and authorise the granting of this licence.

For peer review only

Protocol for a definitive randomised controlled trial and economic evaluation of a community-based rehabilitation programme following hip fracture: Fracture in the Elderly Multidisciplinary Rehabilitation - Phase III (FEMuR III) [ISRCTN28376407]

Nefyn H Williams[1]

Email: Nefyn.williams@liverpool.ac.uk

Susanna Dodd[1]

Email: shinds@liverpool.ac.uk

Ben Hardwick[1]

Email: ben121@liverpool.ac.uk

Dannii Clayton[1]

Email: claytond@liverpool.ac.uk

Rhiannon T Edwards[2]

Email: r.t.edwards@bangor.ac.uk

Joanna M Charles[2]

Email: j.charles@bangor.ac.uk

Pip Logan[3]

Email: Pip.Logan@nottingham.ac.uk

Monica Busse[4]

Email: BusseME@cardiff.ac.uk

Ruth Lewis[2]

Email: r.lewis@bangor.ac.uk

Toby Smith[5]

Email: toby.smith@uea.ac.uk

Catherine Sackley[3]

For peer review only

Email: catherine.sackley@nottingham.ac.uk

Val Morrison[2]

Email: v.morrison@bangor.ac.uk

Andrew B Lemmey[2]

Email: a.lemmey@bangor.ac.uk

Patricia Masterson Algar[2]

Email: p.m.algar@bangor.ac.uk

Lolade Howard[1]

Email: awoyalel@liverpool.ac.uk

Sophie Hennessy[1]

Email: sophie.hennessy@liverpool.ac.uk

Claire Soady[1]

Email: claire.soady@liverpool.ac.uk

Penelope Ralph[1]

Email: p.ralph@liverpool.ac.uk

Susan Dobson[1]

Email: sldobson@liverpool.ac.uk

Shanaz Dorkenoo[6]

Email: shanaz519@btinternet.com

[1]University of Liverpool, UK

[2]Bangor University, UK

[3]University of Nottingham, UK

[4]Cardiff University, UK

[5]University of East Anglia, UK

For peer review only

[6]Involving People, Cardiff, UK

Corresponding author: Professor Nefyn H Williams, Department of Health Services Research, University of Liverpool, 1st Floor, Block B, Waterhouse Building, 1-5 Brownlow Street, Liverpool, L69 3GL, UK.

Tel: +44 (0)151 795 5305 e-mail: nefyn.williams@liverpool.ac.uk

Word count (4,000)

For peer review only

For peer review only - http://bmjopen.bmj.com/site/about/guidelines.xhtml

Abstract (300 words)

Introduction

Proximal femoral (hip) fracture is common, serious and costly. Rehabilitation may improve functional recovery but evidence of effectiveness and cost-effectiveness is lacking. An enhanced rehabilitation intervention was previously developed and a feasibility study tested the methods used for this randomised controlled trial (RCT).

The objectives are to compare the effectiveness and cost-effectiveness of the enhanced rehabilitation programme following surgical repair of proximal femoral fracture in older people compared with usual care.

Methods and analysis

Protocol for phase III, parallel-group, two-armed, superiority, pragmatic RCT with 1:1 allocation ratio. Allocation sequence by minimisation programme with a built in random element. Secure web-based allocation concealment. The two treatments will be usual care (control) and usual care plus an enhanced rehabilitation programme (intervention). The enhanced rehabilitation will consist of a patient-held information workbook, goal-setting diary and up to six additional therapy sessions. Outcome assessment and statistical analysis will be performed blind; patient and carer participants will be unblinded. Outcomes will be measured at baseline, 17 and 52 weeks' follow-up. Primary outcome at 52 weeks will be the Nottingham Extended Activities of Daily Living scale. Secondary outcomes will measure anxiety and depression, health utility, cognitive status, hip pain intensity, falls self-efficacy, fear of falling, grip strength and physical function. Carer strain, anxiety and depression will be measured in carers. All safety events will be recorded by the researchers when they are made aware of them. Each serious adverse event will be assessed to determine whether it is related to the intervention and expected.

For peer review only

For peer review only - http://bmjopen.bmj.com/site/about/guidelines.xhtml

Concurrent economic evaluation will be a cost-utility analysis from a health service and personal social care perspective. An embedded process evaluation will determine the mechanisms and processes that explain the implementation and impacts of the enhanced rehabilitation programme.

Ethics

NHS research ethics approval reference 18/NE/0300.

Registration details

ISRCTN28376407 registered on 23/11/2018.

Article Summary

Strengths and limitations of this study

- Pragmatic phase III randomised controlled trial following phase I intervention development and phase II feasibility study.
- Concurrent economic evaluation with a health service and personal social care perspective.
- Embedded process evaluation to determine the mechanisms and processes that explain the implementation and impacts of the enhanced rehabilitation programme.
- Only patients with mental capacity to consent are eligible, therefore excluding a large number of potential participants lacking capacity.

For peer review only - http://bmjopen.bmj.com/site/about/guidelines.xhtml

Introduction

Proximal femoral fracture, more commonly referred to as hip fracture, is a common, major health problem in old age [1]. It is projected to increase further as the population ages [2,3]. Mortality is high [4,5], and of those who survive to one year, 29% fail to regain their level of functioning, in terms of restrictions of activities of daily living [6]; many lose their independence. This imposes a large cost burden on society, estimated to be approximately £2.3 billion a year in the United Kingdom [7]. The majority of costs are incurred in the community and social care setting in the 12 months following hospital discharge, which are almost four times higher than the costs of the acute hospital admission [8]. Frail individuals are at particular risk of secondary future proximal femoral fracture, resulting in worse morbidity and mortality outcomes [9].

The National Institute of Health and Clinical Excellence (NICE) have issued guidelines for the management of hip fracture [10]. This includes the provision of a co-ordinated multidisciplinary rehabilitation programme starting in hospital during post-operative recovery and continuing in the community following discharge [10]. Where possible such rehabilitation programmes should consider individual patient goals, facilitate a return to pre-fracture independence and provide patients and carers with written information to support the rehabilitation programme and long-term outcomes. The Hip Sprint audit reported that community rehabilitation services were inconsistent [11].

Rationale

There have been four relevant Cochrane systematic reviews with inconclusive results [12-15]. These have examined different types and intensities of in-patient rehabilitation [12],

For peer review only - http://bmjopen.bmj.com/site/about/guidelines.xhtml

mobilisation strategies [13], psychosocial functioning after hip fracture [14] and rehabilitation for those with dementia following hip fracture surgery [15]. Other systematic reviews have reported improved walking ability [16], strength and physical function [17], including those with mild to moderate dementia [18]. These systematic reviews concluded that whilst individual components of rehabilitation programmes may aid recovery after a hip fracture, there is insufficient evidence to demonstrate clinical effectiveness or cost-effectiveness of an overall care pathway, and that further research is required.

A previous study [19] completed the first two phases of the Medical Research Council (MRC) framework for complex interventions [20]. The first phase developed an enhanced rehabilitation intervention which, in addition to usual care, included a patient-held workbook, a goal-setting diary and up to six additional home-based therapy sessions [21]. The second phase of the study was a randomised feasibility study, which assessed the acceptability of the new rehabilitation programme and the feasibility of trial methods for a definitive phase III randomised controlled trial (RCT) [22, 23]. Although this feasibility study was underpowered to assess effectiveness, the intervention showed a medium sized improvement in the Nottingham Extended Activities of Daily Living scale compared with usual care (Cohen's d 0.63). A process evaluation described the implementation of the rehabilitation programme and informed how to enhance recruitment and improve the intervention [24].

Risk and Benefits

The enhanced rehabilitation programme demonstrated a potential improvement in activities of daily living in the feasibility study. Possible risks of rehabilitation interventions

For peer review only - http://bmjopen.bmj.com/site/about/guidelines.xhtml

would include injury or falling when performing therapeutic exercises, which must be weighed against the risk to health of sedentary behaviour.

Primary Objective

To determine the effectiveness of an enhanced rehabilitation programme following surgical repair of proximal femoral fracture in older people compared with usual care, in terms of the performance of activities of daily living at 52 weeks follow-up.

Secondary Objectives

1. To compare the cost-effectiveness of an enhanced rehabilitation programme following surgical repair of proximal femoral fracture in older people compared with usual care at 52 weeks follow-up.
2. To determine the effectiveness of an enhanced rehabilitation programme following surgical repair of proximal femoral fracture in older people compared with usual care, in terms of the performance of activities of daily living at 17 weeks follow-up.
3. To determine the effectiveness of an enhanced rehabilitation package following surgical repair of proximal femoral fracture in older people compared with usual care, in terms of anxiety and depression at 17 and 52 weeks follow-up.
4. To assess whether the enhanced rehabilitation intervention creates change in self-efficacy, hip pain, cognitive function, fear of falling and physical function as potential mediators for improving activities of daily living at 17 and 52 weeks follow-up.
5. To assess whether the enhanced rehabilitation intervention creates change in strain, anxiety and depression in carers at 17 and 52 weeks follow-up.

For peer review only - http://bmjopen.bmj.com/site/about/guidelines.xhtml

6. To determine the mechanisms and processes that explain the implementation and impacts of the enhanced rehabilitation programme, and whether there are adverse effects.

Methods and Analysis

Trial design

This is a pragmatic, multisite, parallel-group, two-armed, superiority randomised controlled trial (RCT) with 1:1 allocation ratio, and an internal pilot phase (Figure 1). Outcome assessment and statistical analysis will be blinded; patient and carer participants and clinicians will be unblinded. A concurrent economic evaluation will be a cost-utility analysis from a health service and personal social care perspective. An embedded process evaluation will examine the mechanisms and processes that explain the implementation and impacts of the enhanced rehabilitation programme.

Trial Setting and Selection of Sites / Clinicians

Patients will be recruited on orthopaedic, rehabilitation and community hospital wards, or after hospital discharge home. The intervention will be delivered in the community, following hospital discharge, by community teams receiving referrals from the acute hospital sites and their associated community hospitals.

Selection of Sites/Clinicians

Sites have been opened to recruitment in Nottingham, Norfolk, north Wales, south Wales and east Kent. Further sites are planned in west Kent, Derby and Cheshire plus others. The site trial teams comprise principal investigators, hospital and community NHS staff, research assistants and support staff from clinical research networks.

For peer review only - http://bmjopen.bmj.com/site/about/guidelines.xhtml

Trial Population

Inclusion Criteria

1. Age 60 years or older
2. Recent proximal femoral fracture
3. Surgical repair by replacement arthroplasty, hemi-arthroplasty or internal fixation
4. Living in their own home prior to hip fracture
5. Living and receiving rehabilitation from the NHS in the area covered by the trial sites

Exclusion Criteria

1. Living in residential or nursing homes prior to hip fracture
2. Participants unable to understand English or Welsh
3. Lacking mental capacity to give informed consent

Carer Participants

We will also recruit carer participants to evaluate carer strain, anxiety and depression. These are defined as a relative or friend providing help with activities of daily living or physical care, at least four days a week. Carer participants will provide informed consent, but will not receive any trial intervention, so will not undergo eligibility screening or randomisation.

Trial Treatment/interventions

We plan to compare an enhanced rehabilitation intervention with usual rehabilitation care.

Usual rehabilitation care

Usual care consists of a multi-disciplinary rehabilitation delivered by the acute hospital, community hospital and community services depending on patients' individual needs at different times during their recovery and on the availability and accessibility of services in

For peer review only - http://bmjopen.bmj.com/site/about/guidelines.xhtml
For peer review only

different areas. The multidisciplinary team delivering care and rehabilitation may include: orthopaedic surgeons, orthogeriatricians, nurses, physiotherapists, occupational therapists, dieticians, pharmacists, GPs and social workers. The settings for care include acute orthopaedic or orthogeriatric wards, rehabilitation units in community hospitals, rehabilitation beds in care homes, the patient's own home and care home settings, all delivered by a variety of community teams in both health and social care services. There will be no restrictions on concomitant medications or treatments.

Enhanced rehabilitation

The main aim of the intervention is to enhance usual rehabilitation by increasing patients' self-efficacy [25], and increasing the amount and quality of patients' practice of physical exercise and activities of daily living to improve functional outcomes at follow-up. Self-efficacy will be enhanced by means of a patient-held information workbook and diary. In addition to whatever community-based rehabilitation is provided as part of usual care, we will provide up to six additional therapy (physiotherapist, occupational therapist or assistant) sessions, once patients are discharged home. The therapists will tailor these extra sessions, so the total number of sessions used, the time scale for their delivery, and the sessions' content will vary between patients according to need.

The workbook will include:

- Information about what has happened to them, and what to expect from their recovery;
- Information about NHS, council and voluntary sector services including falls' prevention programmes;

For peer review only - http://bmjopen.bmj.com/site/about/guidelines.xhtml

- How to manage their recovery, set goals and monitor progress of their rehabilitation; reduce fear of falling.

In each site, therapists have been trained to deliver the enhanced rehabilitation programme according to protocol, whilst at the same time tailoring the content and frequency of sessions to patients' needs. Throughout the running of this trial, therapists will receive on-going support via e-mails, newsletters and refresher events.

For peer review only

Outcomes

Patient participants will complete outcome measures at baseline, 17 and 52 weeks administered by a research assistant blinded to participant allocation. Follow-up assessments will be completed within participants' homes (Tables 1 and 2). The primary outcome will be the difference in Nottingham Extended Activities of Daily Living (NEADL) scale [26,27] at 52 week follow-up, between the usual rehabilitation arm and the enhanced rehabilitation arm. At baseline, the patient will be asked to recall the four weeks prior to hip fracture and not four weeks prior to completing this questionnaire. Secondary outcomes will include the Hospital Anxiety and Depression Scale (HADS) [28], economic measures will be EuroQol EQ-5D-3L [29] and Client Service Receipt Inventory (CSRI) [30]. A reduced version of this will be used at baseline to reduce participant burden as they recover from hip fracture surgery. Potential mediators of outcome will include a Visual Analogue Scale (VAS) for hip pain intensity [31], Falls Efficacy Scale - International (FES-I) (self efficacy) [32,33], and Visual Analogue Score - Fear of Falling (VASFoF) [34].

The research assistant will assess patient participants' cognitive function at baseline, 17 and 52 weeks using the Abbreviated Mental Test Score (AMTS) [35]. The research assistant will

For peer review only - http://bmjopen.bmj.com/site/about/guidelines.xhtml

measure physical function at baseline, 17 and 52 weeks using the grip strength test [36-38], and using the Short Physical Performance Battery (SPPB) [39,40] at 17 and 52 weeks. Carer participants will complete the Caregiver Strain Index (CSI) [41] and the Hospital Anxiety and Depression Scale (HADS) [28] at baseline, 17 and 52 weeks.

Qualitative interviews will take place with patients and carers after 17 weeks. These will gather data on trial participation and intervention design (see process evaluation below).

Routinely collected demographic, clinical and recruitment data will include the numbers of patients who are eligible, willing to be randomised, withdraw after randomisation, complete outcome measurements, also reasons for non-completion, age, gender, hip fracture type, surgery type, co-morbid conditions, place of residence prior to admission and place of discharge.

Sample size calculation

The phase II feasibility study results [23] informed the sample size calculation. The adjusted mean difference in the primary outcome measure (NEADL) between the intervention and control group in the feasibility trial was 3.0. Work completed by Wu, et al [27] has suggested that the minimum clinically significant difference is 2.4; this has been used within the sample size calculation for this phase III RCT. A two-point score in the NEADL scale would equate to an improvement in function from being independent around the home to being able to use public transport or get in and out of a car. The adjusted mean difference between the groups in NEADL in the randomised feasibility study had a standard deviation (SD) of 5.8. In this multi-site phase III RCT, a more diverse sample would be expected, so a

For peer review only - http://bmjopen.bmj.com/site/about/guidelines.xhtml

larger SD would be expected. Parker et al. [42] used NEADL in a rehabilitation RCT and found a SD of 10. Based on ANCOVA with alpha of 5% and 90% power to detect a difference of 2.4 (SD = 10, R^2 of covariate = 0.52) 352 patient participants would be required to complete the trial over both treatment groups. When considering the 79% retention rate in the feasibility study [23], the trial would need to recruit 446 patient participants.

Recruitment and Randomisation

Screening and Consent – Patient Participants

Patients with proximal femoral fracture will be identified and screened for eligibility, including mental capacity, by clinical staff on orthopaedic or rehabilitation wards. If the patients are eligible, and interested in the trial, the trial team researchers would then recruit patients following the trial's informed consent process. Assessment of eligibility may occur over an extended period, if for example, the patient is experiencing temporary delirium post-surgery. If during this period, patients are transferred to rehabilitation wards, community hospitals, or discharged home, then assessment will continue in these alternative locations. These assessments will be recorded in a screening log, including any reasons for ineligibility.

Informed consent - carer participants

For the purpose of this RCT, carers are defined as either a relative or friend caring for a hip fracture patient, helping them with activities of daily living or physical care on at least four days a week. They will be identified and recruited following the trial's informed consent process.

Randomisation Procedures

For peer review only

For peer review only - http://bmjopen.bmj.com/site/about/guidelines.xhtml

Patient participants who provide informed consent will complete baseline outcome measurements prior to randomisation. Randomisation will take place no later than six weeks after hip fracture repair surgery. The randomisation will have an allocation ratio of 1:1 within each stratum and across the trial. Randomisation will use a minimisation programme with a built in random element utilising factors that will not be made known to individuals in charge of recruitment to minimise any potential for predicting allocation. Randomisation will be completed by secure web access to the remote randomisation site at the clinical trials unit. The therapists delivering the enhanced rehabilitation intervention will receive an automated email when a participant has been allocated to the intervention group.

Blinding

This is a pragmatic trial comparing two rehabilitation interventions. It will therefore not be possible to blind participants or their clinicians to treatment group allocation. The research assistants will collect outcome measurements blind to treatment allocation. They will not be informed to which group the patient participants have been allocated, and will not be present at any of the therapy sessions. Before any home visits for follow-up assessments, they will ask participants not to reveal their treatment allocation. After the final follow-up assessment, they will complete a perception of allocation form, in order to monitor the level of blinding achieved for these researchers. Data analysis will be performed blind to treatment allocation.

Internal pilot

For peer review only - http://bmjopen.bmj.com/site/about/guidelines.xhtml

An internal pilot assessed site recruitment and participant recruitment and retention rates for the six months after the first site was open to recruitment from September 2019 to February 2020.

Progression criteria

- Number of sites open: 7 or more (go); 5-6 (amend); 4 or fewer (stop)
- Open site recruitment rate per month: 2 or more (go); 1-2 (amend); <1 (stop)
- Retention rate: 69% or higher (go); 50-68% (amend); 49% or fewer (stop)

Statistical Analysis

Final analysis will take place once all participants have been followed-up for 52 weeks, and the database has been locked. Analyses will be by 'intention to treat' for the primary and secondary outcomes on all randomised participants, in the group to which they were allocated, and for whom the outcomes of interest have been observed or measured.

Baseline

Demographic and baseline characteristics will be summarised separately using descriptive statistics for each randomised group to allow clinical assessment of whether balance was achieved between randomised groups. No statistical testing of differences between groups will be performed.

Analysis of effectiveness

Primary and secondary outcomes at baseline, 17 weeks' and 52 weeks' follow-up will be summarised for each treatment group using descriptive statistics at each time point. If normally distributed, the difference between group means (with 95% confidence intervals)

For peer review only - http://bmjopen.bmj.com/site/about/guidelines.xhtml

will be reported from an analysis of covariance (ANCOVA) adjusted for baseline score and stratification factors.

Missing data and withdrawals

Predictors of missing data will be investigated using regression models (including type of surgery, age, living arrangements and co-morbidities) and any significant predictors will be considered for inclusion in the models. In addition, given the two assessment points at 17 and 52 weeks, we will carry out a sensitivity analysis using a joint modelling approach to check whether there is any difference in outcome (here the longitudinal outcome rather than the outcome at 17 weeks or 52 weeks alone) between the randomised arms adjusted for dropouts or missing values.

Instrumental variable regression

The impact of engagement with the intervention will be assessed using instrumental variable (IV) regression, using the number of face-to-face direct rehabilitation sessions over 52 weeks' follow up as a continuous measure of engagement. Additional exploratory IV regression analyses will use in turn: the total number of rehabilitation sessions (face-to-face plus telephone), total time (in minutes) spent in face-to-face direct rehabilitation sessions, and total time (in minutes) spent in all rehabilitation sessions (i.e. face-to-face and telephone). The suitability of using randomisation as the instrument in these IV regression models will be assessed using tests of exogeneity, redundancy and under/weak identification.

Mediation analyses

The hypothesised mechanism of change for the enhanced rehabilitation intervention is that participants' primary outcome (activities of daily living) is mediated by self-efficacy, hip pain,

For peer review only - http://bmjopen.bmj.com/site/about/guidelines.xhtml

cognitive function, fear of falling and physical function. If the enhanced rehabilitation intervention has a significant effect on primary outcome ($p<0.05$) for enhanced rehabilitation in ANCOVA, causal mediation analysis will be used to determine whether each of these potential mediators predict change in NEADL at 52 weeks. Initial assessments will determine whether the randomised intervention affects each putative mediator in turn. For those putative mediators that are significantly ($p<0.1$) affected by the randomised intervention, mediation analysis will be carried out adjusting for baseline covariates that predict both the mediator and NEADL, potentially including type of surgery, age, living arrangements (alone/with others) and co-morbidities. Sensitivity analyses will assess the potential impact of unmeasured confounding between the mediator and NEADL.

Economic analysis

The enhanced rehabilitation programme will be fully costed using unit costs from a public sector multiagency perspective. Unit costs will be obtained from national sources of reference costs [43,44] and applied to information received from pilot questionnaires, namely salary band of therapists, time spent with the patient conducting rehabilitation, costs of travel and costs of any additional equipment. Costs of health and social care services used by the participants will also be costed using national sources of reference costs. The costs of service use and the cost of the intervention will be added together for use in a cost-effectiveness analysis.

The EQ-5D (3L) will be used to calculate Quality Adjusted Life Years (QALYs) over the 52 week trial period, using the area under the curve method [45,46]. A cost-utility analysis will be conducted to calculate a cost per QALY of the enhanced rehabilitation intervention. This

For peer review only - http://bmjopen.bmj.com/site/about/guidelines.xhtml

cost per QALY generated will be compared to the NICE threshold range of £30,000 per QALY [47]. We will bootstrap differences in costs and outcomes (EQ-5D-3L) between the two groups, producing a 95% confidence interval around these differences.

Process Evaluation

The process evaluation will aim to identify and explain all mechanisms and processes (i.e. the intervention theory) that enabled or acted as a barrier to the implementation of the enhanced rehabilitation intervention. The process evaluation will help build a picture of how the intervention was carried out in reality, and what factors shaped it. By carrying out a process evaluation, it will be possible to identify if observed impacts are solely due to the enhanced rehabilitation programme, or if these impacts are a result of a number of external and internal variables that are closely linked to the environment and the context in which the intervention takes place [48-51].

The specific objectives will be to:

- Refine the programme theory from the previous realist review that was used to develop the intervention [21]. This programme theory will explain how the researchers envisage the intervention to work, to reach its expected outcomes.
- Investigate therapists' expectations and experience of implementation, their previous experience and training, and their learning throughout the conduct of the trial.
- Investigate the mechanisms driving and shaping the tailoring of the enhanced rehabilitation intervention to individual patients.
- Investigate trial participants' (patients and carers) experiences and views about their involvement in the trial, as well as their experience of care in either arm of the trial.

For peer review only - http://bmjopen.bmj.com/site/about/guidelines.xhtml

- Map and synthesise all data collected in order to test the refined programme theory and explain the trial findings.

Process evaluation data collection

Semi-structured telephone interviews will be conducted with:

- A purposive sample of 60 patient participants in each of the two trial arms and up to 30 of their carers. Patients will be purposively sampled to ensure diversity based on age, functional impairment (using baseline NEADL scores) and the presence or absence of a family carer. Interviews will take place after the 17-week assessment and will be audio recorded.
- The therapists delivering the enhanced rehabilitation programme, which will explore implementation from the therapists' perspectives. Interviews will be conducted midway through their involvement in the trial, and at the end, in order to investigate learning over time.

Data on intervention delivery and adherence:

- Therapists will record key reflections on 'critical incident reports'.
- The visiting therapist will record the length and content of each extra rehabilitation therapy session on a case report form.
- All patient participants will be given a therapy session record, where visiting therapists will record the number, length and content of usual rehabilitation care. Whenever possible, routinely collected electronic data that therapists complete on their Therapy Manager system, or its equivalent, will be collected.
- An online questionnaire will be emailed to participating therapists in order to capture therapists' relevant training, previous experience and familiarity with the trial intervention.

For peer review only - http://bmjopen.bmj.com/site/about/guidelines.xhtml

Qualitative data will be analysed following a thematic analysis approach [52] that will be guided by the proposed programme theory. Quantitative data (record forms and online questionnaires) will be analysed using descriptive statistics, which will allow the exploration of frequency of responses. All data sets will be synthesised in order to describe the complex nature of the enhanced rehabilitation intervention.

Patient and Public Involvement

There has been patient and public involvement (PPI) at all stages including refining the research question, choosing outcomes relevant to patients, commenting on the burden of the intervention and of trial participation. A PPI co-investigator will continue to contribute to the trial management group, including comments on patient facing materials and the dissemination plan.

Ethics and Dissemination

NHS research ethics approval was obtained from North East – Tyne & Wear South Research Ethics Committee, reference 18/NE/0300. The current protocol is version 4.0 (11/12/20019). A Trial Steering Committee is providing overall supervision and an Independent Data Safety and Monitoring Committee is responsible for reviewing and assessing recruitment, interim monitoring of safety and effectiveness, trial conduct and external data.

All safety events will be recorded by researchers when they are made aware of the event by the patient, carer, the treating clinicians, or therapists. Adverse event reports and serious adverse events (SAEs) not related to the intervention will be entered on to the remote data entry system. Each SAE will be assessed by the relevant PI to determine whether it is related

For peer review only - http://bmjopen.bmj.com/site/about/guidelines.xhtml

For peer review only

to the intervention. A related SAE will be assessed by the CI to determine whether it is expected. If the SAE is related and unexpected (RUSAE) it will be reported to the Research Ethics Committee (REC) and sponsor in an expedited manner.

Reporting of the trial will be consistent with the CONSORT 2010 Statement (patient reported outcomes and non-pharmcological interventions) [53]. We will submit the final report to a peer-reviewed academic journal, according to our publication strategy and authorship policy. Research data will be available for secondary analysis upon reasonable request.

Trial Status

At the time of submission this trial had been open in nine sites and had recruited 96 patients and 10 carers, with a recruitment rate of two patient participants per site per month and a retention rate of 83%, which fulfilled the progression criteria of the internal pilot. However, recruitment to the trial is currently suspended because of the COVID-19 pandemic. Wherever possible, participants already recruited into the trial will complete their follow-up assessments over the telephone or by post, extra rehabilitation sessions will be delivered over the telephone. When trial recruitment resumes, updated recruitment information will be found on the website http://femur3study.co.uk/

Abbreviations

AMTS	Abbreviated Mental Test Score
ANCOVA	Analysis of Covariance
CI	Chief Investigator

For peer review only - http://bmjopen.bmj.com/site/about/guidelines.xhtml

CSI	Carer Strain Index
CSRI	Client Service Receipt Inventory
EQ-5D-3L	EuroQol 5 Dimensions 3 Levels
FEMuR	Fracture in the Elderly Multidisciplinary Rehabilitation
FES-I	Falls Efficacy Scale - International
GP	General Practitioner
HADS	Hospital Anxiety and Depression Scale
ISRCTN	International Standard Randomised Controlled Trial Number
IV	Instrumental Variable
LCTC	Liverpool Clinical Trials Centre
NEADL	Nottingham Extended Activities of Daily Living
NHS	National Health Service
NICE	National Institute of Health and Clinical Excellence
PI	Principal Investigator
PPI	Patient and Public Involvement
QALY	Quality Adjusted Life Year
RCT	Randomised Controlled Trial
REC	Research Ethics Committee
RUSAE	Related Unexpected Serious Adverse Event
SAE	Serious Adverse Event
SPPB	Short Physical Performance Battery
VAS	Visual Analogue Scale
VASFoF	Visual Analogue Score - Fear of Falling

For peer review only

For peer review only - http://bmjopen.bmj.com/site/about/guidelines.xhtml

References

1. Royal College of Physicians, *National Hip Fracture Database annual report*. 2016: London.
2. Baker PN, Salar O, Ollivere BJ, Forward DP, Weerasuriya N, Moppett IK, et al., Evolution of the hip fracture population: time to consider the future? A retrospective observational analysis. *BMJ Open,* 2014. 4(4): p. e004405.
3. White SM and Griffiths R, Projected incidence of proximal femoral fracture in England: a report from the NHS Hip Fracture Anaesthesia Network (HIPFAN). *Injury,* 2011. 42(11): p. 1230-3.
4. Roche JJ, Wenn RT, Sahota O, and Moran CG, Effect of comorbidities and postoperative complications on mortality after hip fracture in elderly people: prospective observational cohort trial. *BMJ,* 2005. 331(7529): p. 1374.
5. Schnell S, Friedman SM, Mendelson DA, Bingham KW, and Kates SL, The 1-year mortality of patients treated in a hip fracture program for elders. *Geriatr Orthop Surg Rehabil,* 2010. 1(1): p. 6-14.
6. Bertram M, Norman R, Kemp L, and Vos T, Review of the long-term disability associated with hip fractures. *Injury Prev.,* 2011. 17(6): p. 365-70.
7. *National Osteoporosis Society. Key facts and figures. Accessed September 2014. Available at http://www.nos.org.uk/page.aspx?pid=328.*
8. Tian Y, Thompson J, Buck D, Sonola L. Exploring the system-wide costs of falls in older people in Torbay. London: The King's Fund. 2013.
9. Schnell S, Friedman SM, Mendelson DA, et al. The 1-year mortality of patients treated in a hip fracture program for elders. Geriatr Orthop Surg Rehabil 2010;1:6–14.

For peer review only - http://bmjopen.bmj.com/site/about/guidelines.xhtml

10. National Clinical Guideline Centre. Hip fracture: the management of hip fracture in adults| Guidance and guidelines | NICE. 2011 http://www. nice. org. uk/ guidance/ cg124 (accessed 2020).

11. Royal College of Physicians' Falls and Fragility Fracture Audit Programme. Recovering after a hip fracture: helping people understand physiotherapy in the NHS. Physiotherapy 'Hip Sprint' audit report 2017. https://www.fffap.org.uk/FFFAP/landing.nsf/PHFSA201802w1.pdf (accessed 2020).

12. Handoll HHG, Cameron ID, Mak JCS, et al. Multidisciplinary rehabilitation for older people with hip fractures. Cochrane Database Syst Rev 2009;15.

13. Handoll HH, Sherrington C, Mak JC. Interventions for improving mobility after hip fracture surgery in adults. Cochrane Database Syst Rev 2011:CD001704.

14. Crotty M, Unroe K, Cameron ID, et al. Rehabilitation interventions for improving physical and psychosocial functioning after hip fracture in older people. Cochrane Database Syst Rev 2010:CD007624.

15. Smith TO, Smith TO, Gilbert AW, Sreekanta A, Sahota O, Griffin XL, Cross JL, Fox C, Lamb SE. Enhanced rehabilitation and care models for adults with dementia following hip fracture surgery. Cochrane Database Syst Rev 2020;(2): CD010569

16. Chudyk A, Jutai J, Petrella R, Speechley M. Systematic review of hip fracture rehabilitation practices in the elderly. Archives of Physical Medicine and Rehabilitation 2009;90:246-62.

17. Auais MA, Eilayyan O, Mayo NE. Extended Exercise Rehabilitation After Hip Fracture Improves Patients' Physical Function: A Systematic Review and Meta-Analysis. Physical Therapy 2012;92:1437-51.

For peer review only - http://bmjopen.bmj.com/site/about/guidelines.xhtml

18. Allen J, Koziak A, Buddingh S, Liang J, Buckingham J, Beaupre L. Rehabilitation in patients with dementia following hip fracture: A systematic review. Physiotherapy Canada 2012;64:190-201.

19. Williams NH, Roberts JL, Din NU, et al. Developing a multidisciplinary rehabilitation package following hip fracture and testing in a randomised feasibility study: Fracture in the Elderly Multidisciplinary Rehabilitation (FEMuR). Health Technol Assess 2017;21:1–528.

20. Medical Research Council. Developing and evaluating complex interventions: new guidance. London: Medical Research Council, 2008.

21. Roberts JL, Din NU, Williams M, et al. Development of an evidence-based complex intervention for community rehabilitation of patients with hip fracture using realist review, survey and focus groups. BMJ Open 2017;7:e014362.

22. Williams NH, Hawkes C, Din NU, et al. Fracture in the Elderly Multidisciplinary Rehabilitation (FEMuR): study protocol for a phase II randomised feasibility study of a multidisciplinary rehabilitation package following hip fracture [ISRCTN22464643]. Pilot Feasibility Stud 2015;1:13.

23. Williams NH, Roberts JL, Din NU, et al. Fracture in the Elderly Multidisciplinary Rehabilitation (FEMuR): a phase II randomised feasibility study of a multidisciplinary rehabilitation package following hip fracture. BMJ Open 2016;6:e012422.

24. Roberts JL, Pritchard AW, Williams M, Totton N, Morrison V, Din NU, Williams NH. Mixed methods process evaluation of an enhanced rehabilitation intervention for elderly hip fracture patients. BMJ Open 2018;8:e021486.

25. Bandura A. Self-efficacy: Toward a unifying theory of behavioral change. *Psychol Rev* 1977; 84: 191–215.

For peer review only - http://bmjopen.bmj.com/site/about/guidelines.xhtml

For peer review only

26. Nouri F, Lincoln N. An extended activities of daily living scale for stroke patients. Clin Rehabil. 1987;1:301–5.

27. Wu CY, Chuang LL, Lin KC, et al. Responsiveness, minimal detectable change, and minimal clinically important difference of the Nottingham extended activities of daily living scale in patients with improved performance after stroke rehabilitation. Arch Phys Med Rehabil 2011;92:1281–7.

28. Zigmond A, Snaith R. The Hospital Anxiety and Depression Scale. Acta Psychiatr Scand. 983;67:1983.

29. EuroQol Group. EuroQol – a new facility for the measurements of health-related quality of life. Health Policy 1990;16:199-208.

30. Database of Instruments for Resource Use Measurement. Client Service Receipt Inventory (CSRI). http://www.dirum.org/instruments/details/44. Accessed 31 January 2019.

31. Brokelman RH, van Loon C, Hol A, van Kampen A, Veth R. The validation of the visual analogue scale for patient satisfaction after total hip arthroplasty. Eur Orthop Traumatol 2012;3:101–5.

32. Hauer K, Yardley L, Beyer N, Kempen G, Dias N, Campbell M, et al. Validation of the Falls Efficacy Scale and Falls Efficacy Scale International in geriatric patients with and without cognitive impairment: results of self-report and interview-based questionnaires. Gerontology. 2010;56:190–9.

33. Yardley L, Beyer N, Hauer K, Kempen G, Piot-Ziegler C, Todd C. Development and initial validation of the Falls Efficacy Scale-International (FES-I). Age Ageing. 2005;34:614–9.

For peer review only

34. Scheffer AC, Schuurmans MJ, van Dijk N, van der Hooft T, de Rooij SE. Reliability and validity of the visual analogue scale for fear of falling in older persons. J Am Geriatr Soc. 2010;58:2228–30.

35. Hodkinson H. Evaluation of a mental test score for assessment of mental impairment in the elderly. Age Aging. 1972;1:233–8.

36. Giampaoli S, Ferrucci L, Cecchi F, Lo Noce C, Poce A, Dima F, et al. Hand-grip strength predicts incident disability in non-disabled older men. Age Ageing.1999;28:283–8.

37. Rantanen et al., 'Midlife hand grip strength as a predictor of old age disability' JAMA 1999 281(6):558-560;

38. Sallinen et al., 'Hand-grip strength cut-points to screen older persons at risk for mobility limitation' 2010 J Am Geriatr Soc 58(9):1721-1726.

39. Guralnik JM, Simonsick EM, Ferrucci L, Glynn RL, Berkman LF, Blazer DG, et al. A short physical performance battery assessing lower extremity function: association with self-reported disability and prediction of mortality nursing home admission. J Gerentol 1994; 49(2):M85-M94.

40. Guralnik JM, Ferrucci L, Pieper CF, Leveille SG, Markides KS, Ostir GV, et al. Lower extremity function and subsequent disbility: consistency across studies, predictive modes, and value of gait speed alone compared with the short physical performance battery. J Gerentol A 2000; 55(4):M221-M231.

41. Robinson BC. Validation of a caregiver strain index. J Gerontol. 1983;38(3):344–8.

42. Parker S G, Oliver P, Pennington M, Bond J, Jagger C. Rehabilitation of older patients: day hospital compared to rehabilitation at home. A randomised controlled trial. Health Technol Assess 2009;13(39).

For peer review only

43. Department of Health. NHS reference costs 2015 to 2016. Accessed April 2020. https://www.gov.uk/government/publications/nhs-reference-costs-2015-to-2016

44. Curtis L, Burns A. Unit costs of health and social care 2019. Personal Social Services Research Unit (PSSRU). Accessed April 2020. https://www.pssru.ac.uk/project-pages/unit-costs/unit-costs-2019/

45. Glick H, Doshi J, Sonnad S, Polsky D. Economic evaluation in clinical trials. Oxford: Oxford University Press; 2007.

46. Drummond MF, Sculpher MJ, Torrance GW, O'Brien BJ, Stoddart GL. Methods for the economic evaluation of health care programmes. Oxford: Oxford University Press; 2005.

47. National Institute for Health and Care Excellence. Guide to the methods of technology appraisal. Accessed on 11 June 2014. http://www.nice.org.uk/article/pmg9/resources/non-guidance-guide-to-themethods-of-technology-appraisal-2013-pdf. 2013.

48. Oakley, A., Strange V., Bonell C., Allen E. & Stephenson J. (2006). Process evaluation in randomised controlled trials of complex interventions. Health Services Research 2006; 332: 413-416.

49. Carroll C, Patterson M, Wood S, Booth A, Rick J, Balain S. A conceptual framework for implementation fidelity. Implementation Science 2007; 2: 40-49.

50. Grant A, Treweek S, Dreischulte T, Foy R, Guthrie B. Process evaluations for cluster-randomised trials of complex interventions: a proposed framework for design and reporting. Trials 2013; 14: 15-25.

51. Masterson-Algar P, Burton CR, Rycroft-Malone J, Sackley C, Walker M. Towards a programme theory for fidelity in the evaluation of complex interventions. Journal of Evaluation of Clinical Practice 2014; 20(4): 445-452.

For peer review only - http://bmjopen.bmj.com/site/about/guidelines.xhtml

52. Braun V, Clarke V. Using thematic analysis in psychology. Qualitative Research in Psychology 2006; 3: 77-101.

53. Schulz KF, Altman DG , Moher D , et al. CONSORT 2010 statement: updated guidelines for reporting parallel group randomised trials. BMC Med 2010;8:18

Author Statement

NHW was the chief investigator and grant holder, was responsible for study design, conduct and analysis and had overall responsibility for the study and acts as guarantor. LH and SH were the trial co-ordinators and BH was the senior trial co-ordinator overseeing day-to-day conduct, and provided methodological input. LH was the initial trial co-ordinator and contributed to writing the trial protocol and setting up the trial. SD wrote the statistical analysis plan. RhTE and JC wrote the health economic analysis plan. PMA wrote the process evaluation analysis plan with NHW. RL is a lead applicant from Bangor University and oversees sites in North Wales. MB is a lead applicant from Cardiff University and oversees sites in South Wales. PL is a lead applicant from University of Nottingham and oversees sites in Nottingham. CS is a lead applicant from Kings College London and oversees sites in Kent. TS is a lead applicant from the University of East Anglia and oversees the site in East Anglia. VM and ABL were co-investigators responsible for study design, methodological oversight and provided health psychology and exercise science expertise respectively. All authors were involved in drafting, revising and approving this manuscript.

Acknowledgements

For peer review only - http://bmjopen.bmj.com/site/about/guidelines.xhtml

The authors would like to thank all of the principal investigators, research nurses, hospital staff, community rehabilitation teams, physiotherapists, occupational therapists and their assistants who are providing the additional therapy sessions. In Nottingham: Miriam Golding-Day, Katie Robinson, Jane Horne, Frances Allen. In north Wales: Matthew Jones, Lauren Porter, Jane Stockport, Mary Roberts, Victoria Whitehead, Yogesh Joshi, Gareth Evans, Cathy Baker, Jeannie Bishop, Jane Heron, Llinos Davies. In south Wales: Rachel Lowe, Rebecca Milton, Andrew Dean Young, Peter Lewis, Lisa Roche. In East Anglia: Stephanie Tuck, Catherine Mingay, Stephanie Howard. In east Kent: Danielle Pearce, Joanne Jackson, Sarah Chapman, Andrew Smith, Alex Chipperfield, Joanne Deery.

Special thanks to the LCTC data management team Clare Jackson and Dianne Wheatley, LCTC information systems team Keith Kennedy and Janet Harrison, trial co-ordinator assistant Kieran Crabtree and quality assurance manager Katie Neville. Research Assistants/Research Project Support Officers who perform follow-up visits. Others who have contributed to the trial management group Kevin Anthony, Gail Arnold, Janine Bates, Suzannah Evans, Mark Howells, Lara Lavelle-Langham, Nic Nikolic, Jessica Roberts. Also the North West Coast Clinical research Network Lisa Cheng, Julia Murgarza.

We very much appreciate the work of the Trial Steering Committee Gail Mountain (chair), Graeme Holt, Helen Hughes, George Kernohan, Tosan Okoro, Nikki Totton, and the Independent Data and Safety Monitoring Committee Chris Robertson (chair), Diane Dixon, Peter Giannoudis, Barbara Hanratty.

Funding

For peer review only

For peer review only - http://bmjopen.bmj.com/site/about/guidelines.xhtml

This work was supported by the National Institute for Health Research's Health Technology Assessment Programme, grant number 16/167/09. The views and opinions expressed therein are those of the authors and do not necessarily reflect those of the HTA, NIHR, NHS or the Department of Health.

Trial Sponsor

University of Liverpool: sponsor's reference: UoL001378. Contact: Mr Alex Astor, University of Liverpool, 2nd Floor Block D Waterhouse Building, 3 Brownlow Street, Liverpool L69 3GL. The university has appropriate clinical trials and professional indemnity insurance.

Conflicts of Interest

NHW reports additional grants from NIHR HS&DR outside the submitted work and membership of the NIHR HTA programme funding committee (commissioned research).

Data Statement

Technical appendix, statistical code, and dataset will be made available.

For peer review only - http://bmjopen.bmj.com/site/about/guidelines.xhtml

Table 1 Outcome measures

**Patient Completed Measures - Primary**	**Description**	**Range**
Nottingham Extended Activities of Daily Living (NEADL) scale [26, 27]	Activities of daily living (mobility, kitchen, domestic, leisure) with higher score indicating greater independence	(0-66)
**Patient Completed Measures - Secondary**		
Hospital Anxiety and Depression Scale (HADS) [28]	Anxiety and depression in patients with physical health problems. Two sub-scales (0-21) with higher score indicates greater anxiety or depression	(0-21)
**Patient Completed Economic Measures**		
EuroQol EQ-5D-3L [29]	Health utility index with five dimensions (mobility, self-care, usual activities, pain/discomfort, anxiety/depression) and three levels to give health states converted to a utility weight. Also Visual Analogue Score (VAS) for health state today	Health utility weight from 0 (death) to 1.0 (perfect health) also with negative values VAS (0-100)
Client Service Receipt Inventory (CSRI) [30]	Use of health and social care services	According to activity
**Patient Completed Process Measures (potential mediators of outcomes)**		
Visual Analogue Scale (VAS) for hip pain intensity [31]	VAS of current hip pain intensity	(0-10cm)
Falls Efficacy Scale - International (FES-I) (self-efficacy) [32,33]	How concerned a patient is about falling when performing 16 activities of daily living both inside and outside of the home, rated from 1 (not at all concerned) to 4 (very concerned)	(16-64)
Visual Analogue Score - Fear of Falling (VASFoF) [34]	VAS with higher scores indicating greater fear of falling	(0-10cm)
**Assessment of cognitive function**		

For peer review only - http://bmjopen.bmj.com/site/about/guidelines.xhtml

Abbreviated Mental Test Score (AMTS) [35, 36]	Detecting and monitoring cognitive impairment. 10 items with lower scores indicating worse cognitive function	(0-10)
**Objective measures of physical function**		
Grip strength [37]	Hand dynamometer	According to meter reading
Short Physical Performance Battery (SPPB) [40,41]	Physical function tests assessing lower limb function in terms of balance, gait, strength and endurance. Higher score indicates greater function	(0-12)
**Carer completed measure - secondary outcome**		
Caregiver Strain Index (CSI) [42]	13-items in the domains: employment, financial, physical, social and time. Positive responses to seven or more items indicate a greater level of strain	(0-13)
Hospital Anxiety and Depression Scale (HADS) [28]	Anxiety and depression in carers. Two sub-scales (0-21) with higher score indicates greater anxiety or depression	(0-21)

For peer review only

Table 2 FEMuR III protocol schedule of forms and procedures

Participant follow-up visits should take place at 17 (+/- 2 weeks) and 52 (+/- 2 weeks) weeks post randomisation.

Procedures	Screening	Baseline / Randomisati	Trial intervention	17 weeks post randomisati	Qualitative interviews	52weeks post randomisati
**Eligibility screening and consent**						
Assessment of eligibility criteria	X					
Written and informed consent (patient / carer))	X					
Confirm consent		X	X	X	X	X
Randomisation		X				
Discharge data		X				
**Outcome measurement - patient**						
NEADL		X		X		X
HADS		X		X		X
AMTS		X		X		X
VAS hip pain intensity		X		X		X
FES-I		X		X		X
VASFoF		X		X		X
EQ-5D-3L		X		X		X
CSRI		X		X		X
Grip strength		X		X		X
SPPB				X		X
**Outcome measurement - carer**						
CSI		X		X		X
HADS		X		X		X
**Trial Intervention****			X			
**Qualitative interviews**						
Re-affirm consent verbally specifically for qualitative phone interview. (patient / carer)					X	

Procedures	Screening	Baseline / Randomisati	Trial intervention	17 weeks post randomisati	Qualitative interviews	52weeks post randomisati
Qualitative telephone interview					X	
**Safety Event Reporting**						
Monitoring of Adverse Events			X	X	X	X
Monitoring of Serious Adverse Events			X	X	X	X

* Randomisation and baseline should take place no later than 6 weeks after hip fracture repair surgery

** If randomised to intervention arm.

For peer review only

For peer review only - http://bmjopen.bmj.com/site/about/guidelines.xhtml

Figure Legend

Figure 1 **Participant Flowchart for FEMuR III**

For peer review only - http://bmjopen.bmj.com/site/about/guidelines.xhtml

Figure 1 Participant Flow Chart for FEMuR III

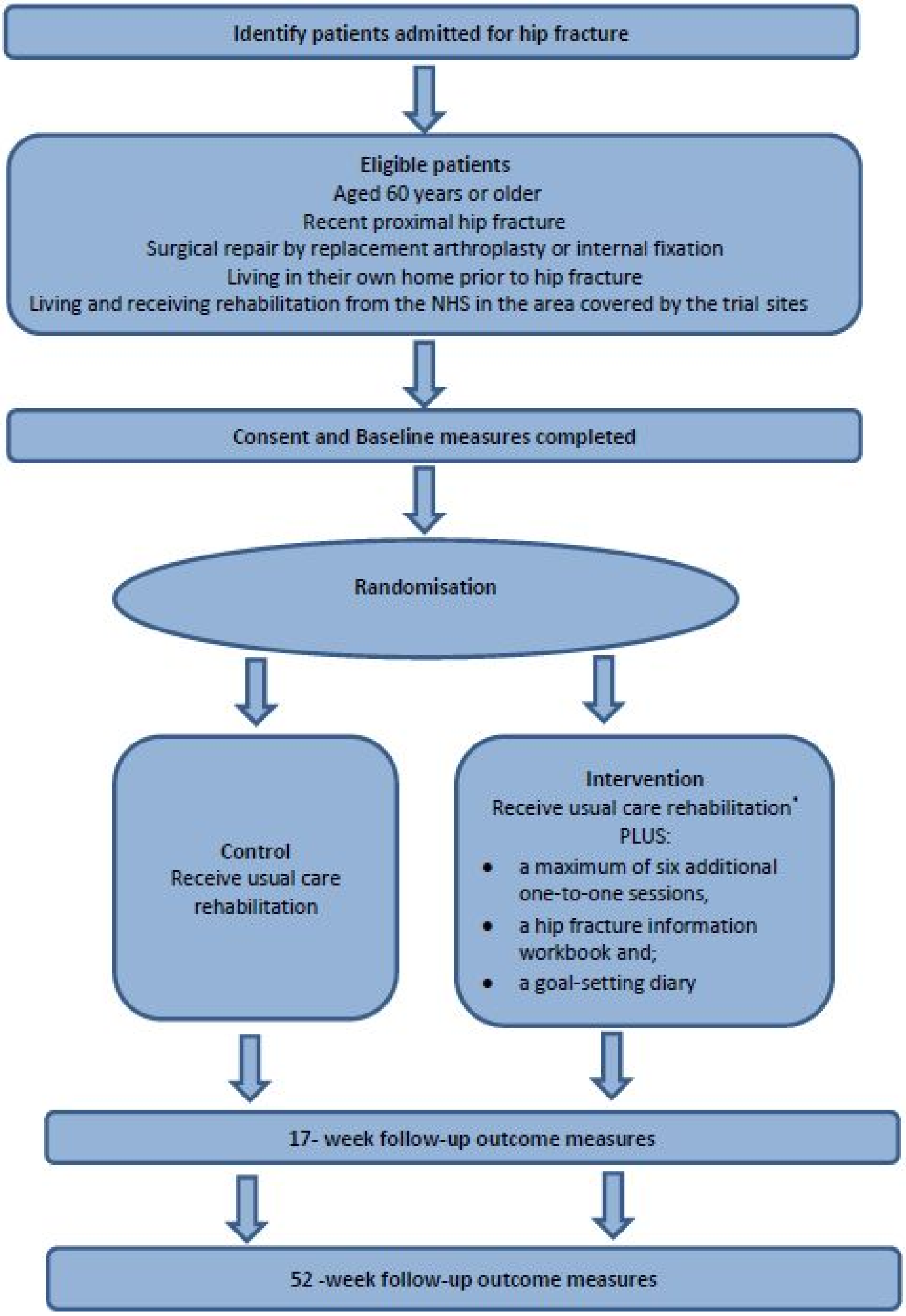

Appendix 1 FEMuR III Patient Participant Information Sheet and Informed Consent Forms

Fracture in the Elderly Multidisciplinary Rehabilitation III

<<Local NHS Logo to go here>>

<Trust/Site address 1>
<Trust/Site address 2>
<Trust/Site address 3>
<postcode>
Tel: <telephone number>

Patient Participant Information Sheet

Contents	Page
You are invited to take part in FEMuR III	1
Why are we doing the FEMuR III study?	2
What is the Enhanced Rehabilitation Package?	2
Why have I been asked to take part?	2
What will I have to do if I take part?	2
Timeline of visits	3
How will I know which treatment I'm going to have?	3
What are the benefits and risks of taking part?	3
What are the alternatives for treatment?	3
Do I have to take part?	4
What happens if I change my mind?	4
Will my participation be kept confidential?	4
What will happen to the results of the study?	5
What if there is a problem?	5
Additional information	5
Additional information about future research	5
Contacts for further information	6
Consent Form	7

Important Contact Information

Thank you for taking the time to read this information sheet. We hope you will find this information helpful.

If you would like a large print version of this information sheet please ask your research team.

If you have any questions about this study please talk to your research team:

<Add contact details for PI/RN i.e., name and telephone number>

Website: Femur3study.co.uk

You are invited to take part in FEMuR III

Important things to know about FEMUR III:

- FEMuR III aims to compare a new enhanced rehabilitation package with standard NHS care for patients who have had surgery to repair a hip fracture.
- We are interested in the recovery of patients aged over 60 years old who lived independently before they suffered a hip fracture even if they were in receipt of personal care at home.
- Being part of the study means you will receive either standard care or standard care **plus** enhanced rehabilitation when you leave hospital.
- Standard care can vary but usually involves community-based physiotherapy. The enhanced rehabilitation package will provide additional physiotherapy, occupational therapy and some 'self-help' tools to aid recovery.
- You have been given this information sheet as you might be eligible to take part in this study.
- Before you decide whether or not to take part it is important for you to understand why the research is being done and what it will involve for you. Please take time to read the following information carefully and discuss it with friends or relatives if you wish.
- Taking part is voluntary. If you don't want to take part then you don't need to give a reason.
- If you have someone who provides you with help for most days of the week with activities of daily living or physical care, we would also like them to be involved in the study.
- Please ask a member of your clinical team if there is anything that is not clear, or if you would like more information.

For peer review only - http://bmjopen.bmj.com/site/about/guidelines.xhtml

Why are we doing the FEMuR III study?

Hip fracture is a common, major health problem in old age, especially for people with other health problems or who are frail. Some patients who suffer this type of fracture need surgery to repair it. They take a long time to recover, and others may not recover fully.

Once patients are discharged, the routine care they receive can vary depending on local NHS policy. Some may not find it as easy to live independently afterwards.

We have designed an enhanced rehabilitation package for patients who are recovering from this surgery, which is delivered in addition to standard NHS care. FEMuR III will compare the enhanced package with standard NHS care to see if it can improve recovery for patients.

What is the Enhanced Rehabilitation Package?

The enhanced rehabilitation package is made specifically for each patient and we think this should improve recovery. We think this package should work better if it includes physiotherapy (to help patients recover movement), occupational therapy (to help patients with activities associated with daily living) and also provides tools to help build confidence and mood.

The enhanced rehabilitation package we have designed involves additional rehabilitation at follow up visits. You will be given a workbook and a goal-setting diary to complete during the first few months of recovery.

In order to compare the enhanced rehabilitation package to standard NHS care we are asking 446 people to take part in our study. We will follow your progress in the 12 months after surgery and collect information from you during that time so we can see how you are. The information we collect will help us to see whether there is a difference between those people who have standard NHS treatment and those who receive standard NHS treatment **and** the enhanced rehabilitation package.

Why have I been asked to take part?

We are inviting you to take part in this study because you are a patient at one of the hospitals taking part and have recently had surgery to repair a hip fracture.

What will I have to do if I take part?

A member of the clinical team can talk to you in more detail about this study and you will be able to ask any questions that you have. If you have had all of your questions answered and are happy to take part then you will be asked to sign a consent form to confirm you want to take part. You will be given a copy of your consent form and this information sheet to keep.

This study is comparing standard NHS care with the enhanced rehabilitation package. Both of these will be tailored to individual patients so it is difficult for us to describe exactly what your rehabilitation will look like. However, the main differences between the two are that if you are receiving the enhanced rehabilitation package you will also:

- Be given a goal-setting diary to complete which we would like you to use to set yourself targets and track your progress through your rehabilitation.
- Be given a workbook containing information about hip fractures: what to expect during recovery, tips to aid recovery, and useful contacts if you would like more information.
- Receive up to six community/home-based therapy sessions **in addition** to any provided by the NHS.

If you do decide to take part and have given your consent, we will ask you to complete some questionnaires and do a grip-strength test so we can get some information about how you are feeling both mentally and physically. This will allow us to see how you are recovering over time. The grip – strength test uses a special piece of equipment to measure this and will be carried out at home with a researcher.

We will arrange an appointment with you around 4 months after you have started the study to see how you are getting on. This visit may take place at your home or in a community hospital depending on what is best with you. At the visit, we will ask you to complete the same questionnaires and do some basic physical tests so we can see how you are recovering. At around 12 months after you started the study we will arrange one more visit to complete the questionnaires and physical tests again so we can track your progress. The physical tests are things you do every day. For example, the researcher will ask you to sit and stand up to five times (if you are able) and will record your progress.

For peer review only - http://bmjopen.bmj.com/site/about/guidelines.xhtml

We may also telephone you soon after the 4-month visit to ask some questions about how you are finding being involved in the study. This will give you a chance to give some feedback on your experiences and your views on the care you are receiving. Not all patients will receive a phone call, we aim to call 60 patients who have agreed to take part. We will offer you a £30 shopping voucher for your time and inconvenience in taking part in the telephone interview.

Timeline of visits

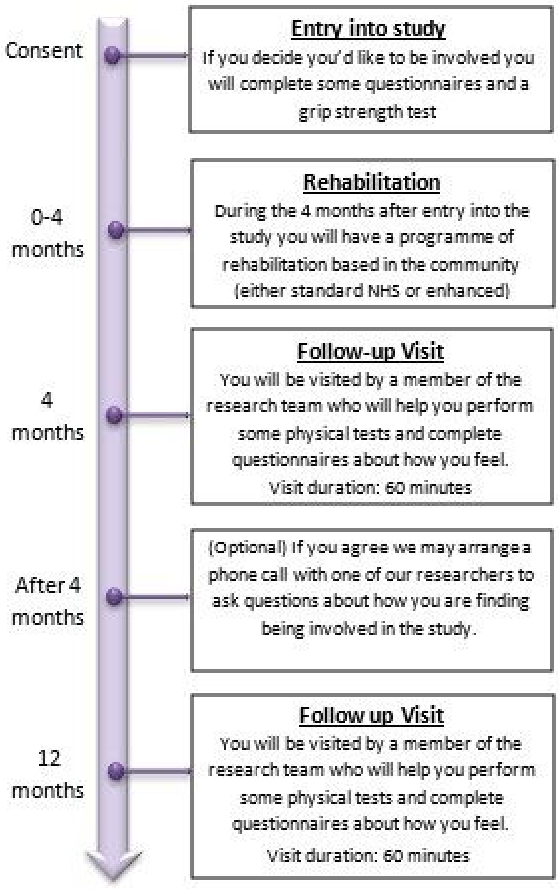

How will I know which treatment I'm going to have?

In the FEMuR III study patients will be split into two groups at random:

- One group will receive standard NHS care after discharge
- The other group will receive the enhanced rehabilitation package alongside standard NHS care after discharge

We use a computer programme that puts patients 'at random' into one of the groups – you might hear this described as 'randomisation' or 'random allocation', but they all mean the same thing. Neither you nor your doctor chooses which group you are in.

In this study you are equally likely to be in the group receiving standard NHS care as you are in the group receiving the enhanced package. Your healthcare team will let you know which group you are in as soon as possible.

What are the benefits and risks of taking part?

We are not sure whether standard NHS care or the enhanced rehabilitation package is best, but we anticipate that both will aid your recovery following surgery.

We do not foresee any significant risks involved in taking part in FEMuR III. All of the physical exercises suggested are used in normal rehabilitation after hip fracture and will be supervised by trained healthcare professionals to minimise any risk. The enhanced rehabilitation package will take up more of your time due to additional therapy sessions and having to complete the diary.

We hope that the results from the study will help doctors, therapists, patients and their carers in the future when making decisions about treatment.

What are the alternatives for treatment?

Patients recovering from hip fracture repair will get standard NHS treatment, though this may vary in different areas. In this study every patient will get standard NHS treatment even if they are in the enhanced rehabilitation group. Currently, there are no other alternative treatment programmes available.

Do I have to take part?

No, taking part is voluntary. It is up to you to decide whether or not you want to take part. If you choose to take part you can also choose to stop at any time without giving a reason. The standard of care you receive now or in the future will be the same whether you take part or not.

What happens if I change my mind?

If at any point you decide to stop taking part in the study you will receive the treatment and follow up usually offered in your area. The standard of care you receive will remain the same if you decide to stop taking part. If you do decide to stop taking part we will ask you if you would like to either:

- continue to complete follow up visits for the study
- stop taking part with no more study visits.

We will use any study information collected up until the time you stop taking part.

Will my participation be kept confidential?

Yes. All information collected about you during the course of the study will be handled according to relevant ethical and legal requirements. Your personal information will be kept strictly confidential and will only be accessed by people working on the study, or working to ensure the study is being run correctly.

You will be given a study number, which will be used along with your initials to identify you on each paper form. Your full name and date of birth, postcode, contact details and NHS number will be included on your consent form and a copy of this will be sent to the study team at the coordinating centre for the study, the Liverpool Clinical Research Centre (LCTC.) There may be instances (depending on your local NHS,) when a copy of your contact details will need to be sent to other locations or Universities within your local area to arrange your follow up visits. Only members of the FEMUR III team will be given access to your contact details, they will be held securely and destroyed after your final follow-up. Every effort will be made to ensure that any further information about you that leaves the hospital will have your name removed so that you cannot be recognised from it. This information will usually be removed by a member of the study team at your hospital/community care, but may also be removed by study team members at the LCTC upon receipt. We will also ask for your telephone number so that we can contact you for the telephone interviews during the study. Your telephone number will not be used for anything else without your consent.

With your consent, we will send a letter to your GP to let them know you are taking part and we will use your NHS number and postcode to access data about your use of health services (for example your hospital admissions).

The University of Liverpool is the sponsor for this study based in the United Kingdom. The University of Liverpool along with Bangor University will be using information from you and your medical records in order to undertake this study and will act as joint data controllers for this study. This means that both joint data controllers are responsible for looking after your information and using it properly. The University of Liverpool will keep identifiable information about you for a maximum of 25 years after the study has finished. Arrangements for confidential destruction will then be made.

Details about the use of health services (health economics) will be collected in this study. Information will be obtained from your hospital finance department and NHS Digital. Health economics researchers at the Centre for Health Economics and Medicines Evaluation (part of Bangor University), who are part of the study team, will use these data to calculate the overall costs of care. Data will be provided to the study team by NHS Digital and in order to obtain this, your NHS number will be securely transferred to NHS Digital by the CTRC using an encrypted electronic transfer system.

We would also like to collect information regarding your therapy sessions from electronic data from Therapy Management systems. This data will include date of sessions, location of sessions and activities completed during the session. This data will help us to see how many therapy sessions you have completed during the study. This data will be extracted from Therapy Management systems by NHS IT personnel and transferred using an encrypted electronic transfer system to a researcher at the University of Liverpool.

Your rights to access, change or move your information are limited, as we need to manage your information in specific ways in order for the research to be reliable and accurate. If you withdraw from the study, we will keep the information about you that we have already obtained. To safeguard your rights, we will use the minimum personally-identifiable information possible.

You can find out more about how we use your information in the "**How we use your information**" section on the study website *Femur3study.co.uk*

For peer review only - http://bmjopen.bmj.com/site/about/guidelines.xhtml

Your NHS hospital and/or community health team will collect information from you and your medical records for this research study in accordance with our instructions.

Your NHS hospital and/or community health team will use your name, NHS number and contact details to contact you about the research study, and make sure that relevant information about the study is recorded for your care, and to oversee the quality of the study. Individuals from the team and regulatory organisations may look at your medical and research records to check the accuracy of the research study. Your NHS hospital and/or community health team will pass these details to the University of Liverpool along with the information collected from you and your medical records. The only people in the University of Liverpool who will have access to information that identifies you will be people who need to confirm your participation in the study, to contact you after 4 months to ask you questions about taking part in this study or audit the data collection process. The people who analyse the information will not attempt to identify you or find out your name, NHS number or contact details.

Your NHS hospital and/or community health team will keep identifiable information about you from this study for up to a maximum of 25 years after the study has finished.

Additional information about future research:

When you agree to take part in a research study, the information about your health and care may be provided to researchers running other research studies in this organisation and in other organisations. These organisations may be universities, NHS organisations or companies involved in health and care research in this country or abroad. Your information will only be used by organisations and researchers to conduct research in accordance with the UK Policy Framework for Health and Social Care Research.

This information will not identify you and will not be combined with other information in a way that could identify you. The information will only be used for the purpose of health and care research, and cannot be used to contact you or to affect your care. It will not be used to make decisions about future services available to you, such as insurance.

What will happen to the results of the study?

We want the results of the study to be presented at conferences and published in medical journals so that we can explain to the medical, nursing and therapies' community what our research results have shown. You will not be identified in any publication or presentations.

What if there is a problem?

If you have a concern about any aspect of this study, you should ask to speak with one of your research team who will do their best to answer your questions.

If at any time during the study you feel distressed or anxious please speak to your research team, a therapist or contact your GP.

If you wish to make a formal complaint, you can do this through the NHS Complaints Procedure. Details can be obtained from your hospital.

Every care will be taken in the course of this clinical study. However, in the unlikely event that you are harmed by taking part in this research project, compensation may be available and you may have to pay your related legal costs. The University of Liverpool holds insurance against claims from participants for harm caused by their participation in this clinical trial. Participants may be able to claim compensation if they can prove that the University of Liverpool has been negligent. However, the NHS organisation that has provided your treatment has a duty of care to you, whether or not you agree to participate in the study, and the study sponsor accepts no liability for negligence on the part of your NHS organisation's employees. However, if you are harmed, and this is due to someone's negligence in the NHS, then you may have grounds for a legal action for compensation against the NHS organisation providing your treatment, but you may have to pay for your legal costs. The normal NHS complaints procedures should be available to you.

Additional information

The University of Liverpool is responsible for managing this study; they have asked that the day to day running of the study is carried out by the Liverpool Clinical Research Centre (LCTC,) part of the University of Liverpool. Additional support is provided by health economics researchers from the Centre for Health

For peer review only - http://bmjopen.bmj.com/site/about/guidelines.xhtml

Economics and Medicines Evaluation, part of Bangor University, and other researchers from participating universities (the study team).

This study is funded by the National Institute for Health Research's Health Technology Assessment Programme (ref: 16/167/09).

The study has been reviewed by the National Institute for Health Research (NIHR), Health Research Authority and the National Research Ethics Service Committee. Tyne and Wear South Ethics Committee has reviewed the study and given approval for it to take place.

The views expressed are those of the author(s) and not necessarily those of the NHS, the NIHR or the Department of Health.

Thank you for reading this information sheet.

Contacts for further information

If you would like more information or have any questions about the FEMuR III study please talk to:

Principal Investigator: <PI name to go here>

Research Nurse: <RN name to go here>

Telephone: <Hospital contact number to go here>

Or visit the website: Femur3study.co.uk

If you wish to discuss the study with someone independent of the research team you can contact the local NHS Patient Advice and Liaison Service (PALS) or local equivalent on: <Local PALS or equivalent telephone number to go here>

<<Local NHS Logo to go here>>

Fracture in the
Elderly
Multidisciplinary
Rehabilitation III

<Trust/Site address 1>
<Trust/Site address 2>
<Trust/Site address 3>
<postcode>
Tel: <telephone number>

Patient Participant Consent Form

To be completed by the Researcher:			
Site Name:			
Participant Study Number			
Patient Initials:		Participant DOB:	/ /

Once you have read and understood each statement please enter your initials in each box	Initial
Example: I confirm that I have read and understand the Participant Information Sheet.	JS
1. I have read and understood the information sheet for this study. I have had the opportunity to ask questions and have had these answered satisfactorily.	
2. I understand that participation is voluntary and that I am free to withdraw from the study at any time, without giving a reason, and without my care or legal rights being affected. I understand that in some cases further information about any unwanted effects of treatment may need to be collected by the study team.	
3. I understand that my data will be retained for a maximum of 25 years at site and by the Liverpool Clinical Trials Centre (LCTC,) part of the University of Liverpool and that they will be stored in a confidential manner.	
4. I give permission for a copy of my consent form which will include my name and date of birth, postcode and NHS number to be sent to the members of the FEMUR III research team (where it will be kept in a secure location), to allow confirmation that my consent was given.	
5. I understand that relevant sections of my medical notes and any data collected during the study may be looked at by authorised individuals from the study team and those listed under "Additional information" (NHS organisation, sponsor and regulatory authorities). I give permission for these individuals to have access to my records.	
6. I agree to allow information or results arising from this study to be used in future healthcare and/or medical research in a pseudo-anonymised form.	
7. I agree to my GP being informed of my participation in the study.	
8. I agree for my data on NHS hospital admissions and treatment to be collected for the purpose of this study and understand this will include both routine paper and electronic NHS health care records covering the study period for health economic analysis.	
9. I agree for my routine electronic data from Therapy Management systems to be collected for the purpose of this study and understand that this will include both routine paper and electronic NHS health care records covering the study period for qualitative research purposes.	
10. I agree to take part in the above study.	
**The statements below are OPTIONAL** ***(you can still participate even if you only agree to the statements above):***	
11. I agree that I may be contacted in the future in relation to this or other related studies.	

For peer review only - http://bmjopen.bmj.com/site/about/guidelines.xhtml

12. I agree to being contacted by a study researcher to conduct a qualitative interview and for the interview to be recorded.<br>(if you agree to this statement provide your details below):<br>Telephone number:		☐

Your full name (please print):			
Your signature:		Date:	
To be completed by a witness only if participant is unable to sign the consent form:			
Witness full name (please print):			
Witness signature:		Date:	

To be completed by the Researcher (on the same day after participant has completed the form):			
Researcher full name (please print):			
Researcher signature:		Date:	
Additional details required for health economic analysis:			
Participant postcode:			
Participant NHS Number:			

Appendix 2 FEMuR III Carer Participant Information Sheet and Informed Consent Forms

For peer review only - http://bmjopen.bmj.com/site/about/guidelines.xhtml

Fracture in the Elderly Multidisciplinary Rehabilitation III

<<Local NHS Logo to go here>>

<Trust/Site address 1>
<Trust/Site address 2>
<Trust/Site address 3>
<postcode>
Tel: <telephone number>

Carer Participant Information Sheet

Contents

Contents	Page
You are invited to take part in FEMuR III	1
Why are we doing the FEMuR III study?	2
What is the Enhanced Rehabilitation Package?	2
Why have I been asked to take part?	2
What will I have to do if I take part?	2
Timeline of visits	3
How will I know which treatment I'm going to have?	3
What are the benefits and risks of taking part?	3
What are the alternatives for treatment?	3
Do I have to take part?	4
What happens if I change my mind?	4
Will my participation be kept confidential?	4
What will happen to the results of the study?	5
What if there is a problem?	5
Additional information	5
Contacts for further information	6
Consent Form	7

Important Contact Information

Thank you for taking the time to read this information sheet. We hope you will find this information helpful.

If you have any questions about this study please talk to your research team:

If you would like a large print version of this information sheet please ask your research team.

<Add contact details for PI/RN i.e., name and telephone number>

Website: Femur3study.co.uk

You are invited to take part in FEMuR III

Important things to know about FEMUR III:

- FEMuR III aims to compare a new enhanced rehabilitation package with standard NHS care for patients who have had surgery to repair a hip fracture.
- After surgery, some patients take a long time to recover, and others may not recover fully.
- Additional care is often provided by members of the family or close friends, and usually involves helping hip fracture patients with activities associated with daily living or physical care.
- You have been given this information sheet as you are a carer of someone who is potentially eligible to take part.
- Before you decide whether or not to take part it is important for you to understand why the research is being done and what it will involve for you. Please take time to read the following information carefully and discuss it with friends or relatives if you wish.
- Taking part is voluntary. If you don't want to take part then you don't need to give a reason.
- Please ask a member of the clinical team if there is anything that is not clear, or if you would like more information.

Why are we doing the FEMuR III study?

The FEMuR III study hopes to collect information to help provide evidence about the best way to treat patients who are recovering from hip repair surgery after a fracture. Hip fractures are common and often need to be surgically repaired. Once patients are discharged, the routine care they receive can vary depending on local

NHS policy and they often require additional help at home from family members or friends.

FEMuR III will compare an enhanced rehabilitation programme we have designed against the normal care given by the NHS. We want to see if one offers any additional benefit over the other. To do this, we are asking 446 patients and their carers to take part. We will follow progress in the 12 months after surgery and collect information from you during that time so we can see how you both are.

What is the Enhanced Rehabilitation Package?

The enhanced rehabilitation package is made specifically for each patient and we think this should improve recovery. The enhanced rehabilitation package mixes extra therapy with self-help tools which aim to help patients improve aspects of their own recovery e.g., build confidence in trying exercises by themselves. We also hope that this will help the people caring for hip fracture patients by reducing the level of care they need to provide as patients may be able to recover independence quicker.

The enhanced rehabilitation package we have designed involves additional rehabilitation sessions and patients will be given a workbook and a goal-setting diary to complete during the first few months of recovery. Carers will be given questionnaires to complete so we can look at how much help you are providing and how you are managing.

In order to compare the enhanced rehabilitation package with standard NHS care we are asking 446 people aged 60 and over, who have had hip repair surgery, to take part in our study. We will follow their progress in the 12 months after surgery and collect information from them and from you (as their carer) during that time so we can see how you both are. The information we collect will help us to see whether there is a difference between those people who have standard NHS treatment and those who receive standard NHS treatment and the enhanced rehabilitation package.

Why have I been asked to take part?

We are inviting you to take part in this study because you are a carer of a patient who has recently had surgery to repair a hip fracture at one of the hospitals taking part

What will I have to do if I take part?

A member of the clinical team will talk to you first in more detail and you will be able to ask any questions that you have. If you have had all of your questions answered and are happy to take part then you will be asked to sign a consent form to confirm you want to participate. You will be given a copy of the consent form and the information sheet to keep.

If you do decide to take part and have given your consent, we will ask you to complete some questionnaires so we can see how you are feeling. These may take around 15 minutes to complete.

We will arrange an appointment with your friend/relative around 4 months after surgery to see how they are getting on. This will involve a member of the research team coming out to visit your friend/relative and we would like to ask that you be present too. At the visit, we will ask you to complete the same questionnaires that you completed at the start, so that we can see if there is any difference in how you are feeling. At around 12 months after the surgery we will arrange one more visit to complete the questionnaires again so that we can see if anything has changed.

We may also telephone you after the 4-month visit to ask some questions about how you are finding being involved in the study. This will give you a chance to give some feedback on your experiences as well as your views on the care your friend/relative is receiving. Not all carers will receive a phone call. We aim to call 30 carers who have agreed to take part. We will offer you a £30 shopping voucher for your time and inconvenience in taking part in the telephone interview.

For peer review only - http://bmjopen.bmj.com/site/about/guidelines.xhtml

Timeline of visits

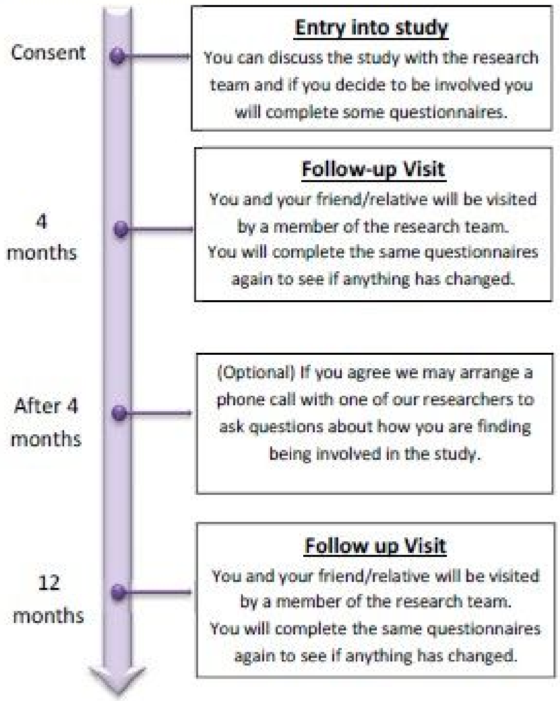

How will I know which treatment my friend/relative going to have?

In the FEMuR III study patients will be split into two groups at random:

- One group will receive normal NHS care
- The other group will receive the enhanced rehabilitation package

In the FEMuR III study participants are equally as likely to be in the group receiving normal NHS care as in the group receiving the enhanced programme. The healthcare team will let you know which group your friend/relative is in as soon as possible.

What are the benefits and risks of taking part?

We are not sure whether routine NHS care or the enhanced therapy programme is best but we anticipate that both will aid patient recovery following surgery.

We do not foresee any significant risks involved in taking part in FEMuR III, although the assessments will take up some time. It is also possible that the enhanced rehabilitation programme will take up more of your time if your friend/relative receives additional therapy sessions that you may have to support them with.

We hope that the results from the study will help doctors, therapists, patients and their carers in the future when making decisions about treatment.

What are the alternatives for treatment?

Patients recovering from hip fracture repair will get standard NHS treatment, though this may vary in different areas. In this study every patient will get standard NHS treatment even if they are in the enhanced rehabilitation group. Currently, there are no other alternative treatment programmes available.

Do I have to take part?

No, taking part is voluntary. It is up to you to decide whether or not you want to take part. If you choose to take part you can also choose to stop at any time without giving a reason. This will not affect the care received by your friend/relative or how you are treated.

What happens if I change my mind?

If at any point you decide to stop taking part in the study we will not collect any further information from you. We will use any study information collected up until the time you stop taking part.

Will my participation be kept confidential?

Yes. All information collected about you during the course of the study will be handled according to relevant ethical and legal requirements. Your personal information will be kept strictly confidential and will only be accessed by people working on the study, or working to ensure the study is being run correctly.

You will be given a study number, which will be used along with your initials to identify you on each paper form. Your full name and telephone number will be included on your consent form and a copy of this will be sent to the study team at the coordinating centre for the study, the Liverpool Clinical Trials Centre (LCTC.) There may be instances (depending on your local NHS,) when a copy of your contact details will need to be sent to other

For peer review only - http://bmjopen.bmj.com/site/about/guidelines.xhtml

locations or Universities within your local area to arrange your follow up visits. Only members of the FEMUR III team will be given access to your contact details, they will be held securely and destroyed after your final follow-up.We will ask for your telephone number so that we can contact you for the telephone interviews during the study. Your telephone number will not be used for anything else without your consent.

The University of Liverpool is the sponsor for this study based in the United Kingdom. The University of Liverpool along with Bangor University will be using information from you in order to undertake this study and act as the joint data controllers for this study. This means that both joint data controllers are responsible for looking after your information and using it properly. The University of Liverpool will keep identifiable information about you for a maximum of 25 years after the study has finished. Arrangements for confidential destruction will then be made.

Your rights to access, change or move your information are limited, as we need to manage your information in specific ways in order for the research to be reliable and accurate. If you withdraw from the study, we will keep the information about you that we have already obtained. To safeguard your rights, we will use the minimum personally-identifiable information possible.

You can find out more about how we use your information in the **"How we use your information"** section on the trial website Femur3study.co.uk

Your friend/relative's NHS hospital and/or community health team will collect information from you for this research study in accordance with our instructions.

The only people working on the studywho will have access to information that identifies you will be people who need to confirm your participation in the study, contact you after 4 months to ask you questions about taking part in this study or audit the data collection process. The people who analyse the information will not attempt to identify you or find out your name or contact details.

Your friend/relative's NHS hospital and/or community health team will keep identifiable information about you from this study for up to a maximum of 25 years after the study has finished.

Additional information about future research:

When you agree to take part in a research study, the information you give may be provided to researchers running other research studies in this organisation and in other organisations. These organisations may be universities, NHS organisations or companies involved in health and care research in this country or abroad. Your information will only be used by organisations and researchers to conduct research in accordance with the UK Policy Framework for Health and Social Care Research.

This information will not identify you and will not be combined with other information in a way that could identify you. The information will only be used for the purpose of health and care research, and cannot be used to contact you or to affect your care. It will not be used to make decisions about future services available to you, such as insurance.

What will happen to the results of the study?

It is intended that the results of the study will be presented at conferences and published in medical journals so that we can explain to the medical, nursing and therapies' community what our research results have shown. Confidentiality will be ensured at all times and you will not be identified in any publication.

What if there is a problem?

If you have a concern about any aspect of this study, you should ask to speak with one of the research team who will do their best to answer your questions.

If at any time during the study you feel distressed or anxious please speak to your research team, a therapist or contact your GP.

If you wish to make a formal complaint, you can do this through the NHS Complaints Procedure. Details can be obtained from the hospital.

Every care will be taken in the course of this clinical study. However, in the unlikely event that you are harmed by taking part in this research project, compensation may be available and you may have to pay your related legal costs. The University of Liverpool holds insurance against claims from participants for

harm caused by their participation in this clinical trial. Participants may be able to claim compensation if they can prove that the University of Liverpool has been negligent. However, if you are harmed and this is due to someone's negligence in the NHS, then you may have grounds for a legal action for compensation against the NHS, but you may have to pay for your legal costs. The normal NHS complaints procedures should be available to you. The study sponsor accepts no liability for negligence on part of your NHS organisation's employees.

Additional information

The University of Liverpool is responsible for managing this study; they have asked that the day to day running of the study is carried out by the Liverpool Clinical Trial Centre (LCTC)part of the University of Liverpool. Additional support is provided by other researchers from participating universities (the study team).

This study is funded by the National Institute for Health Research's Health Technology Assessment programme (ref: 16/167/09).

The study has been reviewed by the National Institute for Health Research (NIHR), Health Research Authority and the National Research Ethics Service Committee. Tyne and Wear South reviewed the study and given approval for it to take place.

The views expressed are those of the author(s) and not necessarily those of the NHS, the NIHR or the Department of Health.

Thank you for reading this information sheet.

Contacts for further information

If you would like more information or have any questions about the FEMuR III study please talk to:

Principal Investigator: <PI name to go here>

Research Nurse: <RN name to go here>

Telephone: <Hospital contact number to go here>

Or visit the website: Femur3study.co.uk

If you wish to discuss the study with someone independent of the research team you can contact the local NHS Patient Advice and Liaison Service (PALS) or local equivalent on: <<Local PALS or equivalent telephone number to go here>>

.

Fracture in the Elderly Multidisciplinary Rehabilitation III

<<Local NHS Logo to go here>>

<Trust/Site address 1>
<Trust/Site address 2>
<Trust/Site address 3>
<postcode>
Tel: <telephone number>

Carer Participant Consent Form

To be completed by the Researcher:			
Site Name:			
Carer Study Number		Carer Initials:	
Participant Study Number			

To be completed by the carer:

Once you have read and understood each statement please enter your initials in each box	Initial
Example: I confirm that I have read and understand the Participant Information Sheet.	JS
1. I have read and understood the information sheet for this study. I have had the opportunity to ask questions and have had these answered satisfactorily.	
2. I understand that participation is voluntary and that I am free to withdraw from the study at any time, without giving a reason, and without my care or legal rights being affected.	
3. I understand that my data will be retained for a maximum of 25 years at site and by the Liverpool Clinical Trials Centre (LCTC) part of the University of Liverpool and that they will be stored in a confidential manner.	
4. I give permission for a copy of my consent form which will include my name and telephone number to be sent to members of the FEMUR III team (where it will be kept in a secure location), to allow confirmation that my consent was given.	
5. I understand that any data collected during the study may be looked at by authorised individuals from the study team and those listed under "Additional information" (NHS organisation, sponsor and regulatory authorities). I give permission for these individuals to have access to my records.	
6. I agree to allow information or results arising from this study to be used in future healthcare and/or medical research in a pseudo-anonymised form.	
7. I agree to take part in the above study.	
**The statements below are OPTIONAL** ***(you can still participate even if you only agree to the statements above):***	
8. I agree that I may be contacted in the future in relation to this or other related studies.	
9. I agree to being contacted by a study researcher to conduct a qualitative interview and for the interview to be recorded. Telephone number: (if you agree to this statement provide your details below):	

Your full name (please print):			
Your signature:		Date:	

To be completed by the Researcher (after carer has completed the form):			
Researcher full name (please print):			
Researcher signature:		Date:	

For peer review only - http://bmjopen.bmj.com/site/about/guidelines.xhtml

Appendix 3 FEMuR III Trial Registration Data

Data category	Information
Registry and trial identification no.	ISRCTN28376407
Date of registration	23/11/2018
Funder	NIHR Evaluation, Trials and Studies Co-ordinating Centre (NETSCC); Grant code 16/167/09
Sponsor	University of Liverpool
Contact for public enquiries	LH email: femur3@liverpool.ac.uk
Scientific title	Fracture in the Elderly Multidisciplinary Rehabilitation - Phase III (FEMuR III): a definitive randomised controlled trial and economic evaluation of a community-based rehabilitation package following hip fracture
Acronym	FEMuR III
Countries of recruitment	United Kingdom
Health condition	Hip fracture
Intervention	Intervention comparator: Enhanced rehabilitation
	Control comparator: Usual care
Inclusion criteria	Aged 60 years or older
	Recent proximal hip fracture
	Surgical repair by replacement arthroplasty or internal fixation
	Living in own home prior to hip fracture
	Living and receiving rehabilitation from the NHS in the area covered by the trial centres
Exclusion criteria	Living in residential or nursing home prior to hip fracture
	Unable to understand English or Welsh
	Lacking mental capacity to give informed consent
Study design	Interventional
	Randomised controlled trial
	Treatment, education or self-management, psychological and behavioural, complex intervention, physical, rehabilitation
Recruitment start date	01/04/2019
Target sample size	446
Primary outcome	Nottingham Extended Activities of Daily Living scale
Secondary outcomes	EuroQol EQ-5D, Hospital Anxiety and Depression Scale, Abbreviated Mental Test Score, Falls Efficacy Scale – International, hip pain intensity, fear of falling, grip strength, short physical performance battery

For peer review only

For peer review only

FEMuR III SPIRIT Checklist

STANDARD PROTOCOL ITEMS: RECOMMENDATIONS FOR INTERVENTIONAL TRIA

SPIRIT 2013 Checklist: Recommended items to address in a clinical trial protocol and related documents*

Section/item	Item No	Description	Addressed on page number
**Administrative information**			
Title	1	Descriptive title identifying the study design, population, interventions, and, if applicable, trial acronym	1
Trial registration	2a	Trial identifier and registry name. If not yet registered, name of intended registry	1,3
	2b	All items from the World Health Organization Trial Registration Data Set	Appendix 3
Protocol version	3	Date and version identifier	19
Funding	4	Sources and types of financial, material, and other support	31
Roles and responsibilities	5a	Names, affiliations, and roles of protocol contributors	1,2,26
	5b	Name and contact information for the trial sponsor	30
	5c	Role of study sponsor and funders, if any, in study design; collection, management, analysis, and interpretation of data; writing of the report; and the decision to submit the report for publication, including whether they will have ultimate authority over any of these activities	29-31
	5d	Composition, roles, and responsibilities of the coordinating centre, steering committee, endpoint adjudication committee, data management team, and other individuals or groups overseeing the trial, if applicable (see Item 21a for data monitoring committee)	30,31

For peer review only

**Introduction**			
Background and rationale	6a	Description of research question and justification for undertaking the trial, including summary of relevant studies (published and unpublished) examining benefits and harms for each intervention	6,7
	6b	Explanation for choice of comparators	6,7
Objectives	7	Specific objectives or hypotheses	8
Trial design	8	Description of trial design including type of trial (eg, parallel group, crossover, factorial, single group), allocation ratio, and framework (eg, superiority, equivalence, noninferiority, exploratory)	9
**Methods: Participants, interventions, and outcomes**			
Study setting	9	Description of study settings (eg, community clinic, academic hospital) and list of countries where data will be collected. Reference to where list of study sites can be obtained	9
Eligibility criteria	10	Inclusion and exclusion criteria for participants. If applicable, eligibility criteria for study centres and individuals who will perform the interventions (eg, surgeons, psychotherapists)	9,10
Interventions	11a	Interventions for each group with sufficient detail to allow replication, including how and when they will be administered	10-12
	11b	Criteria for discontinuing or modifying allocated interventions for a given trial participant (eg, drug dose change in response to harms, participant request, or improving/worsening disease)	11,12
	11c	Strategies to improve adherence to intervention protocols, and any procedures for monitoring adherence (eg, drug tablet return, laboratory tests)	12,18-21
	11d	Relevant concomitant care and interventions that are permitted or prohibited during the trial	10,11

For peer review only - http://bmjopen.bmj.com/site/about/guidelines.xhtml

Outcomes	12	Primary, secondary, and other outcomes, including the specific measurement variable (eg, systolic blood pressure), analysis metric (eg, change from baseline, final value, time to event), method of aggregation (eg, median, proportion), and time point for each outcome. Explanation of the clinical relevance of chosen efficacy and harm outcomes is strongly recommended	12,13
Participant timeline	13	Time schedule of enrolment, interventions (including any run-ins and washouts), assessments, and visits for participants. A schematic diagram is highly recommended (see Figure)	34,35
Sample size	14	Estimated number of participants needed to achieve study objectives and how it was determined, including clinical and statistical assumptions supporting any sample size calculations	13,14
Recruitment	15	Strategies for achieving adequate participant enrolment to reach target sample size	14,15

Methods: Assignment of interventions (for controlled trials)

Allocation:

Sequence generation	16a	Method of generating the allocation sequence (eg, computer-generated random numbers), and list of any factors for stratification. To reduce predictability of a random sequence, details of any planned restriction (eg, blocking) should be provided in a separate document that is unavailable to those who enrol participants or assign interventions	15
Allocation concealment mechanism	16b	Mechanism of implementing the allocation sequence (eg, central telephone; sequentially numbered, opaque, sealed envelopes), describing any steps to conceal the sequence until interventions are assigned	15
Implementation	16c	Who will generate the allocation sequence, who will enrol participants, and who will assign participants to interventions	15
Blinding (masking)	17a	Who will be blinded after assignment to interventions (eg, trial participants, care providers, outcome assessors, data analysts), and how	15,16

	17b	If blinded, circumstances under which unblinding is permissible, and procedure for revealing a participant's allocated intervention during the trial	15,16

Methods: Data collection, management, and analysis

Data collection methods	18a	Plans for assessment and collection of outcome, baseline, and other trial data, including any related processes to promote data quality (eg, duplicate measurements, training of assessors) and a description of study instruments (eg, questionnaires, laboratory tests) along with their reliability and validity, if known. Reference to where data collection forms can be found, if not in the protocol	12,13
	18b	Plans to promote participant retention and complete follow-up, including list of any outcome data to be collected for participants who discontinue or deviate from intervention protocols	32-34
Data management	19	Plans for data entry, coding, security, and storage, including any related processes to promote data quality (eg, double data entry; range checks for data values). Reference to where details of data management procedures can be found, if not in the protocol	16,17
Statistical methods	20a	Statistical methods for analysing primary and secondary outcomes. Reference to where other details of the statistical analysis plan can be found, if not in the protocol	16,17
	20b	Methods for any additional analyses (eg, subgroup and adjusted analyses)	17-21
	20c	Definition of analysis population relating to protocol non-adherence (eg, as randomised analysis), and any statistical methods to handle missing data (eg, multiple imputation)	16,17

Methods: Monitoring

Data monitoring	21a	Composition of data monitoring committee (DMC); summary of its role and reporting structure; statement of whether it is independent from the sponsor and competing interests; and reference to where further details about its charter can be found, if not in the protocol. Alternatively, an explanation of why a DMC is not needed	21,31
	21b	Description of any interim analyses and stopping guidelines, including who will have access to these interim results and make the final decision to terminate the trial	16,31
Harms	22	Plans for collecting, assessing, reporting, and managing solicited and spontaneously reported adverse events and other unintended effects of trial interventions or trial conduct	21
Auditing	23	Frequency and procedures for auditing trial conduct, if any, and whether the process will be independent from investigators and the sponsor	21
**Ethics and dissemination**			
Research ethics approval	24	Plans for seeking research ethics committee/institutional review board (REC/IRB) approval	21
Protocol amendments	25	Plans for communicating important protocol modifications (eg, changes to eligibility criteria, outcomes, analyses) to relevant parties (eg, investigators, REC/IRBs, trial participants, trial registries, journals, regulators)	21
Consent or assent	26a	Who will obtain informed consent or assent from potential trial participants or authorised surrogates, and how (see Item 32)	14,15
	26b	Additional consent provisions for collection and use of participant data and biological specimens in ancillary studies, if applicable	N/A
Confidentiality	27	How personal information about potential and enrolled participants will be collected, shared, and maintained in order to protect confidentiality before, during, and after the trial	14,15, Appendices 1&2
Declaration of interests	28	Financial and other competing interests for principal investigators for the overall trial and each study site	31

For peer review only

Access to data	29	Statement of who will have access to the final trial dataset, and disclosure of contractual agreements that limit such access for investigators	22
Ancillary and post-trial care	30	Provisions, if any, for ancillary and post-trial care, and for compensation to those who suffer harm from trial participation	31
Dissemination policy	31a	Plans for investigators and sponsor to communicate trial results to participants, healthcare professionals, the public, and other relevant groups (eg, via publication, reporting in results databases, or other data sharing arrangements), including any publication restrictions	22
	31b	Authorship eligibility guidelines and any intended use of professional writers	22
	31c	Plans, if any, for granting public access to the full protocol, participant-level dataset, and statistical code	22
**Appendices**			
Informed consent materials	32	Model consent form and other related documentation given to participants and authorised surrogates	Appendices 1&2
Biological specimens	33	Plans for collection, laboratory evaluation, and storage of biological specimens for genetic or molecular analysis in the current trial and for future use in ancillary studies, if applicable	N/A

*It is strongly recommended that this checklist be read in conjunction with the SPIRIT 2013 Explanation & Elaboration for important clarification on the items. Amendments to the protocol should be tracked and dated. The SPIRIT checklist is copyrighted by the SPIRIT Group under the Creative Commons "Attribution-NonCommercial-NoDerivs 3.0 Unported" license.

For peer review only

BMJ Open

Protocol for a definitive randomised controlled trial and economic evaluation of a community-based rehabilitation programme following hip fracture: Fracture in the Elderly Multidisciplinary Rehabilitation - Phase III (FEMuR III) [ISRCTN28376407]

Journal:	*BMJ Open*
Manuscript ID	bmjopen-2020-039791.R1
Article Type:	Protocol
Date Submitted by the Author:	08-Jun-2020

Complete List of Authors:	Williams, Nefyn; University of Liverpool Faculty of Health and Life Sciences, Primary Care and Mental Health<br>Dodd, Susanna; University of Liverpool Faculty of Health and Life Sciences, Liverpool Clinical Trials Centre<br>Hardwick, Ben; University of Liverpool Faculty of Health and Life Sciences, Liverpool Clinical Trials Centre<br>Clayton, Dannii; University of Liverpool Faculty of Health and Life Sciences, Liverpool Clinical Trials Centre<br>Edwards, Rhiannon; Bangor University College of Physical and Applied Sciences, Centre for Health Economics & Medicines Evaluation<br>Charles, Joanna; Bangor University College of Health and Behavioural Sciences, Centre for Health Economics & Medicines Evaluation<br>Logan, Phillipa; University of Nottingham Faculty of Medicine and Health Sciences, Community Health Sciences<br>Busse, Monica; Cardiff University Centre for Trials Research<br>Lewis, Ruth; Bangor University College of Health and Behavioural Sciences, North Wales Centre for Primary Care Research<br>Smith, Toby; University of East Anglia Faculty of Medicine and Health Sciences<br>Sackley, Catherine; University of Nottingham Faculty of Medicine and Health Sciences<br>Morrison, Val; Bangor University College of Health and Behavioural Sciences, School of Psychology<br>Lemmey, Andrew; Bangor University College of Health and Behavioural Sciences, School of Sports, Health and Exercise Science<br>Masterson-Algar, Patricia; Bangor University College of Health and Behavioural Sciences, School of Healthcare Sciences<br>Howard, Lola; University of Liverpool Faculty of Health and Life Sciences, Liverpool Clinical Trials Centre<br>Hennessy, Sophie; University of Liverpool Faculty of Health and Life Sciences, Liverpool Clinical Trials Centre<br>Soady, Claire; University of Liverpool Faculty of Health and Life Sciences, Liverpool Clinical Trials Centre<br>Ralph, Penelope; University of Liverpool Faculty of Health and Life Sciences, Department of Primary Care and Mental Health<br>Dobson, Susan; University of Liverpool Faculty of Health and Life Sciences, Primary Care and Mental Health

	Dorkenoo, Shanaz; Health and Care Research Wales, Involving People Network
**Primary Subject Heading**:	Rehabilitation medicine
Secondary Subject Heading:	Geriatric medicine, Health economics, Health services research
Keywords:	REHABILITATION MEDICINE, Hip < ORTHOPAEDIC & TRAUMA SURGERY, Clinical trials < THERAPEUTICS, HEALTH ECONOMICS, QUALITATIVE RESEARCH

SCHOLARONE™ Manuscripts

I, the Submitting Author has the right to grant and does grant on behalf of all authors of the Work (as defined in the below author licence), an exclusive licence and/or a non-exclusive licence for contributions from authors who are: i) UK Crown employees; ii) where BMJ has agreed a CC-BY licence shall apply, and/or iii) in accordance with the terms applicable for US Federal Government officers or employees acting as part of their official duties; on a worldwide, perpetual, irrevocable, royalty-free basis to BMJ Publishing Group Ltd ("BMJ") its licensees and where the relevant Journal is co-owned by BMJ to the co-owners of the Journal, to publish the Work in this journal and any other BMJ products and to exploit all rights, as set out in our licence.

The Submitting Author accepts and understands that any supply made under these terms is made by BMJ to the Submitting Author unless you are acting as an employee on behalf of your employer or a postgraduate student of an affiliated institution which is paying any applicable article publishing charge ("APC") for Open Access articles. Where the Submitting Author wishes to make the Work available on an Open Access basis (and intends to pay the relevant APC), the terms of reuse of such Open Access shall be governed by a Creative Commons licence – details of these licences and which Creative Commons licence will apply to this Work are set out in our licence referred to above.

Other than as permitted in any relevant BMJ Author's Self Archiving Policies, I confirm this Work has not been accepted for publication elsewhere, is not being considered for publication elsewhere and does not duplicate material already published. I confirm all authors consent to publication of this Work and authorise the granting of this licence.

For peer review only

Protocol for a definitive randomised controlled trial and economic evaluation of a community-based rehabilitation programme following hip fracture: Fracture in the Elderly Multidisciplinary Rehabilitation - Phase III (FEMuR III) [ISRCTN28376407]

Nefyn H Williams[1]

Email: Nefyn.williams@liverpool.ac.uk

Susanna Dodd[1]

Email: shinds@liverpool.ac.uk

Ben Hardwick[1]

Email: ben121@liverpool.ac.uk

Dannii Clayton[1]

Email: claytond@liverpool.ac.uk

Rhiannon T Edwards[2]

Email: r.t.edwards@bangor.ac.uk

Joanna M Charles[2]

Email: j.charles@bangor.ac.uk

Phillipa Logan[3]

Email: Pip.Logan@nottingham.ac.uk

Monica Busse[4]

Email: BusseME@cardiff.ac.uk

Ruth Lewis[2]

Email: r.lewis@bangor.ac.uk

Toby Smith[5]

Email: toby.smith@uea.ac.uk

Catherine Sackley[3]

For peer review only

Email: catherine.sackley@nottingham.ac.uk

Val Morrison[2]

Email: v.morrison@bangor.ac.uk

Andrew B Lemmey[2]

Email: a.lemmey@bangor.ac.uk

Patricia Masterson Algar[2]

Email: p.m.algar@bangor.ac.uk

Lola Howard[1]

Email: awoyalel@liverpool.ac.uk

Sophie Hennessy[1]

Email: sophie.hennessy@liverpool.ac.uk

Claire Soady[1]

Email: claire.soady@liverpool.ac.uk

Penelope Ralph[1]

Email: p.ralph@liverpool.ac.uk

Susan Dobson[1]

Email: sldobson@liverpool.ac.uk

Shanaz Dorkenoo[6]

Email: shanaz519@btinternet.com

[1]University of Liverpool, UK

[2]Bangor University, UK

[3]University of Nottingham, UK

[4]Cardiff University, UK

[5]University of East Anglia, UK

For peer review only

For peer review only - http://bmjopen.bmj.com/site/about/guidelines.xhtml

[6]Involving People, Cardiff, UK

Corresponding author: Professor Nefyn H Williams, Department of Health Services Research, University of Liverpool, 1st Floor, Block B, Waterhouse Building, 1-5 Brownlow Street, Liverpool, L69 3GL, UK.

Tel: +44 (0)151 795 5305 e-mail: nefyn.williams@liverpool.ac.uk

Word count (4,000)

For peer review only

For peer review only - http://bmjopen.bmj.com/site/about/guidelines.xhtml

Abstract (300 words)

Introduction

Proximal femoral (hip) fracture is common, serious and costly. Rehabilitation may improve functional recovery but evidence of effectiveness and cost-effectiveness is lacking. An enhanced rehabilitation intervention was previously developed and a feasibility study tested the methods used for this randomised controlled trial (RCT).

The objectives are to compare the effectiveness and cost-effectiveness of the enhanced rehabilitation programme following surgical repair of proximal femoral fracture in older people compared with usual care.

Methods and analysis

Protocol for phase III, parallel-group, two-armed, superiority, pragmatic RCT with 1:1 allocation ratio. Allocation sequence by minimisation programme with a built in random element. Secure web-based allocation concealment. The two treatments will be usual care (control) and usual care plus an enhanced rehabilitation programme (intervention). The enhanced rehabilitation will consist of a patient-held information workbook, goal-setting diary and up to six additional therapy sessions. Outcome assessment and statistical analysis will be performed blind; patient and carer participants will be unblinded. Outcomes will be measured at baseline, 17 and 52 weeks' follow-up. Primary outcome at 52 weeks will be the Nottingham Extended Activities of Daily Living scale. Secondary outcomes will measure anxiety and depression, health utility, cognitive status, hip pain intensity, falls self-efficacy, fear of falling, grip strength and physical function. Carer strain, anxiety and depression will be measured in carers. All safety events will be recorded, and serious adverse events will be assessed to determine whether they are related to the intervention and expected.

For peer review only - http://bmjopen.bmj.com/site/about/guidelines.xhtml

Concurrent economic evaluation will be a cost-utility analysis from a health service and personal social care perspective. An embedded process evaluation will determine the mechanisms and processes that explain the implementation and impacts of the enhanced rehabilitation programme.

Ethics and dissemination

NHS research ethics approval reference 18/NE/0300. Results will be disseminated by peer-reviewed publication.

Registration details

ISRCTN28376407 registered on 23/11/2018.

Article Summary

Strengths and limitations of this study

- Pragmatic phase III randomised controlled trial following phase I intervention development and phase II feasibility study.
- Concurrent economic evaluation with a health service and personal social care perspective.
- Embedded process evaluation to determine the mechanisms and processes that explain the implementation and impacts of the enhanced rehabilitation programme.
- Only patients with mental capacity to consent are eligible, therefore excluding a large number of potential participants lacking capacity.

For peer review only

For peer review only - http://bmjopen.bmj.com/site/about/guidelines.xhtml

Introduction

Proximal femoral fracture, more commonly referred to as hip fracture, is a common, major health problem in old age [1]. It is projected to increase further as the population ages [2,3]. Mortality is high [4,5], and of those who survive to one year, 29% fail to regain their level of functioning, in terms of restrictions of activities of daily living [6]; many lose their independence. This imposes a large cost burden on society, estimated to be approximately £2.3 billion a year in the United Kingdom [7]. The majority of costs are incurred in the community and social care setting in the 12 months following hospital discharge, which are almost four times higher than the costs of the acute hospital admission [8]. Frail individuals are at particular risk of secondary future proximal femoral fracture, resulting in worse morbidity and mortality outcomes [9].

The National Institute of Health and Clinical Excellence (NICE) have issued guidelines for the management of hip fracture [10]. This includes the provision of a co-ordinated multidisciplinary rehabilitation programme starting in hospital during post-operative recovery and continuing in the community following discharge [10]. Where possible such rehabilitation programmes should consider individual patient goals, facilitate a return to pre-fracture independence and provide patients and carers with written information to support the rehabilitation programme and long-term outcomes. The Hip Sprint audit reported that community rehabilitation services were inconsistent [11].

Rationale

There have been four relevant Cochrane systematic reviews with inconclusive results [12-15]. These have examined different types and intensities of in-patient rehabilitation [12],

For peer review only - http://bmjopen.bmj.com/site/about/guidelines.xhtml

mobilisation strategies [13], psychosocial functioning after hip fracture [14] and rehabilitation for those with dementia following hip fracture surgery [15]. Other systematic reviews have reported improved walking ability [16], strength and physical function [17], including those with mild to moderate dementia [18]. These systematic reviews concluded that whilst individual components of rehabilitation programmes may aid recovery after a hip fracture, there is insufficient evidence to demonstrate clinical effectiveness or cost-effectiveness of an overall care pathway, and that further research is required.

A previous study [19] completed the first two phases of the Medical Research Council (MRC) framework for complex interventions [20]. The first phase developed an enhanced rehabilitation intervention which, in addition to usual care, included a patient-held workbook, a goal-setting diary and up to six additional home-based therapy sessions [21]. The second phase of the study was a randomised feasibility study, which assessed the acceptability of the new rehabilitation programme and the feasibility of trial methods for a definitive phase III randomised controlled trial (RCT) [22, 23]. Although this feasibility study was underpowered to assess effectiveness, the intervention showed a medium sized improvement in the Nottingham Extended Activities of Daily Living scale compared with usual care (Cohen's d 0.63). A process evaluation described the implementation of the rehabilitation programme and informed how to enhance recruitment and improve the intervention [24].

Risk and Benefits

The enhanced rehabilitation programme demonstrated a potential improvement in activities of daily living in the feasibility study. Possible risks of rehabilitation interventions

For peer review only - http://bmjopen.bmj.com/site/about/guidelines.xhtml

would include injury or falling when performing therapeutic exercises, which must be weighed against the risk to health of sedentary behaviour.

Primary Objective

To determine the effectiveness of an enhanced rehabilitation programme following surgical repair of proximal femoral fracture in older people compared with usual care, in terms of the performance of activities of daily living at 52 weeks follow-up.

Secondary Objectives

1. To compare the cost-effectiveness of an enhanced rehabilitation programme following surgical repair of proximal femoral fracture in older people compared with usual care at 52 weeks follow-up.
2. To determine the effectiveness of an enhanced rehabilitation programme following surgical repair of proximal femoral fracture in older people compared with usual care, in terms of the performance of activities of daily living at 17 weeks follow-up.
3. To determine the effectiveness of an enhanced rehabilitation package following surgical repair of proximal femoral fracture in older people compared with usual care, in terms of anxiety and depression at 17 and 52 weeks follow-up.
4. To assess whether the enhanced rehabilitation intervention creates change in self-efficacy, hip pain, cognitive function, fear of falling and physical function as potential mediators for improving activities of daily living at 17 and 52 weeks follow-up.
5. To assess whether the enhanced rehabilitation intervention creates change in strain, anxiety and depression in carers at 17 and 52 weeks follow-up.

For peer review only - http://bmjopen.bmj.com/site/about/guidelines.xhtml

6. To determine the mechanisms and processes that explain the implementation and impacts of the enhanced rehabilitation programme, and whether there are adverse effects.

Methods and Analysis

Trial design

This is a pragmatic, multisite, parallel-group, two-armed, superiority randomised controlled trial (RCT) with 1:1 allocation ratio, and an internal pilot phase (Figure 1). Outcome assessment and statistical analysis will be blinded; patient and carer participants and clinicians will be unblinded. A concurrent economic evaluation will be a cost-utility analysis from a health service and personal social care perspective. An embedded process evaluation will examine the mechanisms and processes that explain the implementation and impacts of the enhanced rehabilitation programme. The RCT was registered on 23 November 2018, ISRCTN28376407. Trial registration data can be found in Appendix 1 in the supplementary file.

Trial Setting and Selection of Sites / Clinicians

Sites were recruited by co-investigators in different regions of England and Wales with a spread of socio-economic conditions and a mixture of rural and urban locations: Kent (CS), Merseyside (NW), Norwich (TS), north Wales (RL), Nottingham (PL) and south Wales (MB). The sites had to include trauma centres treating proximal femoral fracture and links to community rehabilitation teams, which could accommodate the extra community rehabilitation sessions.

For peer review only - http://bmjopen.bmj.com/site/about/guidelines.xhtml

Patients will be recruited on orthopaedic, rehabilitation and community hospital wards, or after hospital discharge home. The intervention will be delivered in the community, following hospital discharge, by community teams receiving referrals from the acute hospital sites and their associated community hospitals.

Selection of Sites/Clinicians

Sites have been opened to recruitment in Nottingham, Norfolk, north Wales, south Wales and east Kent. Further sites are planned in west Kent, Derby and Cheshire plus others. The site trial teams comprise principal investigators, hospital and community NHS staff, research assistants and support staff from clinical research networks.

For peer review only

Trial Population

Inclusion Criteria

1. Age 60 years or older
2. Recent proximal femoral fracture
3. Surgical repair by replacement arthroplasty, hemi-arthroplasty or internal fixation
4. Living in their own home prior to hip fracture
5. Living and receiving rehabilitation from the NHS in the area covered by the trial sites

Exclusion Criteria

1. Living in residential or nursing homes prior to hip fracture
2. Participants unable to understand English or Welsh
3. Lacking mental capacity to give informed consent

Carer Participants

We will also recruit carer participants to evaluate carer strain, anxiety and depression. These are defined as a relative or friend providing help with activities of daily living or physical

For peer review only - http://bmjopen.bmj.com/site/about/guidelines.xhtml

care, at least four days a week. Carer participants will provide informed consent, but will not receive any trial intervention, so will not undergo eligibility screening or randomisation.

Trial Treatment/interventions

We plan to compare an enhanced rehabilitation intervention with usual rehabilitation care.

Usual rehabilitation care

Usual care consists of a multi-disciplinary rehabilitation delivered by the acute hospital, community hospital and community services depending on patients' individual needs at different times during their recovery and on the availability and accessibility of services in different areas. The multidisciplinary team delivering care and rehabilitation may include: orthopaedic surgeons, orthogeriatricians, nurses, physiotherapists, occupational therapists, dieticians, pharmacists, GPs and social workers. The settings for care include acute orthopaedic or orthogeriatric wards, rehabilitation units in community hospitals, rehabilitation beds in care homes, the patient's own home and care home settings, all delivered by a variety of community teams in both health and social care services. There will be no restrictions on concomitant medications or treatments.

Enhanced rehabilitation

The main aim of the intervention is to enhance usual rehabilitation by increasing patients' self-efficacy [25], and increasing the amount and quality of patients' practice of physical exercise and activities of daily living to improve functional outcomes at follow-up. Self-efficacy will be enhanced by means of a patient-held information workbook and a goal-setting diary. The workbook will include:

- Information about what has happened to them, and what to expect from their recovery;

For peer review only - http://bmjopen.bmj.com/site/about/guidelines.xhtml

- Information about NHS, council and voluntary sector services including falls' prevention programmes;
- How to manage their recovery, set goals and monitor progress of their rehabilitation; reduce fear of falling.

In addition to whatever community-based rehabilitation is provided as part of usual care, we will provide up to six additional therapy sessions, once patients are discharged home. These can be delivered by physiotherapists, occupational therapists or their assistants, who have been trained to deliver these extra sessions alongside the workbook, using the diary to set goals and monitor progress. The therapists will tailor these extra sessions, so that the total number of sessions used, the time scale for their delivery, and the sessions' content will vary between patients according to need, but may include the practice of specific exercises and activities of daily living. Throughout the running of this trial, therapists will receive on-going support via e-mails, newsletters and refresher events.

Outcomes

Patient participants will complete outcome measures at baseline, 17 and 52 weeks administered by a research assistant blinded to participant allocation. Follow-up assessments will be completed within participants' homes (Tables 1 and 2). The primary outcome will be the difference in Nottingham Extended Activities of Daily Living (NEADL) scale [26,27] at 52 week follow-up, between the usual rehabilitation arm and the enhanced rehabilitation arm. At baseline, the patient will be asked to recall the four weeks prior to hip fracture and not four weeks prior to completing this questionnaire. Secondary outcomes will include the Hospital Anxiety and Depression Scale (HADS) [28], economic measures will be EuroQol EQ-5D-3L [29] and Client Service Receipt Inventory (CSRI) [30]. A reduced version of

For peer review only - http://bmjopen.bmj.com/site/about/guidelines.xhtml

this will be used at baseline to reduce participant burden as they recover from hip fracture surgery. Potential mediators of outcome will include a Visual Analogue Scale (VAS) for hip pain intensity [31], Falls Efficacy Scale - International (FES-I) (self efficacy) [32,33], and Visual Analogue Score - Fear of Falling (VASFoF) [34].

The research assistant will assess patient participants' cognitive function at baseline, 17 and 52 weeks using the Abbreviated Mental Test Score (AMTS) [35]. The research assistant will measure physical function at baseline, 17 and 52 weeks using the grip strength test [36-38], and using the Short Physical Performance Battery (SPPB) [39,40] at 17 and 52 weeks. Carer participants will complete the Caregiver Strain Index (CSI) [41] and the Hospital Anxiety and Depression Scale (HADS) [28] at baseline, 17 and 52 weeks.

Qualitative interviews will take place with patients and carers after 17 weeks. These will gather data on trial participation and intervention design (see process evaluation below).

Routinely collected demographic, clinical and recruitment data will include the numbers of patients who are eligible, willing to be randomised, withdraw after randomisation, complete outcome measurements, also reasons for non-completion, age, gender, hip fracture type, surgery type, co-morbid conditions, place of residence prior to admission and place of discharge.

Sample size calculation

The phase II feasibility study results [23] informed the sample size calculation. The adjusted mean difference in the primary outcome measure (NEADL) between the intervention and

For peer review only - http://bmjopen.bmj.com/site/about/guidelines.xhtml

control group in the feasibility trial was 3.0. Work completed by Wu, et al [27] has suggested that the minimum clinically significant difference is 2.4; this has been used within the sample size calculation for this phase III RCT. A two-point score in the NEADL scale would equate to an improvement in function from being independent around the home to being able to use public transport or get in and out of a car. The adjusted mean difference between the groups in NEADL in the randomised feasibility study had a standard deviation (SD) of 5.8. In this multi-site phase III RCT, a more diverse sample would be expected, so a larger SD would be expected. Parker et al. [42] used NEADL in a rehabilitation RCT and found a SD of 10. Based on ANCOVA with alpha of 5% and 90% power to detect a difference of 2.4 (SD = 10, R^2 of covariate = 0.52) 352 patient participants would be required to complete the trial over both treatment groups. When considering the 79% retention rate in the feasibility study [23], the trial would need to recruit 446 patient participants.

Recruitment and Randomisation

Screening and Consent – Patient Participants

Patients with proximal femoral fracture will be identified and screened for eligibility, including mental capacity, by clinical staff on orthopaedic or rehabilitation wards. If the patients are eligible, and interested in the trial, the trial team researchers would then recruit patients following the trial's informed consent process. Assessment of eligibility may occur over an extended period, if for example, the patient is experiencing temporary delirium post-surgery. If during this period, patients are transferred to rehabilitation wards, community hospitals, or discharged home, then assessment will continue in these alternative locations. These assessments will be recorded in a screening log, including any reasons for ineligibility.

For peer review only - http://bmjopen.bmj.com/site/about/guidelines.xhtml

Informed consent - carer participants

For the purpose of this RCT, carers are defined as either a relative or friend caring for a hip fracture patient, helping them with activities of daily living or physical care on at least four days a week. They will be identified and recruited following the trial's informed consent process. Copies of the participant information sheets and informed consent forms can be found in Appendices 2 and 3 in the supplementary file.

Randomisation Procedures

Patient participants who provide informed consent will complete baseline outcome measurements prior to randomisation. Randomisation will take place no later than six weeks after hip fracture repair surgery. The randomisation will have an allocation ratio of 1:1 within each stratum and across the trial. Randomisation will use a minimisation programme with a built in random element utilising factors that will not be made known to individuals in charge of recruitment to minimise any potential for predicting allocation. Randomisation will be completed by secure web access to the remote randomisation site at the clinical trials unit. The therapists delivering the enhanced rehabilitation intervention will receive an automated email when a participant has been allocated to the intervention group.

Blinding

This is a pragmatic trial comparing two rehabilitation interventions. It will therefore not be possible to blind participants or their clinicians to treatment group allocation. The research assistants will collect outcome measurements blind to treatment allocation. They will not be informed to which group the patient participants have been allocated, and will not be present at any of the therapy sessions. Before any home visits for follow-up assessments, they will ask participants not to reveal their treatment allocation. After the final follow-up

For peer review only - http://bmjopen.bmj.com/site/about/guidelines.xhtml

assessment, they will complete a perception of allocation form, in order to monitor the level of blinding achieved for these researchers. Data analysis will be performed blind to treatment allocation.

Internal pilot

An internal pilot assessed site recruitment and participant recruitment and retention rates for the six months after the first site was open to recruitment from September 2019 to February 2020.

Progression criteria

- Number of sites open: 7 or more (go); 5-6 (amend); 4 or fewer (stop)
- Open site recruitment rate per month: 2 or more (go); 1-2 (amend); <1 (stop)
- Retention rate: 69% or higher (go); 50-68% (amend); 49% or fewer (stop)

Statistical Analysis

Final analysis will take place once all participants have been followed-up for 52 weeks, and the database has been locked. Analyses will be by 'intention to treat' for the primary and secondary outcomes on all randomised participants, in the group to which they were allocated, and for whom the outcomes of interest have been observed or measured.

Baseline

Demographic and baseline characteristics will be summarised separately using descriptive statistics for each randomised group to allow clinical assessment of whether balance was achieved between randomised groups. No statistical testing of differences between groups will be performed.

For peer review only - http://bmjopen.bmj.com/site/about/guidelines.xhtml

Analysis of effectiveness

Primary and secondary outcomes at baseline, 17 weeks' and 52 weeks' follow-up will be summarised for each treatment group using descriptive statistics at each time point. If normally distributed, the difference between group means (with 95% confidence intervals) will be reported from an analysis of covariance (ANCOVA) adjusted for baseline score and stratification factors.

Missing data and withdrawals

Predictors of missing data will be investigated using regression models (including type of surgery, age, living arrangements and co-morbidities) and any significant predictors will be considered for inclusion in the models. In addition, given the two assessment points at 17 and 52 weeks, we will carry out a sensitivity analysis using a joint modelling approach to check whether there is any difference in outcome (here the longitudinal outcome rather than the outcome at 17 weeks or 52 weeks alone) between the randomised arms adjusted for dropouts or missing values.

Instrumental variable regression

The impact of engagement with the intervention will be assessed using instrumental variable (IV) regression, using the number of face-to-face direct rehabilitation sessions over 52 weeks' follow up as a continuous measure of engagement. Additional exploratory IV regression analyses will use in turn: the total number of rehabilitation sessions (face-to-face plus telephone), total time (in minutes) spent in face-to-face direct rehabilitation sessions, and total time (in minutes) spent in all rehabilitation sessions (i.e. face-to-face and telephone). The suitability of using randomisation as the instrument in these IV regression

For peer review only - http://bmjopen.bmj.com/site/about/guidelines.xhtml

models will be assessed using tests of exogeneity, redundancy and under/weak identification.

Mediation analyses

The hypothesised mechanism of change for the enhanced rehabilitation intervention is that participants' primary outcome (activities of daily living) is mediated by self-efficacy, hip pain, cognitive function, fear of falling and physical function. If the enhanced rehabilitation intervention has a significant effect on primary outcome ($p<0.05$) for enhanced rehabilitation in ANCOVA, causal mediation analysis will be used to determine whether each of these potential mediators predict change in NEADL at 52 weeks. Initial assessments will determine whether the randomised intervention affects each putative mediator in turn. For those putative mediators that are significantly ($p<0.1$) affected by the randomised intervention, mediation analysis will be carried out adjusting for baseline covariates that predict both the mediator and NEADL, potentially including type of surgery, age, living arrangements (alone/with others) and co-morbidities. Sensitivity analyses will assess the potential impact of unmeasured confounding between the mediator and NEADL.

Economic analysis

The enhanced rehabilitation programme will be fully costed using unit costs from a public sector multiagency perspective. Unit costs will be obtained from national sources of reference costs [43,44] and applied to information received from pilot questionnaires, namely salary band of therapists, time spent with the patient conducting rehabilitation, costs of travel and costs of any additional equipment. Costs of health and social care services used by the participants will also be costed using national sources of reference

For peer review only - http://bmjopen.bmj.com/site/about/guidelines.xhtml

costs. The costs of service use and the cost of the intervention will be added together for use in a cost-effectiveness analysis.

The EQ-5D (3L) will be used to calculate Quality Adjusted Life Years (QALYs) over the 52 week trial period, using the area under the curve method [45,46]. A cost-utility analysis will be conducted to calculate a cost per QALY of the enhanced rehabilitation intervention. This cost per QALY generated will be compared to the NICE threshold range of £30,000 per QALY [47]. We will bootstrap differences in costs and outcomes (EQ-5D-3L) between the two groups, producing a 95% confidence interval around these differences.

Process Evaluation

The process evaluation will aim to identify and explain all mechanisms and processes (i.e. the intervention theory) that enabled or acted as a barrier to the implementation of the enhanced rehabilitation intervention. The process evaluation will help build a picture of how the intervention was carried out in reality, and what factors shaped it. By carrying out a process evaluation, it will be possible to identify if observed impacts are solely due to the enhanced rehabilitation programme, or if these impacts are a result of a number of external and internal variables that are closely linked to the environment and the context in which the intervention takes place [48-51].

The specific objectives will be to:

- Refine the programme theory from the previous realist review that was used to develop the intervention [21]. This programme theory will explain how the researchers envisage the intervention to work, to reach its expected outcomes.

For peer review only

- Investigate therapists' expectations and experience of implementation, their previous experience and training, and their learning throughout the conduct of the trial.
- Investigate the mechanisms driving and shaping the tailoring of the enhanced rehabilitation intervention to individual patients.
- Investigate trial participants' (patients and carers) experiences and views about their involvement in the trial, as well as their experience of care in either arm of the trial.
- Map and synthesise all data collected in order to test the refined programme theory and explain the trial findings.

Process evaluation data collection

Semi-structured telephone interviews will be conducted with:

- A purposive sample of 60 patient participants in each of the two trial arms and up to 30 of their carers. Patients will be purposively sampled to ensure diversity based on age, functional impairment (using baseline NEADL scores) and the presence or absence of a family carer. Interviews will take place after the 17-week assessment and will be audio recorded.
- The therapists delivering the enhanced rehabilitation programme, which will explore implementation from the therapists' perspectives. Interviews will be conducted midway through their involvement in the trial, and at the end, in order to investigate learning over time.

Data on intervention delivery and adherence:

- Therapists will record key reflections on 'critical incident reports'.

For peer review only - http://bmjopen.bmj.com/site/about/guidelines.xhtml

- The visiting therapist will record the length and content of each extra rehabilitation therapy session on a case report form.
- All patient participants will be given a therapy session record, where visiting therapists will record the number, length and content of usual rehabilitation care. Whenever possible, routinely collected electronic data that therapists complete on their Therapy Manager system, or its equivalent, will be collected.
- An online questionnaire will be emailed to participating therapists in order to capture therapists' relevant training, previous experience and familiarity with the trial intervention.

Qualitative data will be analysed following a thematic analysis approach [52] that will be guided by the proposed programme theory. Quantitative data (record forms and online questionnaires) will be analysed using descriptive statistics, which will allow the exploration of frequency of responses. All data sets will be synthesised in order to describe the complex nature of the enhanced rehabilitation intervention.

Patient and Public Involvement

There has been patient and public involvement (PPI) at all stages including refining the research question, choosing outcomes relevant to patients, commenting on the burden of the intervention and of trial participation. A PPI co-investigator will continue to contribute to the trial management group, including comments on patient facing materials and the dissemination plan.

Ethics and Dissemination

For peer review only - http://bmjopen.bmj.com/site/about/guidelines.xhtml

NHS research ethics approval was obtained from North East – Tyne & Wear South Research Ethics Committee, reference 18/NE/0300. The current protocol is version 4.0 (11/12/20019). A Trial Steering Committee is providing overall supervision and an Independent Data Safety and Monitoring Committee is responsible for reviewing and assessing recruitment, interim monitoring of safety and effectiveness, trial conduct and external data.

All safety events will be recorded by researchers when they are made aware of the event by the patient, carer, the treating clinicians, or therapists. Adverse event reports and serious adverse events (SAEs) not related to the intervention will be entered on to the remote data entry system. Each SAE will be assessed by the relevant PI to determine whether it is related to the intervention. A related SAE will be assessed by the CI to determine whether it is expected. If the SAE is related and unexpected (RUSAE) it will be reported to the Research Ethics Committee (REC) and sponsor in an expedited manner.

Reporting of the trial will be consistent with the CONSORT 2010 Statement (patient reported outcomes and non-pharmcological interventions) [53]. We will submit the final report to a peer-reviewed academic journal, according to our publication strategy and authorship policy. Research data will be available for secondary analysis upon reasonable request.

Trial Status

At the time of submission this trial had been open in nine sites and had recruited 96 patients and 10 carers, with a recruitment rate of two patient participants per site per month and a retention rate of 83%, which fulfilled the progression criteria of the internal pilot. However,

For peer review only - http://bmjopen.bmj.com/site/about/guidelines.xhtml

recruitment to the trial is currently suspended because of the COVID-19 pandemic. Wherever possible, participants already recruited into the trial will complete their follow-up assessments over the telephone or by post, extra rehabilitation sessions will be delivered over the telephone. When trial recruitment resumes, updated recruitment information will be found on the website http://femur3study.co.uk/

Abbreviations

AMTS	Abbreviated Mental Test Score
ANCOVA	Analysis of Covariance
CI	Chief Investigator
CSI	Carer Strain Index
CSRI	Client Service Receipt Inventory
EQ-5D-3L	EuroQol 5 Dimensions 3 Levels
FEMuR	Fracture in the Elderly Multidisciplinary Rehabilitation
FES-I	Falls Efficacy Scale - International
GP	General Practitioner
HADS	Hospital Anxiety and Depression Scale
ISRCTN	International Standard Randomised Controlled Trial Number
IV	Instrumental Variable
LCTC	Liverpool Clinical Trials Centre
NEADL	Nottingham Extended Activities of Daily Living
NHS	National Health Service
NICE	National Institute of Health and Clinical Excellence
PI	Principal Investigator

For peer review only - http://bmjopen.bmj.com/site/about/guidelines.xhtml

PPI	Patient and Public Involvement
QALY	Quality Adjusted Life Year
RCT	Randomised Controlled Trial
REC	Research Ethics Committee
RUSAE	Related Unexpected Serious Adverse Event
SAE	Serious Adverse Event
SPPB	Short Physical Performance Battery
VAS	Visual Analogue Scale
VASFoF	Visual Analogue Score - Fear of Falling

References

1. Royal College of Physicians, *National Hip Fracture Database annual report*. 2016: London.
2. Baker PN, Salar O, Ollivere BJ, Forward DP, Weerasuriya N, Moppett IK, et al., Evolution of the hip fracture population: time to consider the future? A retrospective observational analysis. *BMJ Open,* 2014. 4(4): p. e004405.
3. White SM and Griffiths R, Projected incidence of proximal femoral fracture in England: a report from the NHS Hip Fracture Anaesthesia Network (HIPFAN). *Injury,* 2011. 42(11): p. 1230-3.
4. Roche JJ, Wenn RT, Sahota O, and Moran CG, Effect of comorbidities and postoperative complications on mortality after hip fracture in elderly people: prospective observational cohort trial. *BMJ,* 2005. 331(7529): p. 1374.

For peer review only - http://bmjopen.bmj.com/site/about/guidelines.xhtml

5. Schnell S, Friedman SM, Mendelson DA, Bingham KW, and Kates SL, The 1-year mortality of patients treated in a hip fracture program for elders. *Geriatr Orthop Surg Rehabil,* 2010. 1(1): p. 6-14.

6. Bertram M, Norman R, Kemp L, and Vos T, Review of the long-term disability associated with hip fractures. *Injury Prev.,* 2011. 17(6): p. 365-70.

7. *National Osteoporosis Society. Key facts and figures. Accessed September 2014. Available at http://www.nos.org.uk/page.aspx?pid=328.*

8. Tian Y, Thompson J, Buck D, Sonola L. Exploring the system-wide costs of falls in older people in Torbay. London: The King's Fund. 2013.

9. Schnell S, Friedman SM, Mendelson DA, et al. The 1-year mortality of patients treated in a hip fracture program for elders. Geriatr Orthop Surg Rehabil 2010;1:6–14.

10. National Clinical Guideline Centre. Hip fracture: the management of hip fracture in adults| Guidance and guidelines | NICE. 2011 http://www. nice. org. uk/ guidance/ cg124 (accessed 2020).

11. Royal College of Physicians' Falls and Fragility Fracture Audit Programme. Recovering after a hip fracture: helping people understand physiotherapy in the NHS. Physiotherapy 'Hip Sprint' audit report 2017. https://www.fffap.org.uk/FFFAP/landing.nsf/PHFSA201802w1.pdf (accessed 2020).

12. Handoll HHG, Cameron ID, Mak JCS, et al. Multidisciplinary rehabilitation for older people with hip fractures. Cochrane Database Syst Rev 2009;15.

13. Handoll HH, Sherrington C, Mak JC. Interventions for improving mobility after hip fracture surgery in adults. Cochrane Database Syst Rev 2011:CD001704.

For peer review only

14. Crotty M, Unroe K, Cameron ID, et al. Rehabilitation interventions for improving physical and psychosocial functioning after hip fracture in older people. Cochrane Database Syst Rev 2010:CD007624.

15. Smith TO, Smith TO, Gilbert AW, Sreekanta A, Sahota O, Griffin XL, Cross JL, Fox C, Lamb SE. Enhanced rehabilitation and care models for adults with dementia following hip fracture surgery. Cochrane Database Syst Rev 2020;(2): CD010569

16. Chudyk A, Jutai J, Petrella R, Speechley M. Systematic review of hip fracture rehabilitation practices in the elderly. Archives of Physical Medicine and Rehabilitation 2009;90:246-62.

17. Auais MA, Eilayyan O, Mayo NE. Extended Exercise Rehabilitation After Hip Fracture Improves Patients' Physical Function: A Systematic Review and Meta-Analysis. Physical Therapy 2012;92:1437-51.

18. Allen J, Koziak A, Buddingh S, Liang J, Buckingham J, Beaupre L. Rehabilitation in patients with dementia following hip fracture: A systematic review. Physiotherapy Canada 2012;64:190-201.

19. Williams NH, Roberts JL, Din NU, et al. Developing a multidisciplinary rehabilitation package following hip fracture and testing in a randomised feasibility study: Fracture in the Elderly Multidisciplinary Rehabilitation (FEMuR). Health Technol Assess 2017;21:1–528.

20. Medical Research Council. Developing and evaluating complex interventions: new guidance. London: Medical Research Council, 2008.

21. Roberts JL, Din NU, Williams M, et al. Development of an evidence-based complex intervention for community rehabilitation of patients with hip fracture using realist review, survey and focus groups. BMJ Open 2017;7:e014362.

For peer review only - http://bmjopen.bmj.com/site/about/guidelines.xhtml

22. Williams NH, Hawkes C, Din NU, et al. Fracture in the Elderly Multidisciplinary Rehabilitation (FEMuR): study protocol for a phase II randomised feasibility study of a multidisciplinary rehabilitation package following hip fracture [ISRCTN22464643]. Pilot Feasibility Stud 2015;1:13.

23. Williams NH, Roberts JL, Din NU, et al. Fracture in the Elderly Multidisciplinary Rehabilitation (FEMuR): a phase II randomised feasibility study of a multidisciplinary rehabilitation package following hip fracture. BMJ Open 2016;6:e012422.

24. Roberts JL, Pritchard AW, Williams M, Totton N, Morrison V, Din NU, Williams NH. Mixed methods process evaluation of an enhanced rehabilitation intervention for elderly hip fracture patients. BMJ Open 2018;8:e021486.

25. Bandura A. Self-efficacy: Toward a unifying theory of behavioral change. *Psychol Rev* 1977; 84: 191–215.

26. Nouri F, Lincoln N. An extended activities of daily living scale for stroke patients. Clin Rehabil. 1987;1:301–5.

27. Wu CY, Chuang LL, Lin KC, et al. Responsiveness, minimal detectable change, and minimal clinically important difference of the Nottingham extended activities of daily living scale in patients with improved performance after stroke rehabilitation. Arch Phys Med Rehabil 2011;92:1281–7.

28. Zigmond A, Snaith R. The Hospital Anxiety and Depression Scale. Acta Psychiatr Scand. 983;67:1983.

29. EuroQol Group. EuroQol – a new facility for the measurements of health-related quality of life. Health Policy 1990;16:199-208.

For peer review only

For peer review only - http://bmjopen.bmj.com/site/about/guidelines.xhtml

30. Database of Instruments for Resource Use Measurement. Client Service Receipt Inventory (CSRI). http://www.dirum.org/instruments/details/44. Accessed 31 January 2019.

31. Brokelman RH, van Loon C, Hol A, van Kampen A, Veth R. The validation of the visual analogue scale for patient satisfaction after total hip arthroplasty. Eur Orthop Traumatol 2012;3:101–5.

32. Hauer K, Yardley L, Beyer N, Kempen G, Dias N, Campbell M, et al. Validation of the Falls Efficacy Scale and Falls Efficacy Scale International in geriatric patients with and without cognitive impairment: results of self-report and interview-based questionnaires. Gerontology. 2010;56:190–9.

33. Yardley L, Beyer N, Hauer K, Kempen G, Piot-Ziegler C, Todd C. Development and initial validation of the Falls Efficacy Scale-International (FES-I). Age Ageing. 2005;34:614–9.

34. Scheffer AC, Schuurmans MJ, van Dijk N, van der Hooft T, de Rooij SE. Reliability and validity of the visual analogue scale for fear of falling in older persons. J Am Geriatr Soc. 2010;58:2228–30.

35. Hodkinson H. Evaluation of a mental test score for assessment of mental impairment in the elderly. Age Aging. 1972;1:233–8.

36. Giampaoli S, Ferrucci L, Cecchi F, Lo Noce C, Poce A, Dima F, et al. Hand-grip strength predicts incident disability in non-disabled older men. Age Ageing.1999;28:283–8.

37. Rantanen et al., 'Midlife hand grip strength as a predictor of old age disability' JAMA 1999 281(6):558-560;

38. Sallinen et al., 'Hand-grip strength cut-points to screen older persons at risk for mobility limitation' 2010 J Am Geriatr Soc 58(9):1721-1726.

For peer review only

For peer review only - http://bmjopen.bmj.com/site/about/guidelines.xhtml

39. Guralnik JM, Simonsick EM, Ferrucci L, Glynn RL, Berkman LF, Blazer DG, et al. A short physical performance battery assessing lower extremity function: association with self-reported disability and prediction of mortality nursing home admission. J Gerentol 1994; 49(2):M85-M94.

40. Guralnik JM, Ferrucci L, Pieper CF, Leveille SG, Markides KS, Ostir GV, et al. Lower extremity function and subsequent disbility: consistency across studies, predictive modes, and value of gait speed alone compared with the short physical performance battery. J Gerentol A 2000; 55(4):M221-M231.

41. Robinson BC. Validation of a caregiver strain index. J Gerontol. 1983;38(3):344–8.

42. Parker S G, Oliver P, Pennington M, Bond J, Jagger C. Rehabilitation of older patients: day hospital compared to rehabilitation at home. A randomised controlled trial. Health Technol Assess 2009;13(39).

43. Department of Health. NHS reference costs 2015 to 2016. Accessed April 2020. https://www.gov.uk/government/publications/nhs-reference-costs-2015-to-2016

44. Curtis L, Burns A. Unit costs of health and social care 2019. Personal Social Services Research Unit (PSSRU). Accessed April 2020. https://www.pssru.ac.uk/project-pages/unit-costs/unit-costs-2019/

45. Glick H, Doshi J, Sonnad S, Polsky D. Economic evaluation in clinical trials. Oxford: Oxford University Press; 2007.

46. Drummond MF, Sculpher MJ, Torrance GW, O'Brien BJ, Stoddart GL. Methods for the economic evaluation of health care programmes. Oxford: Oxford University Press; 2005.

47. National Institute for Health and Care Excellence. Guide to the methods of technology appraisal. Accessed on 11 June 2014.

For peer review only

For peer review only - http://bmjopen.bmj.com/site/about/guidelines.xhtml

http://www.nice.org.uk/article/pmg9/resources/non-guidance-guide-to-themethods-of-technology-appraisal-2013-pdf. 2013.

48. Oakley, A., Strange V., Bonell C., Allen E. & Stephenson J. (2006). Process evaluation in randomised controlled trials of complex interventions. Health Services Research 2006; 332: 413-416.

49. Carroll C, Patterson M, Wood S, Booth A, Rick J, Balain S. A conceptual framework for implementation fidelity. Implementation Science 2007; 2: 40-49.

50. Grant A, Treweek S, Dreischulte T, Foy R, Guthrie B. Process evaluations for cluster-randomised trials of complex interventions: a proposed framework for design and reporting. Trials 2013; 14: 15-25.

51. Masterson-Algar P, Burton CR, Rycroft-Malone J, Sackley C, Walker M. Towards a programme theory for fidelity in the evaluation of complex interventions. Journal of Evaluation of Clinical Practice 2014; 20(4): 445-452.

52. Braun V, Clarke V. Using thematic analysis in psychology. Qualitative Research in Psychology 2006; 3: 77-101.

53. Schulz KF, Altman DG , Moher D , et al. CONSORT 2010 statement: updated guidelines for reporting parallel group randomised trials. BMC Med 2010;8:18

For peer review only

Author Statement

NHW was the chief investigator and grant holder, was responsible for study design, conduct and analysis and had overall responsibility for the study and acts as guarantor. LH, SH and CSo were the trial co-ordinators and BH was the senior trial co-ordinator overseeing day-to-day conduct, and provided methodological input, along with SDob who is an experienced

For peer review only - http://bmjopen.bmj.com/site/about/guidelines.xhtml

health services researcher. SDod and DC wrote the statistical analysis plan. RhTE and JC wrote the health economic analysis plan. PMA and PR wrote the process evaluation analysis plan with NHW. SDor is a Patient and Public Involvement co-applicant. RL is a lead applicant from Bangor University and oversees sites in North Wales. MB is a lead applicant from Cardiff University and oversees sites in South Wales. PL is a lead applicant from University of Nottingham and oversees sites in Nottingham. CSa is a lead applicant now in University of Nottingham, but previously in Kings College London, and oversees sites in Kent. TS is a lead applicant from the University of East Anglia and oversees the site in East Anglia. VM and ABL were co-investigators responsible for study design, methodological oversight and provided health psychology and exercise science expertise respectively. All authors were involved in drafting, revising and approving this manuscript.

Acknowledgements

The authors would like to thank all of the principal investigators, research nurses, hospital staff, community rehabilitation teams, physiotherapists, occupational therapists and their assistants who are providing the additional therapy sessions. In Nottingham: Miriam Golding-Day, Katie Robinson, Jane Horne, Frances Allen. In north Wales: Matthew Jones, Lauren Porter, Jane Stockport, Mary Roberts, Victoria Whitehead, Yogesh Joshi, Gareth Evans, Cathy Baker, Jeannie Bishop, Jane Heron, Llinos Davies. In south Wales: Rachel Lowe, Rebecca Milton, Andrew Dean Young, Peter Lewis, Lisa Roche. In East Anglia: Stephanie Tuck, Catherine Mingay, Stephanie Howard. In east Kent: Danielle Pearce, Joanne Jackson, Sarah Chapman, Andrew Smith, Alex Chipperfield, Joanne Deery.

For peer review only - http://bmjopen.bmj.com/site/about/guidelines.xhtml

For peer review only

Special thanks to the LCTC data management team Clare Jackson and Dianne Wheatley, LCTC information systems team Keith Kennedy and Janet Harrison, trial co-ordinator assistant Kieran Crabtree and quality assurance manager Katie Neville. Research Assistants/Research Project Support Officers who perform follow-up visits. Others who have contributed to the trial management group Kevin Anthony, Gail Arnold, Janine Bates, Suzannah Evans, Mark Howells, Lara Lavelle-Langham, Nic Nikolic, Jessica Roberts. Also the North West Coast Clinical research Network Lisa Cheng, Julia Murgarza.

We very much appreciate the work of the Trial Steering Committee Gail Mountain (chair), Graeme Holt, Helen Hughes, George Kernohan, Tosan Okoro, Nikki Totton, and the Independent Data and Safety Monitoring Committee Chris Robertson (chair), Diane Dixon, Peter Giannoudis, Barbara Hanratty.

Funding

This work was supported by the National Institute for Health Research's Health Technology Assessment Programme, grant number 16/167/09. The views and opinions expressed therein are those of the authors and do not necessarily reflect those of the HTA, NIHR, NHS or the Department of Health.

Trial Sponsor

University of Liverpool: sponsor's reference: UoL001378. Contact: Mr Alex Astor, University of Liverpool, 2nd Floor Block D Waterhouse Building, 3 Brownlow Street, Liverpool L69 3GL. The university has appropriate clinical trials and professional indemnity insurance.

Conflicts of Interest

For peer review only - http://bmjopen.bmj.com/site/about/guidelines.xhtml

For peer review only

NHW reports additional grants from NIHR HS&DR outside the submitted work and membership of the NIHR HTA programme funding committee (commissioned research).

Data Statement

Technical appendix, statistical code, and dataset will be made available.

For peer review only

For peer review only - http://bmjopen.bmj.com/site/about/guidelines.xhtml

Table 1 Outcome measures

Patient Completed Measures - Primary	Description	Range
Nottingham Extended Activities of Daily Living (NEADL) scale [26, 27]	Activities of daily living (mobility, kitchen, domestic, leisure) with higher score indicating greater independence	(0-66)
**Patient Completed Measures - Secondary**		
Hospital Anxiety and Depression Scale (HADS) [28]	Anxiety and depression in patients with physical health problems. Two sub-scales (0-21) with higher score indicates greater anxiety or depression	(0-21)
**Patient Completed Economic Measures**		
EuroQol EQ-5D-3L [29]	Health utility index with five dimensions (mobility, self-care, usual activities, pain/discomfort, anxiety/depression) and three levels to give health states converted to a utility weight. Also Visual Analogue Score (VAS) for health state today	Health utility weight from 0 (death) to 1.0 (perfect health) also with negative values VAS (0-100)
Client Service Receipt Inventory (CSRI) [30]	Use of health and social care services	According to activity
**Patient Completed Process Measures (potential mediators of outcomes)**		
Visual Analogue Scale (VAS) for hip pain intensity [31]	VAS of current hip pain intensity	(0-10cm)
Falls Efficacy Scale - International (FES-I) (self-efficacy) [32,33]	How concerned a patient is about falling when performing 16 activities of daily living both inside and outside of the home, rated from 1 (not at all concerned) to 4 (very concerned)	(16-64)
Visual Analogue Score - Fear of Falling (VASFoF) [34]	VAS with higher scores indicating greater fear of falling	(0-10cm)
**Assessment of cognitive function**		

For peer review only

Abbreviated Mental Test Score (AMTS) [35, 36]	Detecting and monitoring cognitive impairment. 10 items with lower scores indicating worse cognitive function	(0-10)
**Objective measures of physical function**		
Grip strength [37]	Hand dynamometer	According to meter reading
Short Physical Performance Battery (SPPB) [40,41]	Physical function tests assessing lower limb function in terms of balance, gait, strength and endurance. Higher score indicates greater function	(0-12)
**Carer completed measure - secondary outcome**		
Caregiver Strain Index (CSI) [42]	13-items in the domains: employment, financial, physical, social and time. Positive responses to seven or more items indicate a greater level of strain	(0-13)
Hospital Anxiety and Depression Scale (HADS) [28]	Anxiety and depression in carers. Two sub-scales (0-21) with higher score indicates greater anxiety or depression	(0-21)

For peer review only

For peer review only - http://bmjopen.bmj.com/site/about/guidelines.xhtml

Table 2 FEMuR III protocol schedule of forms and procedures

Participant follow-up visits should take place at 17 (+/- 2 weeks) and 52 (+/- 2 weeks) weeks post randomisation.

Procedures	Screening	Baseline / Randomisati	Trial intervention	17 weeks post randomisati	Qualitative interviews	52weeks post randomisati
**Eligibility screening and consent**						
Assessment of eligibility criteria	X					
Written and informed consent (patient / carer))	X					
Confirm consent		X	X	X	X	X
Randomisation		X				
Discharge data		X				
**Outcome measurement - patient**						
NEADL		X		X		X
HADS		X		X		X
AMTS		X		X		X
VAS hip pain intensity		X		X		X
FES-I		X		X		X
VASFoF		X		X		X
EQ-5D-3L		X		X		X
CSRI		X		X		X
Grip strength		X		X		X
SPPB				X		X
**Outcome measurement - carer**						
CSI		X		X		X
HADS		X		X		X
**Trial Intervention****			X			
**Qualitative interviews**						
Re-affirm consent verbally specifically for qualitative phone interview. (patient / carer)					X	

For peer review only - http://bmjopen.bmj.com/site/about/guidelines.xhtml

Procedures	Screening	Baseline / Randomisati	Trial intervention	17 weeks post randomisati	Qualitative interviews	52weeks post randomisati
Qualitative telephone interview					X	
**Safety Event Reporting**						
Monitoring of Adverse Events			X	X	X	X
Monitoring of Serious Adverse Events			X	X	X	X

* Randomisation and baseline should take place no later than 6 weeks after hip fracture repair surgery

** If randomised to intervention arm.

For peer review only

For peer review only - http://bmjopen.bmj.com/site/about/guidelines.xhtml

Figure Legend

Figure 1 **Participant Flowchart for FEMuR III**

For peer review only

For peer review only - http://bmjopen.bmj.com/site/about/guidelines.xhtml

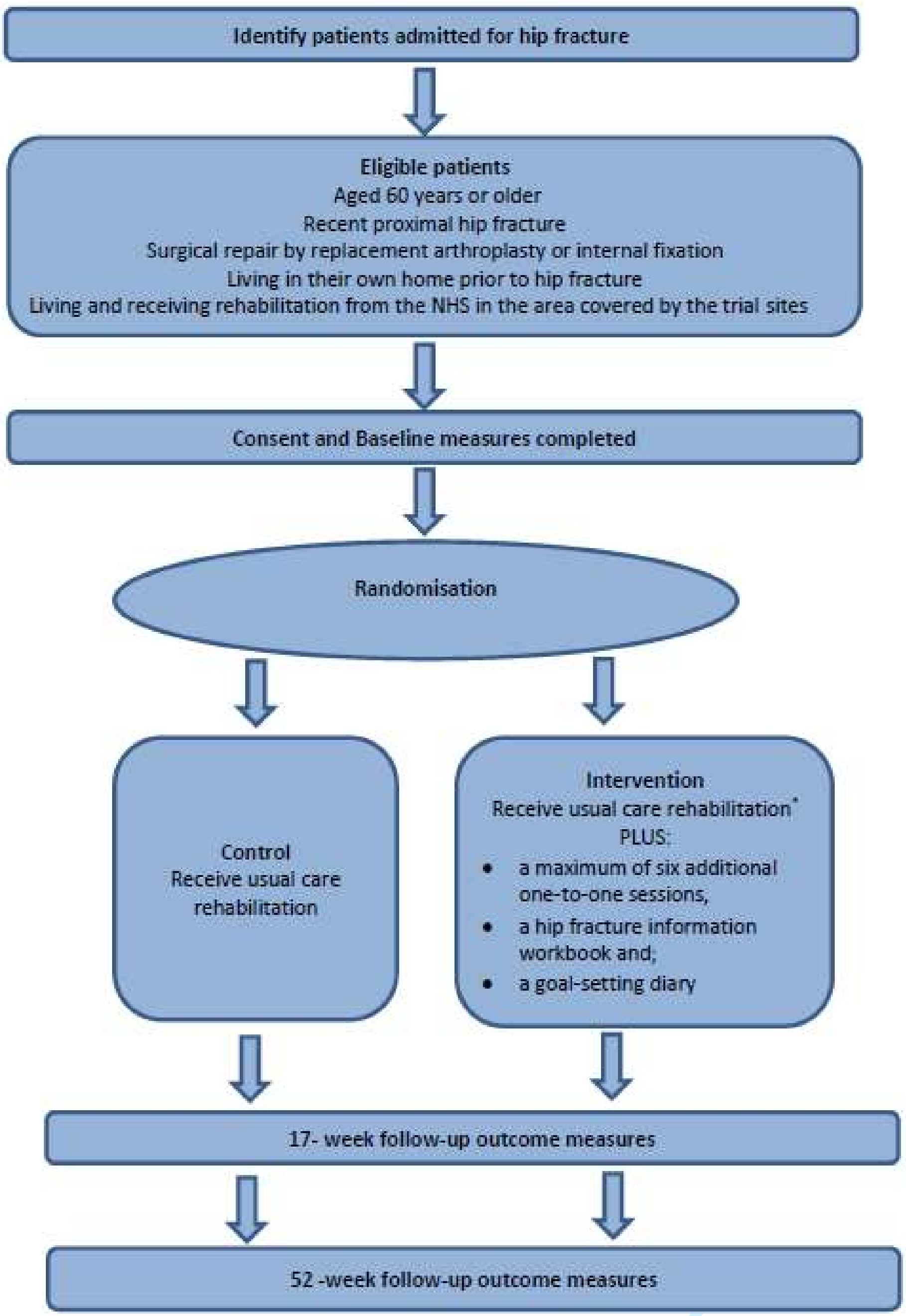
Identify patients admitted for hip fracture
Eligible patients
Aged 60 years or older
Recent proximal hip fracture
Surgical repair by replacement arthroplasty or internal fixation
Living in their own home prior to hip fracture
Living and receiving rehabilitation from the NHS in the area covered by the trial sites
Consent and Baseline measures completed
Randomisation
Control
Receive usual care rehabilitation
Intervention
Receive usual care rehabilitation*
PLUS:
a maximum of six additional one-to-one sessions,
a hip fracture information workbook and;
a goal-setting diary
17- week follow-up outcome measures
52 -week follow-up outcome measures

Appendix 1 FEMuR III Trial Registration Data

Data category	Information
Registry and trial identification no.	ISRCTN28376407
Date of registration	23/11/2018
Funder	NIHR Evaluation, Trials and Studies Co-ordinating Centre (NETSCC); Grant code 16/167/09
Sponsor	University of Liverpool
Contact for public enquiries	LH email: femur3@liverpool.ac.uk
Scientific title	Fracture in the Elderly Multidisciplinary Rehabilitation - Phase III (FEMuR III): a definitive randomised controlled trial and economic evaluation of a community-based rehabilitation package following hip fracture
Acronym	FEMuR III
Countries of recruitment	United Kingdom
Health condition	Hip fracture
Intervention	Intervention comparator: Enhanced rehabilitation
	Control comparator: Usual care
Inclusion criteria	Aged 60 years or older
	Recent proximal hip fracture
	Surgical repair by replacement arthroplasty or internal fixation
	Living in own home prior to hip fracture
	Living and receiving rehabilitation from the NHS in the area covered by the trial centres
Exclusion criteria	Living in residential or nursing home prior to hip fracture
	Unable to understand English or Welsh
	Lacking mental capacity to give informed consent
Study design	Interventional
	Randomised controlled trial
	Treatment, education or self-management, psychological and behavioural, complex intervention, physical, rehabilitation
Recruitment start date	01/04/2019
Target sample size	446
Primary outcome	Nottingham Extended Activities of Daily Living scale
Secondary outcomes	EuroQol EQ-5D, Hospital Anxiety and Depression Scale, Abbreviated Mental Test Score, Falls Efficacy Scale – International, hip pain intensity, fear of falling, grip strength, short physical performance battery

For peer review only

Appendix 2 FEMuR III Patient Participant Information Sheet and Informed Consent Forms

<<Local NHS Logo to go here>>

Fracture in the Elderly Multidisciplinary Rehabilitation III

<Trust/Site address 1>
<Trust/Site address 2>
<Trust/Site address 3>
<postcode>
Tel: <telephone number>

Patient Participant Information Sheet

Contents

Contents	Page
You are invited to take part in FEMuR III	1
Why are we doing the FEMuR III study?	2
What is the Enhanced Rehabilitation Package?	2
Why have I been asked to take part?	2
What will I have to do if I take part?	2
Timeline of visits	3
How will I know which treatment I'm going to have?	3
What are the benefits and risks of taking part?	3
What are the alternatives for treatment?	3
Do I have to take part?	4
What happens if I change my mind?	4
Will my participation be kept confidential?	4
What will happen to the results of the study?	5
What if there is a problem?	5
Additional information	5
Additional information about future research	5
Contacts for further information	6
Consent Form	7

Important Contact Information

Thank you for taking the time to read this information sheet. We hope you will find this information helpful.

If you would like a large print version of this information sheet please ask your research team.

If you have any questions about this study please talk to your research team:

<Add contact details for PI/RN i.e., name and telephone number>

Website: Femur3study.co.uk

You are invited to take part in FEMuR III

Important things to know about FEMUR III:

- FEMuR III aims to compare a new enhanced rehabilitation package with standard NHS care for patients who have had surgery to repair a hip fracture.
- We are interested in the recovery of patients aged over 60 years old who lived independently before they suffered a hip fracture even if they were in receipt of personal care at home.
- Being part of the study means you will receive either standard care or standard care **plus** enhanced rehabilitation when you leave hospital.
- Standard care can vary but usually involves community-based physiotherapy. The enhanced rehabilitation package will provide additional physiotherapy, occupational therapy and some 'self-help' tools to aid recovery.
- You have been given this information sheet as you might be eligible to take part in this study.
- Before you decide whether or not to take part it is important for you to understand why the research is being done and what it will involve for you. Please take time to read the following information carefully and discuss it with friends or relatives if you wish.
- Taking part is voluntary. If you don't want to take part then you don't need to give a reason.
- If you have someone who provides you with help for most days of the week with activities of daily living or physical care, we would also like them to be involved in the study.
- Please ask a member of your clinical team if there is anything that is not clear, or if you would like more information.

Why are we doing the FEMuR III study?

Hip fracture is a common, major health problem in old age, especially for people with other health problems or who are frail. Some patients who suffer this type of fracture need surgery to repair it. They take a long time to recover, and others may not recover fully.

Once patients are discharged, the routine care they receive can vary depending on local NHS policy. Some may not find it as easy to live independently afterwards.

We have designed an enhanced rehabilitation package for patients who are recovering from this surgery, which is delivered in addition to standard NHS care. FEMuR III will compare the enhanced package with standard NHS care to see if it can improve recovery for patients.

What is the Enhanced Rehabilitation Package?

The enhanced rehabilitation package is made specifically for each patient and we think this should improve recovery. We think this package should work better if it includes physiotherapy (to help patients recover movement), occupational therapy (to help patients with activities associated with daily living) and also provides tools to help build confidence and mood.

The enhanced rehabilitation package we have designed involves additional rehabilitation at follow up visits. You will be given a workbook and a goal-setting diary to complete during the first few months of recovery.

In order to compare the enhanced rehabilitation package to standard NHS care we are asking 446 people to take part in our study. We will follow your progress in the 12 months after surgery and collect information from you during that time so we can see how you are. The information we collect will help us to see whether there is a difference between those people who have standard NHS treatment and those who receive standard NHS treatment **and** the enhanced rehabilitation package.

Why have I been asked to take part?

We are inviting you to take part in this study because you are a patient at one of the hospitals taking part and have recently had surgery to repair a hip fracture.

What will I have to do if I take part?

A member of the clinical team can talk to you in more detail about this study and you will be able to ask any questions that you have. If you have had all of your questions answered and are happy to take part then you will be asked to sign a consent form to confirm you want to take part. You will be given a copy of your consent form and this information sheet to keep.

This study is comparing standard NHS care with the enhanced rehabilitation package. Both of these will be tailored to individual patients so it is difficult for us to describe exactly what your rehabilitation will look like. However, the main differences between the two are that if you are receiving the enhanced rehabilitation package you will also:

- Be given a goal-setting diary to complete which we would like you to use to set yourself targets and track your progress through your rehabilitation.
- Be given a workbook containing information about hip fractures: what to expect during recovery, tips to aid recovery, and useful contacts if you would like more information.
- Receive up to six community/home-based therapy sessions **in addition** to any provided by the NHS.

If you do decide to take part and have given your consent, we will ask you to complete some questionnaires and do a grip-strength test so we can get some information about how you are feeling both mentally and physically. This will allow us to see how you are recovering over time. The grip – strength test uses a special piece of equipment to measure this and will be carried out at home with a researcher.

We will arrange an appointment with you around 4 months after you have started the study to see how you are getting on. This visit may take place at your home or in a community hospital depending on what is best with you. At the visit, we will ask you to complete the same questionnaires and do some basic physical tests so we can see how you are recovering. At around 12 months after you started the study we will arrange one more visit to complete the questionnaires and physical tests again so we can track your progress. The physical tests are things you do every day. For example, the researcher will ask you to sit and stand up to five times (if you are able) and will record your progress.

For peer review only - http://bmjopen.bmj.com/site/about/guidelines.xhtml

We may also telephone you soon after the 4-month visit to ask some questions about how you are finding being involved in the study. This will give you a chance to give some feedback on your experiences and your views on the care you are receiving. Not all patients will receive a phone call, we aim to call 60 patients who have agreed to take part. We will offer you a £30 shopping voucher for your time and inconvenience in taking part in the telephone interview.

Timeline of visits

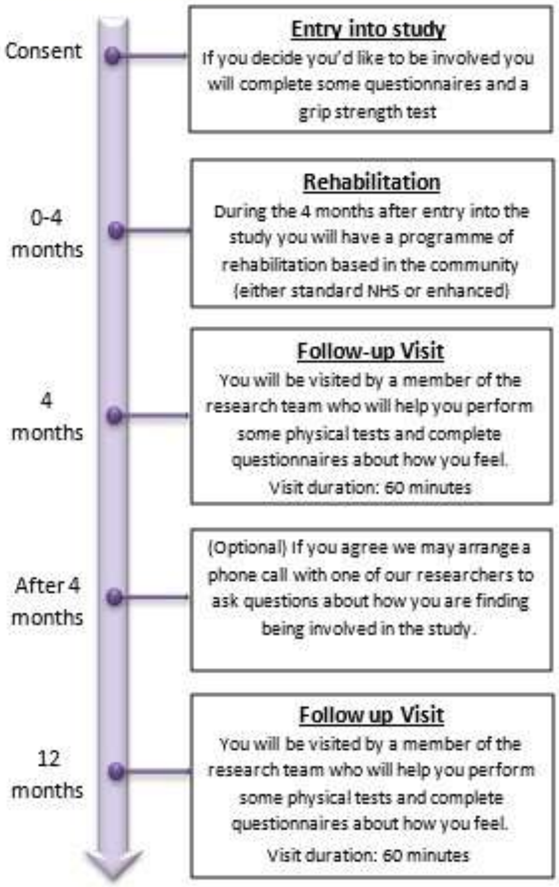

How will I know which treatment I'm going to have?

In the FEMuR III study patients will be split into two groups at random:

- One group will receive standard NHS care after discharge
- The other group will receive the enhanced rehabilitation package alongside standard NHS care after discharge

We use a computer programme that puts patients 'at random' into one of the groups – you might hear this described as 'randomisation' or 'random allocation', but they all mean the same thing. Neither you nor your doctor chooses which group you are in.

In this study you are equally likely to be in the group receiving standard NHS care as you are in the group receiving the enhanced package. Your healthcare team will let you know which group you are in as soon as possible.

What are the benefits and risks of taking part?

We are not sure whether standard NHS care or the enhanced rehabilitation package is best, but we anticipate that both will aid your recovery following surgery.

We do not foresee any significant risks involved in taking part in FEMuR III. All of the physical exercises suggested are used in normal rehabilitation after hip fracture and will be supervised by trained healthcare professionals to minimise any risk. The enhanced rehabilitation package will take up more of your time due to additional therapy sessions and having to complete the diary.

We hope that the results from the study will help doctors, therapists, patients and their carers in the future when making decisions about treatment.

What are the alternatives for treatment?

Patients recovering from hip fracture repair will get standard NHS treatment, though this may vary in different areas. In this study every patient will get standard NHS treatment even if they are in the enhanced rehabilitation group. Currently, there are no other alternative treatment programmes available.

Do I have to take part?

No, taking part is voluntary. It is up to you to decide whether or not you want to take part. If you choose to take part you can also choose to stop at any time without giving a reason. The standard of care you receive now or in the future will be the same whether you take part or not.

For peer review only - http://bmjopen.bmj.com/site/about/guidelines.xhtml

What happens if I change my mind?

If at any point you decide to stop taking part in the study you will receive the treatment and follow up usually offered in your area. The standard of care you receive will remain the same if you decide to stop taking part. If you do decide to stop taking part we will ask you if you would like to either:

- continue to complete follow up visits for the study
- stop taking part with no more study visits.

We will use any study information collected up until the time you stop taking part.

Will my participation be kept confidential?

Yes. All information collected about you during the course of the study will be handled according to relevant ethical and legal requirements. Your personal information will be kept strictly confidential and will only be accessed by people working on the study, or working to ensure the study is being run correctly.

You will be given a study number, which will be used along with your initials to identify you on each paper form. Your full name and date of birth, postcode, contact details and NHS number will be included on your consent form and a copy of this will be sent to the study team at the coordinating centre for the study, the Liverpool Clinical Research Centre (LCTC.) There may be instances (depending on your local NHS,) when a copy of your contact details will need to be sent to other locations or Universities within your local area to arrange your follow up visits. Only members of the FEMUR III team will be given access to your contact details, they will be held securely and destroyed after your final follow-up. Every effort will be made to ensure that any further information about you that leaves the hospital will have your name removed so that you cannot be recognised from it. This information will usually be removed by a member of the study team at your hospital/community care, but may also be removed by study team members at the LCTC upon receipt. We will also ask for your telephone number so that we can contact you for the telephone interviews during the study. Your telephone number will not be used for anything else without your consent.

With your consent, we will send a letter to your GP to let them know you are taking part and we will use your NHS number and postcode to access data about your use of health services (for example your hospital admissions).

The University of Liverpool is the sponsor for this study based in the United Kingdom. The University of Liverpool along with Bangor University will be using information from you and your medical records in order to undertake this study and will act as joint data controllers for this study. This means that both joint data controllers are responsible for looking after your information and using it properly. The University of Liverpool will keep identifiable information about you for a maximum of 25 years after the study has finished. Arrangements for confidential destruction will then be made.

Details about the use of health services (health economics) will be collected in this study. Information will be obtained from your hospital finance department and NHS Digital. Health economics researchers at the Centre for Health Economics and Medicines Evaluation (part of Bangor University), who are part of the study team, will use these data to calculate the overall costs of care. Data will be provided to the study team by NHS Digital and in order to obtain this, your NHS number will be securely transferred to NHS Digital by the CTRC using an encrypted electronic transfer system.

We would also like to collect information regarding your therapy sessions from electronic data from Therapy Management systems. This data will include date of sessions, location of sessions and activities completed during the session. This data will help us to see how many therapy sessions you have completed during the study. This data will be extracted from Therapy Management systems by NHS IT personnel and transferred using an encrypted electronic transfer system to a researcher at the University of Liverpool.

Your rights to access, change or move your information are limited, as we need to manage your information in specific ways in order for the research to be reliable and accurate. If you withdraw from the study, we will keep the information about you that we have already obtained. To safeguard your rights, we will use the minimum personally-identifiable information possible.

You can find out more about how we use your information in the "**How we use your information**" section on the study website *Femur3study.co.uk*

For peer review only - http://bmjopen.bmj.com/site/about/guidelines.xhtml

Your NHS hospital and/or community health team will collect information from you and your medical records for this research study in accordance with our instructions.

Your NHS hospital and/or community health team will use your name, NHS number and contact details to contact you about the research study, and make sure that relevant information about the study is recorded for your care, and to oversee the quality of the study. Individuals from the team and regulatory organisations may look at your medical and research records to check the accuracy of the research study. Your NHS hospital and/or community health team will pass these details to the University of Liverpool along with the information collected from you and your medical records. The only people in the University of Liverpool who will have access to information that identifies you will be people who need to confirm your participation in the study, to contact you after 4 months to ask you questions about taking part in this study or audit the data collection process. The people who analyse the information will not attempt to identify you or find out your name, NHS number or contact details.

Your NHS hospital and/or community health team will keep identifiable information about you from this study for up to a maximum of 25 years after the study has finished.

Additional information about future research:

When you agree to take part in a research study, the information about your health and care may be provided to researchers running other research studies in this organisation and in other organisations. These organisations may be universities, NHS organisations or companies involved in health and care research in this country or abroad. Your information will only be used by organisations and researchers to conduct research in accordance with the UK Policy Framework for Health and Social Care Research.

This information will not identify you and will not be combined with other information in a way that could identify you. The information will only be used for the purpose of health and care research, and cannot be used to contact you or to affect your care. It will not be used to make decisions about future services available to you, such as insurance.

What will happen to the results of the study?

We want the results of the study to be presented at conferences and published in medical journals so that we can explain to the medical, nursing and therapies' community what our research results have shown. You will not be identified in any publication or presentations.

What if there is a problem?

If you have a concern about any aspect of this study, you should ask to speak with one of your research team who will do their best to answer your questions.

If at any time during the study you feel distressed or anxious please speak to your research team, a therapist or contact your GP.

If you wish to make a formal complaint, you can do this through the NHS Complaints Procedure. Details can be obtained from your hospital.

Every care will be taken in the course of this clinical study. However, in the unlikely event that you are harmed by taking part in this research project, compensation may be available and you may have to pay your related legal costs. The University of Liverpool holds insurance against claims from participants for harm caused by their participation in this clinical trial. Participants may be able to claim compensation if they can prove that the University of Liverpool has been negligent. However, the NHS organisation that has provided your treatment has a duty of care to you, whether or not you agree to participate in the study, and the study sponsor accepts no liability for negligence on the part of your NHS organisation's employees. However, if you are harmed, and this is due to someone's negligence in the NHS, then you may have grounds for a legal action for compensation against the NHS organisation providing your treatment, but you may have to pay for your legal costs. The normal NHS complaints procedures should be available to you.

Additional information

The University of Liverpool is responsible for managing this study; they have asked that the day to day running of the study is carried out by the Liverpool Clinical Research Centre (LCTC,) part of the University of Liverpool. Additional support is provided by health economics researchers from the Centre for Health

For peer review only - http://bmjopen.bmj.com/site/about/guidelines.xhtml

Economics and Medicines Evaluation, part of Bangor University, and other researchers from participating universities (the study team).

This study is funded by the National Institute for Health Research's Health Technology Assessment Programme (ref: 16/167/09).

The study has been reviewed by the National Institute for Health Research (NIHR), Health Research Authority and the National Research Ethics Service Committee. Tyne and Wear South Ethics Committee has reviewed the study and given approval for it to take place.

The views expressed are those of the author(s) and not necessarily those of the NHS, the NIHR or the Department of Health.

Thank you for reading this information sheet.

Contacts for further information

If you would like more information or have any questions about the FEMuR III study please talk to:

Principal Investigator: <PI name to go here>

Research Nurse: <RN name to go here>

Telephone: <Hospital contact number to go here>

Or visit the website: Femur3study.co.uk

If you wish to discuss the study with someone independent of the research team you can contact the local NHS Patient Advice and Liaison Service (PALS) or local equivalent on: <Local PALS or equivalent telephone number to go here>

<<Local NHS Logo to go here>>

Fracture in the Elderly Multidisciplinary Rehabilitation III

<Trust/Site address 1>
<Trust/Site address 2>
<Trust/Site address 3>
<postcode>
Tel: <telephone number>

Patient Participant Consent Form

To be completed by the Researcher:			
Site Name:			
Participant Study Number			
Patient Initials:		Participant DOB:	/ /

Once you have read and understood each statement please enter your initials in each box		Initial
Example: I confirm that I have read and understand the Participant Information Sheet.		JS
1. I have read and understood the information sheet for this study. I have had the opportunity to ask questions and have had these answered satisfactorily.		
2. I understand that participation is voluntary and that I am free to withdraw from the study at any time, without giving a reason, and without my care or legal rights being affected. I understand that in some cases further information about any unwanted effects of treatment may need to be collected by the study team.		
3. I understand that my data will be retained for a maximum of 25 years at site and by the Liverpool Clinical Trials Centre (LCTC,) part of the University of Liverpool and that they will be stored in a confidential manner.		
4. I give permission for a copy of my consent form which will include my name and date of birth, postcode and NHS number to be sent to the members of the FEMUR III research team (where it will be kept in a secure location), to allow confirmation that my consent was given.		
5. I understand that relevant sections of my medical notes and any data collected during the study may be looked at by authorised individuals from the study team and those listed under "Additional information" (NHS organisation, sponsor and regulatory authorities). I give permission for these individuals to have access to my records.		
6. I agree to allow information or results arising from this study to be used in future healthcare and/or medical research in a pseudo-anonymised form.		
7. I agree to my GP being informed of my participation in the study.		
8. I agree for my data on NHS hospital admissions and treatment to be collected for the purpose of this study and understand this will include both routine paper and electronic NHS health care records covering the study period for health economic analysis.		
9. I agree for my routine electronic data from Therapy Management systems to be collected for the purpose of this study and understand that this will include both routine paper and electronic NHS health care records covering the study period for qualitative research purposes.		
10. I agree to take part in the above study.		
**The statements below are OPTIONAL** ***(you can still participate even if you only agree to the statements above):***		
11. I agree that I may be contacted in the future in relation to this or other related studies.		

For peer review only - http://bmjopen.bmj.com/site/about/guidelines.xhtml

12. I agree to being contacted by a study researcher to conduct a qualitative interview and for the interview to be recorded.<br>(if you agree to this statement provide your details below):<br>Telephone number:		☐

Your **full** name (please print):			
Your signature:		Date:	
To be completed by a witness only if participant is unable to sign the consent form:			
Witness **full** name (please print):			
Witness signature:		Date:	

To be completed by the Researcher (on the same day after participant has completed the form):			
Researcher **full** name (please print):			
Researcher signature:		Date:	
Additional details required for health economic analysis:			
Participant postcode:			
Participant NHS Number:			

For peer review only - http://bmjopen.bmj.com/site/about/guidelines.xhtml

Appendix 3 FEMuR III Carer Participant Information Sheet and Informed Consent Forms

Fracture in the Elderly Multidisciplinary Rehabilitation III

<<Local NHS Logo to go here>>

<Trust/Site address 1>
<Trust/Site address 2>
<Trust/Site address 3>
<postcode>
Tel: <telephone number>

Carer Participant Information Sheet

Contents

Contents	Page
You are invited to take part in FEMuR III	1
Why are we doing the FEMuR III study?	2
What is the Enhanced Rehabilitation Package?	2
Why have I been asked to take part?	2
What will I have to do if I take part?	2
Timeline of visits	3
How will I know which treatment I'm going to have?	3
What are the benefits and risks of taking part?	3
What are the alternatives for treatment?	3
Do I have to take part?	4
What happens if I change my mind?	4
Will my participation be kept confidential?	4
What will happen to the results of the study?	5
What if there is a problem?	5
Additional information	5
Contacts for further information	6
Consent Form	7

Important Contact Information

Thank you for taking the time to read this information sheet. We hope you will find this information helpful.

If you have any questions about this study please talk to your research team:

If you would like a large print version of this information sheet please ask your research team.

<Add contact details for PI/RN i.e., name and telephone number>

Website: Femur3study.co.uk

You are invited to take part in FEMuR III

Important things to know about FEMUR III:

- FEMuR III aims to compare a new enhanced rehabilitation package with standard NHS care for patients who have had surgery to repair a hip fracture.
- After surgery, some patients take a long time to recover, and others may not recover fully.
- Additional care is often provided by members of the family or close friends, and usually involves helping hip fracture patients with activities associated with daily living or physical care.
- You have been given this information sheet as you are a carer of someone who is potentially eligible to take part.
- Before you decide whether or not to take part it is important for you to understand why the research is being done and what it will involve for you. Please take time to read the following information carefully and discuss it with friends or relatives if you wish.
- Taking part is voluntary. If you don't want to take part then you don't need to give a reason.
- Please ask a member of the clinical team if there is anything that is not clear, or if you would like more information.

Why are we doing the FEMuR III study?

The FEMuR III study hopes to collect information to help provide evidence about the best way to treat patients who are recovering from hip repair surgery after a fracture. Hip fractures are common and often need to be surgically repaired. Once patients are discharged, the routine care they receive can vary depending on local

For peer review only - http://bmjopen.bmj.com/site/about/guidelines.xhtml

NHS policy and they often require additional help at home from family members or friends.

FEMuR III will compare an enhanced rehabilitation programme we have designed against the normal care given by the NHS. We want to see if one offers any additional benefit over the other. To do this, we are asking 446 patients and their carers to take part. We will follow progress in the 12 months after surgery and collect information from you during that time so we can see how you both are.

What is the Enhanced Rehabilitation Package?

The enhanced rehabilitation package is made specifically for each patient and we think this should improve recovery. The enhanced rehabilitation package mixes extra therapy with self-help tools which aim to help patients improve aspects of their own recovery e.g., build confidence in trying exercises by themselves. We also hope that this will help the people caring for hip fracture patients by reducing the level of care they need to provide as patients may be able to recover independence quicker.

The enhanced rehabilitation package we have designed involves additional rehabilitation sessions and patients will be given a workbook and a goal-setting diary to complete during the first few months of recovery. Carers will be given questionnaires to complete so we can look at how much help you are providing and how you are managing.

In order to compare the enhanced rehabilitation package with standard NHS care we are asking 446 people aged 60 and over, who have had hip repair surgery, to take part in our study. We will follow their progress in the 12 months after surgery and collect information from them and from you (as their carer) during that time so we can see how you both are. The information we collect will help us to see whether there is a difference between those people who have standard NHS treatment and those who receive standard NHS treatment and the enhanced rehabilitation package.

Why have I been asked to take part?

We are inviting you to take part in this study because you are a carer of a patient who has recently had surgery to repair a hip fracture at one of the hospitals taking part

What will I have to do if I take part?

A member of the clinical team will talk to you first in more detail and you will be able to ask any questions that you have. If you have had all of your questions answered and are happy to take part then you will be asked to sign a consent form to confirm you want to participate. You will be given a copy of the consent form and the information sheet to keep.

If you do decide to take part and have given your consent, we will ask you to complete some questionnaires so we can see how you are feeling. These may take around 15 minutes to complete.

We will arrange an appointment with your friend/relative around 4 months after surgery to see how they are getting on. This will involve a member of the research team coming out to visit your friend/relative and we would like to ask that you be present too. At the visit, we will ask you to complete the same questionnaires that you completed at the start, so that we can see if there is any difference in how you are feeling. At around 12 months after the surgery we will arrange one more visit to complete the questionnaires again so that we can see if anything has changed.

We may also telephone you after the 4-month visit to ask some questions about how you are finding being involved in the study. This will give you a chance to give some feedback on your experiences as well as your views on the care your friend/relative is receiving. Not all carers will receive a phone call. We aim to call 30 carers who have agreed to take part. We will offer you a £30 shopping voucher for your time and inconvenience in taking part in the telephone interview.

For peer review only - http://bmjopen.bmj.com/site/about/guidelines.xhtml

Timeline of visits

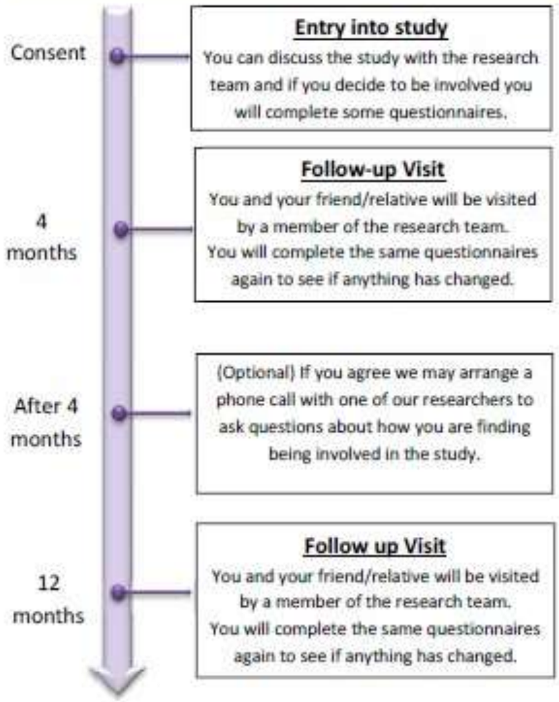

How will I know which treatment my friend/relative going to have?

In the FEMuR III study patients will be split into two groups at random:

- One group will receive normal NHS care
- The other group will receive the enhanced rehabilitation package

In the FEMuR III study participants are equally as likely to be in the group receiving normal NHS care as in the group receiving the enhanced programme. The healthcare team will let you know which group your friend/relative is in as soon as possible.

What are the benefits and risks of taking part?

We are not sure whether routine NHS care or the enhanced therapy programme is best but we anticipate that both will aid patient recovery following surgery.

We do not foresee any significant risks involved in taking part in FEMuR III, although the assessments will take up some time. It is also possible that the enhanced rehabilitation programme will take up more of your time if your friend/relative receives additional therapy sessions that you may have to support them with.

We hope that the results from the study will help doctors, therapists, patients and their carers in the future when making decisions about treatment.

What are the alternatives for treatment?

Patients recovering from hip fracture repair will get standard NHS treatment, though this may vary in different areas. In this study every patient will get standard NHS treatment even if they are in the enhanced rehabilitation group. Currently, there are no other alternative treatment programmes available.

Do I have to take part?

No, taking part is voluntary. It is up to you to decide whether or not you want to take part. If you choose to take part you can also choose to stop at any time without giving a reason. This will not affect the care received by your friend/relative or how you are treated.

What happens if I change my mind?

If at any point you decide to stop taking part in the study we will not collect any further information from you. We will use any study information collected up until the time you stop taking part.

Will my participation be kept confidential?

Yes. All information collected about you during the course of the study will be handled according to relevant ethical and legal requirements. Your personal information will be kept strictly confidential and will only be accessed by people working on the study, or working to ensure the study is being run correctly.

You will be given a study number, which will be used along with your initials to identify you on each paper form. Your full name and telephone number will be included on your consent form and a copy of this will be sent to the study team at the coordinating centre for the study, the Liverpool Clinical Trials Centre (LCTC.) There may be instances (depending on your local NHS,) when a copy of your contact details will need to be sent to other

For peer review only - http://bmjopen.bmj.com/site/about/guidelines.xhtml

locations or Universities within your local area to arrange your follow up visits. Only members of the FEMUR III team will be given access to your contact details, they will be held securely and destroyed after your final follow-up.We will ask for your telephone number so that we can contact you for the telephone interviews during the study. Your telephone number will not be used for anything else without your consent.

The University of Liverpool is the sponsor for this study based in the United Kingdom. The University of Liverpool along with Bangor University will be using information from you in order to undertake this study and act as the joint data controllers for this study. This means that both joint data controllers are responsible for looking after your information and using it properly. The University of Liverpool will keep identifiable information about you for a maximum of 25 years after the study has finished. Arrangements for confidential destruction will then be made.

Your rights to access, change or move your information are limited, as we need to manage your information in specific ways in order for the research to be reliable and accurate. If you withdraw from the study, we will keep the information about you that we have already obtained. To safeguard your rights, we will use the minimum personally-identifiable information possible.

You can find out more about how we use your information in the "**How we use your information**" section on the trial website Femur3study.co.uk

Your friend/relative's NHS hospital and/or community health team will collect information from you for this research study in accordance with our instructions.

The only people working on the studywho will have access to information that identifies you will be people who need to confirm your participation in the study, contact you after 4 months to ask you questions about taking part in this study or audit the data collection process. The people who analyse the information will not attempt to identify you or find out your name or contact details.

Your friend/relative's NHS hospital and/or community health team will keep identifiable information about you from this study for up to a maximum of 25 years after the study has finished.

Additional information about future research:

When you agree to take part in a research study, the information you give may be provided to researchers running other research studies in this organisation and in other organisations. These organisations may be universities, NHS organisations or companies involved in health and care research in this country or abroad. Your information will only be used by organisations and researchers to conduct research in accordance with the UK Policy Framework for Health and Social Care Research.

This information will not identify you and will not be combined with other information in a way that could identify you. The information will only be used for the purpose of health and care research, and cannot be used to contact you or to affect your care. It will not be used to make decisions about future services available to you, such as insurance.

What will happen to the results of the study?

It is intended that the results of the study will be presented at conferences and published in medical journals so that we can explain to the medical, nursing and therapies' community what our research results have shown. Confidentiality will be ensured at all times and you will not be identified in any publication.

What if there is a problem?

If you have a concern about any aspect of this study, you should ask to speak with one of the research team who will do their best to answer your questions.

If at any time during the study you feel distressed or anxious please speak to your research team, a therapist or contact your GP.

If you wish to make a formal complaint, you can do this through the NHS Complaints Procedure. Details can be obtained from the hospital.

Every care will be taken in the course of this clinical study. However, in the unlikely event that you are harmed by taking part in this research project, compensation may be available and you may have to pay your related legal costs. The University of Liverpool holds insurance against claims from participants for

For peer review only - http://bmjopen.bmj.com/site/about/guidelines.xhtml

harm caused by their participation in this clinical trial. Participants may be able to claim compensation if they can prove that the University of Liverpool has been negligent. However, if you are harmed and this is due to someone's negligence in the NHS, then you may have grounds for a legal action for compensation against the NHS, but you may have to pay for your legal costs. The normal NHS complaints procedures should be available to you. The study sponsor accepts no liability for negligence on part of your NHS organisation's employees.

Additional information

The University of Liverpool is responsible for managing this study; they have asked that the day to day running of the study is carried out by the Liverpool Clinical Trial Centre (LCTC)part of the University of Liverpool. Additional support is provided by other researchers from participating universities (the study team).

This study is funded by the National Institute for Health Research's Health Technology Assessment programme (ref: 16/167/09).

The study has been reviewed by the National Institute for Health Research (NIHR), Health Research Authority and the National Research Ethics Service Committee. Tyne and Wear South reviewed the study and given approval for it to take place.

The views expressed are those of the author(s) and not necessarily those of the NHS, the NIHR or the Department of Health.

Thank you for reading this information sheet.

Contacts for further information

If you would like more information or have any questions about the FEMuR III study please talk to:

Principal Investigator: <PI name to go here>

Research Nurse: <RN name to go here>

Telephone: <Hospital contact number to go here>

Or visit the website: Femur3study.co.uk

If you wish to discuss the study with someone independent of the research team you can contact the local NHS Patient Advice and Liaison Service (PALS) or local equivalent on: <<Local PALS or equivalent telephone number to go here>>

.

Fracture in the Elderly Multidisciplinary Rehabilitation III

<<Local NHS Logo to go here>>

<Trust/Site address 1>
<Trust/Site address 2>
<Trust/Site address 3>
<postcode>
Tel: <telephone number>

Carer Participant Consent Form

To be completed by the Researcher:			
Site Name:			
Carer Study Number		Carer Initials:	
Participant Study Number			

To be completed by the carer:

Once you have read and understood each statement please enter your initials in each box	Initial
Example: I confirm that I have read and understand the Participant Information Sheet.	JS
1. I have read and understood the information sheet for this study. I have had the opportunity to ask questions and have had these answered satisfactorily.	
2. I understand that participation is voluntary and that I am free to withdraw from the study at any time, without giving a reason, and without my care or legal rights being affected.	
3. I understand that my data will be retained for a maximum of 25 years at site and by the Liverpool Clinical Trials Centre (LCTC) part of the University of Liverpool and that they will be stored in a confidential manner.	
4. I give permission for a copy of my consent form which will include my name and telephone number to be sent to members of the FEMUR III team (where it will be kept in a secure location), to allow confirmation that my consent was given.	
5. I understand that any data collected during the study may be looked at by authorised individuals from the study team and those listed under "Additional information" (NHS organisation, sponsor and regulatory authorities). I give permission for these individuals to have access to my records.	
6. I agree to allow information or results arising from this study to be used in future healthcare and/or medical research in a pseudo-anonymised form.	
7. I agree to take part in the above study.	
**The statements below are OPTIONAL** ***(you can still participate even if you only agree to the statements above):***	
8. I agree that I may be contacted in the future in relation to this or other related studies.	
9. I agree to being contacted by a study researcher to conduct a qualitative interview and for the interview to be recorded. Telephone number: (if you agree to this statement provide your details below):	

Your full name (please print):			
Your signature:		Date:	

To be completed by the Researcher (after carer has completed the form):			
Researcher full name (please print):			
Researcher signature:		Date:	

FEMuR III Carer PISC V4.0 19/09/2019
IRAS Project ID: 246828

For peer review only - http://bmjopen.bmj.com/site/about/guidelines.xhtml

FEMuR III SPIRIT Checklist

SPIRIT

STANDARD PROTOCOL ITEMS: RECOMMENDATIONS FOR INTERVENTIONAL TRIA

SPIRIT 2013 Checklist: Recommended items to address in a clinical trial protocol and related documents*

Section/item	Item No	Description	Addressed on page number
**Administrative information**			
Title	1	Descriptive title identifying the study design, population, interventions, and, if applicable, trial acronym	1
Trial registration	2a	Trial identifier and registry name. If not yet registered, name of intended registry	1,3
	2b	All items from the World Health Organization Trial Registration Data Set	Appendix 3
Protocol version	3	Date and version identifier	19
Funding	4	Sources and types of financial, material, and other support	31
Roles and responsibilities	5a	Names, affiliations, and roles of protocol contributors	1,2,26
	5b	Name and contact information for the trial sponsor	30
	5c	Role of study sponsor and funders, if any, in study design; collection, management, analysis, and interpretation of data; writing of the report; and the decision to submit the report for publication, including whether they will have ultimate authority over any of these activities	29-31
	5d	Composition, roles, and responsibilities of the coordinating centre, steering committee, endpoint adjudication committee, data management team, and other individuals or groups overseeing the trial, if applicable (see Item 21a for data monitoring committee)	30,31

For peer review only

Introduction

Background and rationale	6a	Description of research question and justification for undertaking the trial, including summary of relevant studies (published and unpublished) examining benefits and harms for each intervention	6,7
	6b	Explanation for choice of comparators	6,7
Objectives	7	Specific objectives or hypotheses	8
Trial design	8	Description of trial design including type of trial (eg, parallel group, crossover, factorial, single group), allocation ratio, and framework (eg, superiority, equivalence, noninferiority, exploratory)	9

Methods: Participants, interventions, and outcomes

Study setting	9	Description of study settings (eg, community clinic, academic hospital) and list of countries where data will be collected. Reference to where list of study sites can be obtained	9
Eligibility criteria	10	Inclusion and exclusion criteria for participants. If applicable, eligibility criteria for study centres and individuals who will perform the interventions (eg, surgeons, psychotherapists)	9,10
Interventions	11a	Interventions for each group with sufficient detail to allow replication, including how and when they will be administered	10-12
	11b	Criteria for discontinuing or modifying allocated interventions for a given trial participant (eg, drug dose change in response to harms, participant request, or improving/worsening disease)	11,12
	11c	Strategies to improve adherence to intervention protocols, and any procedures for monitoring adherence (eg, drug tablet return, laboratory tests)	12,18-21
	11d	Relevant concomitant care and interventions that are permitted or prohibited during the trial	10,11

For peer review only - http://bmjopen.bmj.com/site/about/guidelines.xhtml

Outcomes	12	Primary, secondary, and other outcomes, including the specific measurement variable (eg, systolic blood pressure), analysis metric (eg, change from baseline, final value, time to event), method of aggregation (eg, median, proportion), and time point for each outcome. Explanation of the clinical relevance of chosen efficacy and harm outcomes is strongly recommended	12,13
Participant timeline	13	Time schedule of enrolment, interventions (including any run-ins and washouts), assessments, and visits for participants. A schematic diagram is highly recommended (see Figure)	34,35
Sample size	14	Estimated number of participants needed to achieve study objectives and how it was determined, including clinical and statistical assumptions supporting any sample size calculations	13,14
Recruitment	15	Strategies for achieving adequate participant enrolment to reach target sample size	14,15

Methods: Assignment of interventions (for controlled trials)

Allocation:

Sequence generation	16a	Method of generating the allocation sequence (eg, computer-generated random numbers), and list of any factors for stratification. To reduce predictability of a random sequence, details of any planned restriction (eg, blocking) should be provided in a separate document that is unavailable to those who enrol participants or assign interventions	15
Allocation concealment mechanism	16b	Mechanism of implementing the allocation sequence (eg, central telephone; sequentially numbered, opaque, sealed envelopes), describing any steps to conceal the sequence until interventions are assigned	15
Implementation	16c	Who will generate the allocation sequence, who will enrol participants, and who will assign participants to interventions	15
Blinding (masking)	17a	Who will be blinded after assignment to interventions (eg, trial participants, care providers, outcome assessors, data analysts), and how	15,16

	17b	If blinded, circumstances under which unblinding is permissible, and procedure for revealing a participant's allocated intervention during the trial	15,16

Methods: Data collection, management, and analysis

Data collection methods	18a	Plans for assessment and collection of outcome, baseline, and other trial data, including any related processes to promote data quality (eg, duplicate measurements, training of assessors) and a description of study instruments (eg, questionnaires, laboratory tests) along with their reliability and validity, if known. Reference to where data collection forms can be found, if not in the protocol	12,13
	18b	Plans to promote participant retention and complete follow-up, including list of any outcome data to be collected for participants who discontinue or deviate from intervention protocols	32-34
Data management	19	Plans for data entry, coding, security, and storage, including any related processes to promote data quality (eg, double data entry; range checks for data values). Reference to where details of data management procedures can be found, if not in the protocol	16,17
Statistical methods	20a	Statistical methods for analysing primary and secondary outcomes. Reference to where other details of the statistical analysis plan can be found, if not in the protocol	16,17
	20b	Methods for any additional analyses (eg, subgroup and adjusted analyses)	17-21
	20c	Definition of analysis population relating to protocol non-adherence (eg, as randomised analysis), and any statistical methods to handle missing data (eg, multiple imputation)	16,17

Methods: Monitoring

Data monitoring	21a	Composition of data monitoring committee (DMC); summary of its role and reporting structure; statement of whether it is independent from the sponsor and competing interests; and reference to where further details about its charter can be found, if not in the protocol. Alternatively, an explanation of why a DMC is not needed	21,31
	21b	Description of any interim analyses and stopping guidelines, including who will have access to these interim results and make the final decision to terminate the trial	16,31
Harms	22	Plans for collecting, assessing, reporting, and managing solicited and spontaneously reported adverse events and other unintended effects of trial interventions or trial conduct	21
Auditing	23	Frequency and procedures for auditing trial conduct, if any, and whether the process will be independent from investigators and the sponsor	21
**Ethics and dissemination**			
Research ethics approval	24	Plans for seeking research ethics committee/institutional review board (REC/IRB) approval	21
Protocol amendments	25	Plans for communicating important protocol modifications (eg, changes to eligibility criteria, outcomes, analyses) to relevant parties (eg, investigators, REC/IRBs, trial participants, trial registries, journals, regulators)	21
Consent or assent	26a	Who will obtain informed consent or assent from potential trial participants or authorised surrogates, and how (see Item 32)	14,15
	26b	Additional consent provisions for collection and use of participant data and biological specimens in ancillary studies, if applicable	N/A
Confidentiality	27	How personal information about potential and enrolled participants will be collected, shared, and maintained in order to protect confidentiality before, during, and after the trial	14,15, Appendices 1&2
Declaration of interests	28	Financial and other competing interests for principal investigators for the overall trial and each study site	31

For peer review only

Access to data	29	Statement of who will have access to the final trial dataset, and disclosure of contractual agreements that limit such access for investigators	22
Ancillary and post-trial care	30	Provisions, if any, for ancillary and post-trial care, and for compensation to those who suffer harm from trial participation	31
Dissemination policy	31a	Plans for investigators and sponsor to communicate trial results to participants, healthcare professionals, the public, and other relevant groups (eg, via publication, reporting in results databases, or other data sharing arrangements), including any publication restrictions	22
	31b	Authorship eligibility guidelines and any intended use of professional writers	22
	31c	Plans, if any, for granting public access to the full protocol, participant-level dataset, and statistical code	22
**Appendices**			
Informed consent materials	32	Model consent form and other related documentation given to participants and authorised surrogates	Appendices 1&2
Biological specimens	33	Plans for collection, laboratory evaluation, and storage of biological specimens for genetic or molecular analysis in the current trial and for future use in ancillary studies, if applicable	N/A

*It is strongly recommended that this checklist be read in conjunction with the SPIRIT 2013 Explanation & Elaboration for important clarification on the items. Amendments to the protocol should be tracked and dated. The SPIRIT checklist is copyrighted by the SPIRIT Group under the Creative Commons "Attribution-NonCommercial-NoDerivs 3.0 Unported" license.

For peer review only
